# Clemastine Fumarate Attenuates Myocardial Ischemia Reperfusion Injury Through Inhibition of Mast Cell Degranulation

**DOI:** 10.3389/fphar.2021.704852

**Published:** 2021-08-27

**Authors:** Shuqi Meng, Xiaotong Sun, Zhaodong Juan, Mingling Wang, Ruoguo Wang, Lina Sun, Yaozu Li, Anran Xin, Shuping Li, Yao Li

**Affiliations:** ^1^ Shandong Provincial Medicine and Health Key Laboratory of Clinical Anesthesia, School of Anesthesiology, Weifang Medical University, Weifang, China; ^2^ Department of Pain, Affiliated Hospital of Weifang Medical University, Weifang, China

**Keywords:** myocardial ischemia reperfusion injury, mast cell, clemastine fumarate, degranulation, pretreatment

## Abstract

Mast cell (MC) activation is associated with myocardial ischemia reperfusion injury (MIRI). Suppression of MC degranulation might be a target of anti-MIRI. This study aimed to determine whether clemastine fumarate (CLE) could attenuate MIRI by inhibiting MC degranulation. A rat ischemia and reperfusion (I/R) model was established by ligating the left anterior descending coronary artery for 30 min followed by reperfusion for 120 min. Compound 48/80 (C48/80) was used to promote MC degranulation. The protective effect of CLE by inhibiting MC degranulation on I/R injury was detected by cardiac function, 2,3,5-triphenyl tetrazolium chloride (TTC) staining, hematoxylin-eosin (HE) staining, arrhythmia, and myocardial enzyme detection. Inflammatory factor mRNA levels, such as TNF-α, interleukin (IL)-1β, and IL-6, were detected. Cultured RBL-2H3 mast cells were pretreated with CLE and subjected to C48/80 treatment to determine whether CLE suppressed MC degranulation. Degranulation of MCs was visualized using tryptase release, Cell Counting Kit-8 (CCK-8), and cell toluidine blue (TB) staining. RBL cells were conditionally cultured with H9C2 cells to explore whether CLE could reverse the apoptosis of cardiomyocytes induced by MC degranulation. Apoptosis of H9C2 cells was detected by CCK-8, the LDH Cytotoxicity Assay Kit (LDH), TUNEL staining, and protein expression of BAX and Bcl-2. We found that CLE pretreatment further inhibited cardiac injury manifested by decreased infarct size, histopathological changes, arrhythmias, MC degranulation, and myocardial enzyme levels, improving cardiac function compared with that in the I/R group. C48/80 combined with I/R exacerbated these changes. However, pretreatment with CLE for C48/80 combined with I/R significantly reversed these injuries. In addition, CLE pretreatment improved the vitality of RBL cells and reduced tryptase release *in vitro*. Similarly, the supernatant of RBL cells pretreated with CLE decreased the cytotoxicity, TUNEL-positive cell rate, and BAX expression of conditioned H9C2 cells and increased the cell vitality and expression of Bcl-2. These results suggested that pretreatment with CLE confers protection against I/R injury by inhibiting MC degranulation.

## Introduction

The dominant reasons for morbidity and mortality in patients who undergo noncardiac surgery are perioperative cardiac complications such as myocardial ischemia and infarction (Ibacache et al., 2012). Ischemia of the heart initiates a complex and interrelated sequence of events leading to myocardial dysfunction and damage (Collino et al., 2012). Early and fast restoration of blood flow is used to prevent further damage to the myocardial tissue. Unfortunately, restoring blood flow to the ischemic myocardium can aggravate injury called reperfusion injury (Frank et al., 2012). At present, treatment of cardiac ischemia and reperfusion (I/R) injury is mainly supportive therapy because no effective target-oriented therapy has been validated. The current research focus is to explore the underlying mechanism of I/R to improve clinical treatment for preventing or ameliorating its damage (He et al., 2019).

Mast cells (MCs) are a multieffector cell type and are best known for their characteristics in instant hypersensitivity and chronic allergy (Liu et al., 2014). Furthermore, many recent indications have shown that MCs are involved in I/R damage in many organs, such as the heart and brain (Yang et al., 2014). Cardiac MCs (CMCs) are distributed around the microvasculature and serve as instant reactors in the initial period of I/R occurrence through degranulation and releasing media comprising tryptase, chymase, histamine, etc. (Xiong et al., 2020). A study indicated that patients with acute ST-elevation myocardial infarction (STEMI) have poor myocardial reperfusion and poor cardiac function related to high tryptase levels. Therefore, prevention of myocardial infarction and improvement of myocardial reperfusion might benefit from inhibiting tryptase release after primary percutaneous coronary intervention (PCI), causing poor cardiac function (Chen et al., 2014). In addition, the release of renin from MCs causes initiation of the local cardiac renin-angiotensin system (RAS), which is responsible for norepinephrine (NE) secretion and results in arrhythmia. However, the cardioprotective effect of anti-RAS can be achieved by sequential activation of MC Gi-coupled receptors, protein kinase C-ε (PKCε), and mitochondrial aldehyde dehydrogenase-type 2 (ALDH2) (Marino and Levi, 2018). Furthermore, experimental research has shown that ketotifen and low-dose carvedilol, as MC stabilizers, can significantly attenuate myocardial I/R injury (Jaggi et al., 2007).

Clemastine fumarate (CLE), a second-generation histamine H1 receptor (H1R) blocker, is often used clinically to treat various allergic diseases caused by histamine. Histamine H1R antagonists have been shown to suppress MC release of histamine and proinflammatory cytokines such as IL-3, IL-6, IL-8, and TNF-α and inhibit MC-reliant leukocyte recruitment in the process of I/R (Singh and Saini, 2003). Our previous study indicated that CLE offers a cardioprotective effect during myocardial ischemia reperfusion injury (MIRI) in cardiomyocytes (Yuan et al., 2020). However, it is unclear whether the cardioprotective effect of CLE in MIRI is related to MC activation. In this study, we used a rat I/R model and a cardiomyocyte conditional culture model to explore whether the activation of CMCs and the release of their products were involved in the protective activity of CLE in I/R injury.

## Materials and Methods

### Materials and Reagents

CLE was purchased from Huarun Shuanghe Limin Pharmaceutical Co., Ltd. (2 ml: 2 mg; Jinan, China). Dulbecco’s modified Eagle’s medium (DMEM, SH30243.01) was obtained from HyClone (Utah, United States). Fetal bovine serum (FBS, 16140089) was obtained from Gibco, United States. Cell Counting Kit-8 (BS350B) was obtained from Biosharp Life Sciences (Beijing, China). An LDH Cytotoxicity Assay Kit (C0017) was obtained from Beyotime Biotechnology (Shanghai, China). Compounds 48/80 (C2313-100 mg), lα-N- benzoyl-L-arginine-*p*-nitroanilide (B4875-1 g) and 2,3,5-triphenyl tetrazolium chloride (10838000 10–1 g) were purchased from Sigma, United States A hematoxylin-eosin staining kit (G1121-4) and toluidine blue staining kit (G3661) were obtained from Solarbio Science and Technology Co., Ltd. (Beijing, China). LDH (A020-2-2) and CK-MB (H197-1) enzyme-linked immunosorbent assay (ELISA) kits were purchased from Jiancheng Bioengineering Institute of Nanjing, China. A TUNEL Apoptosis Detection Kit III and FITC (MK1013-100) were obtained from Boster Biological Technology (Wuhan, China). Antibodies against PKCδ (ab182126, RRID: AB_304316), H1R (ab154158, RRID: AB_1523787), and tryptase (ab2378, RRID: AB_303023) were obtained from Abcam (Shanghai, China). Antibodies against BAX (Ca#50599-2-Ig, RRID: AB_2061561), Bcl-2 (Ca#26593-1-AP, RRID: AB_2818996), and GAPDH (Cat#10494-1-AP, RRID: AB_2263076) were purchased from Proteintech (Wuhan, China). Goat anti-rabbit immunoglobulin G (IgG) (H + L), horseradish peroxidase (HRP) (MultiSciences, 70-GAM0072), and goat anti-mouse (MultiSciences, 70-GAR0072) IgG (H + L) HRP were purchased from Hangzhou, China. FastPure Cell/Tissue Total RNA Isolation Kit V2 (RC101), HiscriptⅡQ RT SuperMix for qPCR (+gDNA wiper, R233-01) and ChamQ SYBR qPCR Master Mix (Q711-02/03) were purchased from Vazyme (Nanjing, China).

### Myocardial Ischemia Reperfusion Model in Rats

Animal handling and experimental procedures were approved by Committee on the Ethics of Animal Experiments of Weifang Medical University (Weifang, China). Male Sprague–Dawley (SD) rats weighing 200–250 g were purchased from Jinan Pengyue Laboratory Animal Breeding Co., Ltd. (Jinan, China) and housed individually in cages. All the rats were raised at 22°C–25°C and 45–55% humidity for 1 week before the test. The rats were anesthetized with 1% Nembutal sodium (50 mg/kg) intraperitoneal injection and tracheal ventilation before opening the chest (Xiong et al., 2020). Then, a thoracotomy was performed between the third and fourth intercostal space to expose the heart. A 7–0 silk suture slipknot was used to ligate the left anterior descending (LAD) coronary artery for 30 min of ischemia followed by 120 min of reperfusion. We observed cyanosis of the heart and abnormal wall motion during LAD ligation. ST segment elevation recorded by the LabChart 7.2 multichannel physiological signaling system. After reperfusion, the above phenomenon improved, including the ST elevated segment recovered by more than 50%. The above marks the successful establishment of the MIRI model.

### Animal Grouping

The rats were randomly divided into five groups (*n* = 6 per group): in the sham-operated group (Group S), rats injected with normal saline (10 ml/kg, i. p.) underwent surgery except for LAD ligation for 30 min, and the same volume of normal saline (1 ml/kg, i. v.) was administered as a reagent solvent control (Gan et al., 2015). In the I/R group, rats injected with normal saline (10 ml/kg, i. p.) underwent surgery for ischemia (30 min), followed by reperfusion (2 h), plus normal saline (1 ml/kg, i. v.) was administered 5 min immediately before reperfusion. In the CLE + I/R group, rats were injected with CLE (5 mg/kg, i. p.) dissolved in normal saline (10 ml/kg) 4 h prior to surgery for ischemia (30 min), followed by reperfusion (2 h), plus normal saline (1 ml/kg, i. v.) was administered 5 min immediately before reperfusion. In the C48/80 + I/R group, rats injected with normal saline (10 ml/kg, i. p.) underwent surgery for ischemia (30 min), followed by reperfusion (2 h), plus C48/80 (0.5 mg/kg, i. v.) dissolved in normal saline (1 ml/kg) was administered 5 min immediately before reperfusion. In the CLE + C48/80 + I/R group, rats injected with CLE (5 mg/kg, i. p.) underwent surgery for ischemia (30 min), followed by reperfusion (2 h), plus C48/80 (0.5 mg/kg, i. v.) was administered. Throughout the surgery, the body temperature of the rats was maintained at 37°C using a heated pad.

### Evaluation of Cardiac Function

The cardiac function of the rats was measured by a Vevo 3100 LT Imaging System after 2 h of reperfusion. With M-mode, the cardiac function parameters left ventricular ejection fraction (LVEF) and left ventricular fraction shortening (LVFS) were obtained. Additionally, parameters such as the left ventricular end-systolic diameter (LVESD), left ventricular end-diastolic diameter (LVEDD), left ventricular end-systolic volume (LVESV), and left ventricular end-diastolic volume (LVEDV) were measured.

### Evaluation of Electrocardiogram

A standard limb lead II ECG was recorded by a LabChart 7.2 multichannel physiological signaling system. LabChart 7.2 was set to sample frequency 1 K, range 5 mV, and low-pass amplifier filtering 500 HZ. We recorded ECG after stabilization for 10 min, after ischemia for 10 min, and after reperfusion for 10 min. The arrhythmia severity scoring system was used to assess the severity of arrhythmia after LAD ligation (Polshekan et al., 2019). We also assessed the incidence of arrhythmia after LAD ligation as well as the persistence of ventricular tachycardia (VT) and ventricular fibrillation (VF) during reperfusion.

### Analysis of Ischemic Area

At the end of the experiments, the hearts were frozen and cut into five cross-sections (2 mm) and stained with 1% 2,3,5-triphenyl tetrazolium chloride (TTC) for 30 min at 37°C. The sections were kept in a 4% aqueous solution of formaldehyde for 24 h. The infarcted area (IA; stained white) and noninfarcted area (NIA; stained red) of the left ventricle were quantified by Image-Pro 6.0. Moreover, infarct size (IS) was derived from the formula IS = IA/(IA + NIA)%.

### Hematoxylin-Eosin Staining

The left ventricle was fixed in 4% paraformaldehyde for 24 h and gradient dehydration of 10, 20, and 30% sucrose solution. The tissues were cut into 3–4 μm sections after OCT compound embedding. The sections were stained with HE according to the manufacturer’s instructions. Finally, the sections were viewed and photographed under a microscope (BX-53, Olympus Corporation, Tokyo, Japan).

### Enzyme-Linked Immunosorbent Assay

Elevated LDH and CK-MB commonly indicate myocardial cell injury. Rat serum was collected from each experimental group. The LDH and CK-MB levels were detected using a commercial ELISA kit per the instructions. Light absorbance was measured at 450 nm by a spectrophotometer (Thermo Scientific, Waltham, MA, United States).

### Toluidine Blue Staining

The preparation of myocardial tissue was similar to HE staining but stained with TB staining in accordance with the manufacturer’s instructions. Then, a BX-53 microscope (Olympus Corporation, Tokyo, Japan) was used to observe and count the number of intact (IMCs) and degranulated (DMCs) MCs. The degranulation rate of MC was calculated by the formula MCD = DMCs/(IMCs + DMCs) ×100% (Xiong et al., 2020). Moreover, RBL-2H3 cells were also stained with TB and, before staining, fixed with 95% ethanol according to the instructions.

### Assay for Tryptase Activity in Serum

After heart reperfusion, one hundred microliters of serum was collected and immediately incubated to determine the enzyme activity of tryptase. After 72 h of incubation with 100 μl of 0.8 mmol/L BAPNA at 37°C, the light absorbance of the nitroaniline product was measured at 405 nm with a spectrophotometer (Yu et al., 2018).

### RNA Isolation and qRT–PCR

Total RNA was extracted from cardiac tissues by a FastPure Cell/Tissue Total RNA Isolation Kit V2, and cDNA was synthesized by reverse transcription using HiScript II Q RT SuperMix for qPCR (+gDNA wiper). Ten microliters of PCR master mix consisted of 4.8 μl cDNA template (15 ng/μl), 5 µl 2×ChamQ SYBR qPCR Master Mix, and 0.2 µl gene-specific primers (10 μM). Then, the reactions were performed using the LightCycler 480 II (Roche, Indianapolis, IN, United States) to conduct qPCR under the following conditions: predenaturation (5 min at 95°C), followed by 40 cycles at 95°C for 10 s and 60°C for 30 s. The primer sequences of TNF-α (forward:5-CGAGTGACAAGCCCGTAGCC‐3 and reverse:5-GGATGAACACGCCAGTCGCC-3), IL-6 (forward:5-CTCTCCGCAAGAGACTTCCA‐3 and reverse:5-TCTCC-TCTCCGGACTTGTGAA‐3), IL-1β (forward:5-GGGATG-ATGACGACGACCTGC‐3 and reverse:5-CCACTTGTT-GGCTTATGTT‐3), H1R (forward:5-CCGGACCACAG-ACTCAGACA-3 and reverse:5-GAGTGTGAGCGGAG-CCTCTT-3), PKCδ (forward:5-GTCACCATCTTCCA-GAAAGAACG-3 and reverse:5-CTTGCCAT AGGTCCAGT-TGTTG-3) and GAPDH (forward: 5-GTT​ACC​AGG​GCT​GCC​TTC​TC-3 and reverse: 5-CTCGT GGTTCA CACCCATCA-3). We purchased the primers from BGI (Shanghai, China), and the relative mRNA levels were standardized relative to GAPDH.

### Cell Culture

H9C2 cardiomyocytes and RBL-2H3 mast cells were obtained from Procell Life Science and Technology Co., Ltd. (Wuhan, China) and cultured with DMEM supplemented with 10% FBS and 100 units/ml penicillin/streptomycin at 37°C in 5% CO_2_ with 95% humidity. The density of the cells was maintained at 1 × 10^6^ cells/ml in 25 cm^2^ culture flasks.

### Cell Grouping

**FIGURE 1:**
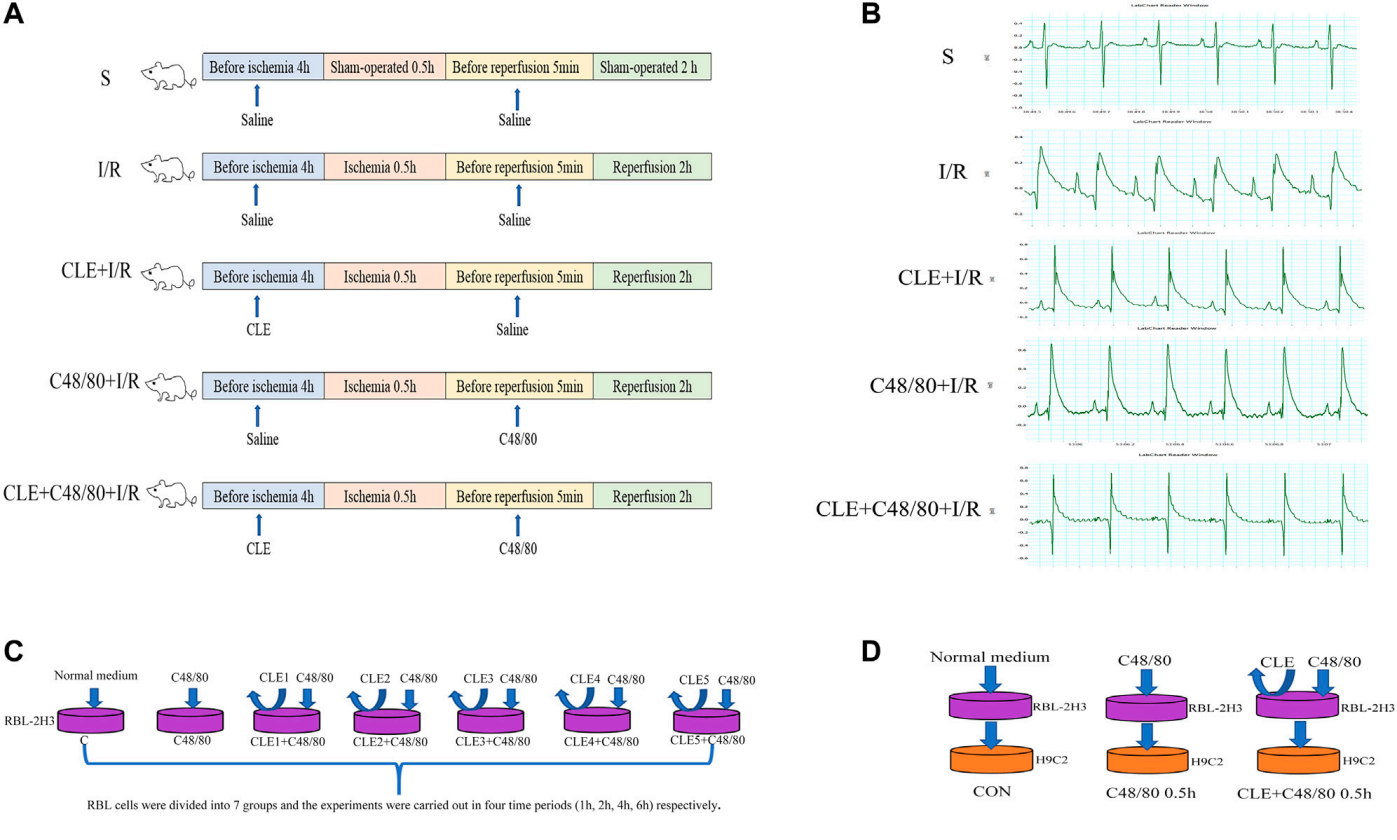
Schematic diagram of experimental design. **(A)** MIRI model and animal grouping **(B)** ECG after ischemia stabilization. **(C)** The effect of CLE on MCs degranulation model and experimental grouping. **(D)** RBL-2H3 and H9C2 co-culture model and experimental grouping.

To explore the optimal time and concentration of CLE for MC degranulation, RBL cells were divided into seven groups at four time periods (1, 2, 4, and 6 h), as shown in Figure 1. The blank control group (Group C) was cultured in a typical environment without any treatment. In the C48/80 group, RBL cells were stimulated by C48/80 (10 μg/ml) for 30 min. In the CLE1+C48/80 group, RBL cells were pretreated with CLE (1 ng/ml) at the four time periods before C48/80 (10 μg/ml) stimulation for 30 min. In the CLE2+C48/80 group, RBL cells were pretreated with CLE (10 ng/ml) at the four time periods before C48/80 (10 μg/ml) stimulation for 30 min. In the CLE3+C48/80 group, RBL cells were pretreated with CLE (100 ng/ml) at the four time periods before C48/80 (10 μg/ml) stimulation for 30 min. In the CLE4+C48/80 group, RBL cells were pretreated with CLE (1 μg/ml) at the four time periods before C48/80 (10 μg/ml) stimulation for 30 min. In the CLE5+C48/80 group, RBL cells were pretreated with CLE (10 μg/ml) at the four time periods before C48/80 (10 μg/ml) stimulation for 30 min.

CLE reversed the apoptosis of cardiomyocytes induced by MC degranulation. RBL cells were conditionally cultured with H9C2 cells and divided into three groups: the control group (Con group), in which untreated RBL cell supernatant was added to H9C2 cells for 0.5 h. In the C48/80 0.5 h group, RBL cells were treated with C48/80 (10 μg/ml) for 0.5 h, then supernatant was added to H9C2 cells for 0.5 h (Hara et al., 1999). In the CLE + C48/80 0.5 h group, RBL cells were treated with CLE for 2 h first, the supernatant containing CLE was discarded, and C48/80 (10 μg/ml) was added to RBL cells for 0.5 h; subsequently, this supernatant was added to H9C2 cells for 0.5 h.

### Measurement of Mast Cell Degranulation

RBL-2H3 cells were seeded into 96-well flat bottom culture plates at a density of 1×10^4^ cells/well and incubated for 24 h. Cells were treated with CLE (1 ng/ml-10 μg/ml) before C48/80 (10 μg/ml) stimulation for 30 min. Tryptase is the unique mediator of MC degranulation (Pejler et al., 2010). The released tryptase was obtained by centrifuging the cells at 2000 rpm for 10 min at 4°C. Supernatants (100 μl) were added to 100 μl of 0.8 mmol/lα-N-benzoyl-L-arginine-*p*-nitroanilide (BAPNA, Sigma, United States) in Tris buffer and incubated at 37°C for 72 h (Yu et al., 2018). C48/80 induces lethal degranulation of MCs. Therefore, Cell Counting Kit-8 (CCK-8) was used as one of the indicators for measuring MC degranulation (Xiang et al., 2001).

### Measurement of Cell Viability and Toxicity

Under sterile conditions, MC granules (MCGs) were prepared from RBL-2H3 cells by stimulation with C48/80 (10 μg/ml) for 30 min. H9C2 cells were seeded into 96-well flat bottom culture plates at a density of 1×10^4^ cells/well and incubated for 24 h. After exposure to MCGs for 0.5 h, cell viability was measured by CCK-8, and cytotoxicity was measured with an LDH Cytotoxicity Assay Kit according to the manufacturer’s instructions.

### Measurement of Cell Apoptosis

After conditional training, H9C2 cells were fixed with 4% paraformaldehyde for 30 min and washed with 0.01 M TBS three times for 2 min. According to the manufacturer’s instructions (TUNEL Apoptosis Detection Kit, MK-1013), 20 μl of labeling buffer (1 μl each of TdT and BIO-d-UTP and 18 μl of buffer mixed together) was added to H9C2 cells at 37°C for 2 h. Then, 0.01 M TBS was used to wash three times for 2 min. After blocking for 2 h at room temperature, H9C2 cells were fluorescently labeled with SABC solution for 30 min. In addition, nuclei were visualized by DAPI staining if necessary. TUNEL-positive cardiomyocytes fluoresced green and were observed by a BX-53 microscope (Olympus Corporation, Tokyo, Japan). The apoptosis rate was equal to the percentage of TUNEL-positive cardiomyocytes compared to the total number of cells.

### Western Blot Analysis

After the experiment, myocardial tissue and H9C2 cells were homogenized in RIPA buffer (R0020, Solarbio, Beijing) at 4°C and centrifuged at 12,000 rpm, 4°C, and 20 min. The supernatant was obtained for proteins, the concentration of which was detected using a BCA protein assay kit (PC0020, Solarbio, Beijing). Protein was boiled for 5 min at 100°C and separated in 12% SDS–PAGE polyacrylamide gels, followed by transfer onto a PVDF membrane (Millipore, United States). The membranes were blocked with 5% nonfat dry milk for 2 h at room temperature and then incubated overnight at 4°C with primary antibodies against PKCδ (1:2,500), H1R (1:1,000), tryptase (1:500), Bcl-2 (1:1500) and BAX (1:8,000). GAPDH (1:10,000) was used as an internal reference. After washing with Tris-buffered saline with Tween 20 (TBST) three times, the membranes were incubated with the secondary antibody (1:6,000) for 2 h. Membranes were visualized with ECL (CWBIO, Beijing, China). The band density was measured by Image-Pro Plus software.

### Statistical Analysis

All the experimental data are expressed as the mean ± standard deviation (SD). Statistical analysis was processed using SPSS 20.0 statistical software. One-way ANOVA was used to compare multiple groups, and a *t* test was used for pairwise comparisons. A value of *p* < 0.05 was considered to indicate statistical significance.

## Results

### Pretreatment With Clemastine Fumarate Had a Protective Effect on MIRI and Reduced Serum Tryptase

**FIGURE 2:**
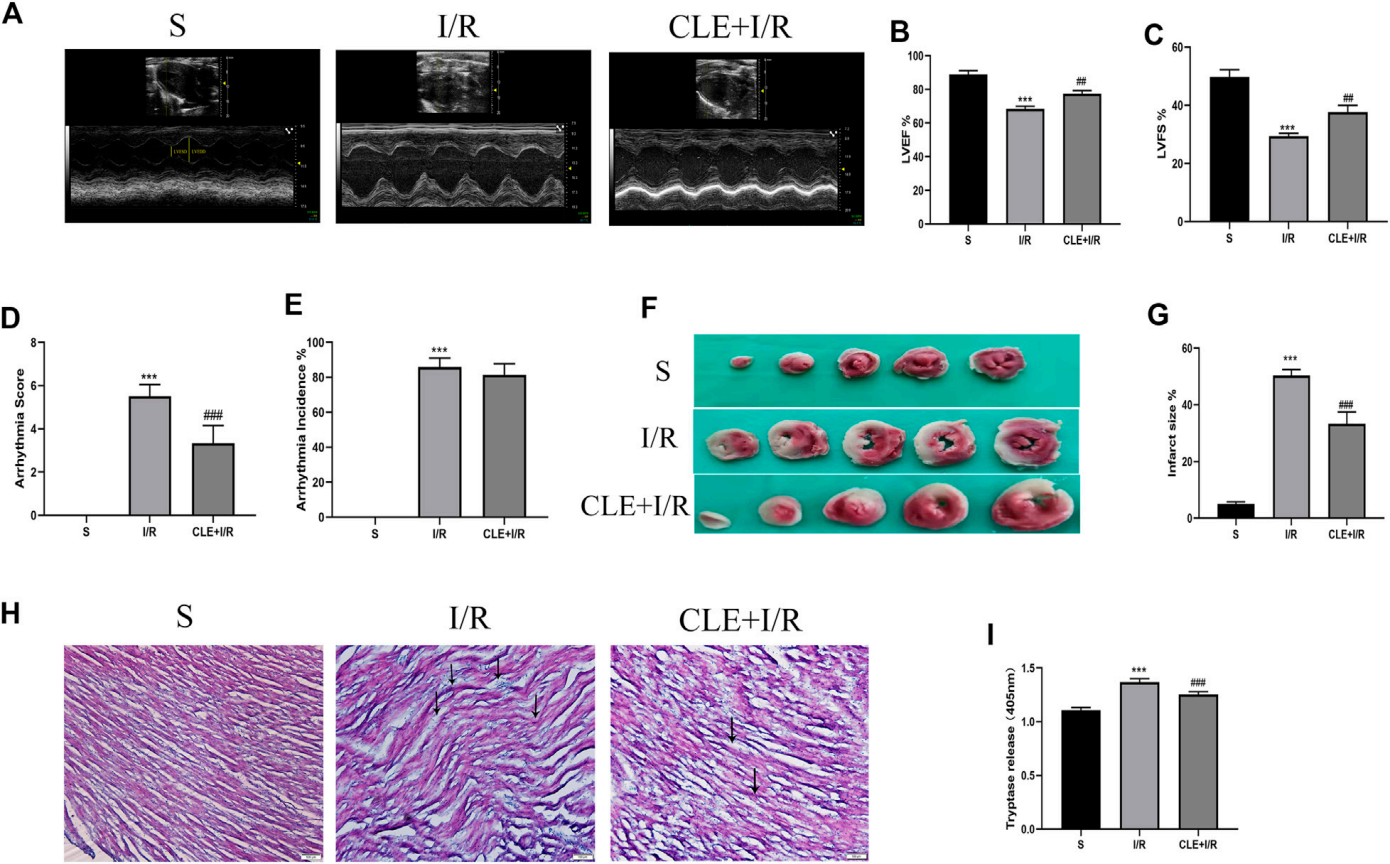
CLE pretreatment alleviated myocardial I/R injury and reduced the serum tryptase. **(A)** Cardiac function detected by M-mode echocardiography. The vertical yellow lines indicate the diameter of the left ventricle at the end of the systole or diastole stage. **(B)** Left ventricular ejection fraction (LVEF). **(C)** Left ventricular fraction shortening (LVFS). **(D)** Arrhythmia severity score. **(E)** Assessment of the incidence of arrhythmia. **(F)** Myocardial infarction area detected by TTC staining. **(G)** Infarct size%. **(H)** Cardiac pathological injury detected by HE staining (×100). The black arrows represent the obvious changes in myocardial tissue. **(I)** The release of tryptase in serum detected by BAPNA. *n* = 3–6 in each group. All data are the mean ± SD. ***p < 0.001 *vs.* the Sham group. ^##^ *p* < 0.01, ^###^ *p* < 0.001 *vs.* the I/R group.

To evaluate the effect of CLE preconditioning on MIRI, we detected cardiac contractile function, infarct size, histopathological structure, and electrocardiogram (ECG) changes. Compared with the sham group, the I/R injury group exhibited significantly reduced LVEF and LVFS, while pretreatment with CLE improved LVEF and LVFS (Figures 2A–C). ECG analysis showed that I/R injury increased the arrhythmia score and incidence of arrhythmia after LAD occlusion compared with the sham group, while CLE pretreatment decreased the arrhythmia score and had no effect on the incidence of arrhythmia (Figures 2D,E). Furthermore, representative images of TTC staining showed that I/R injury affected infarct sizes (IS) in heart issues (Figures 2F,G). Remarkable reductions in IS were observed in the CLE + I/R group compared with the I/R group. Similarly, CLE pretreatment improved wavy fibers and myocardial edema and reduced the infiltration of inflammatory cells resulting from I/R injury (Figure 2H). In addition, we found that the expression of serum tryptase in the I/R group was significantly increased compared with that in the sham group and that pretreatment with CLE decreased the expression of tryptase (Figure 2I).

### Clemastine Fumarate Inhibited Mast Cell Degranulation in MIRI

**FIGURE 3:**
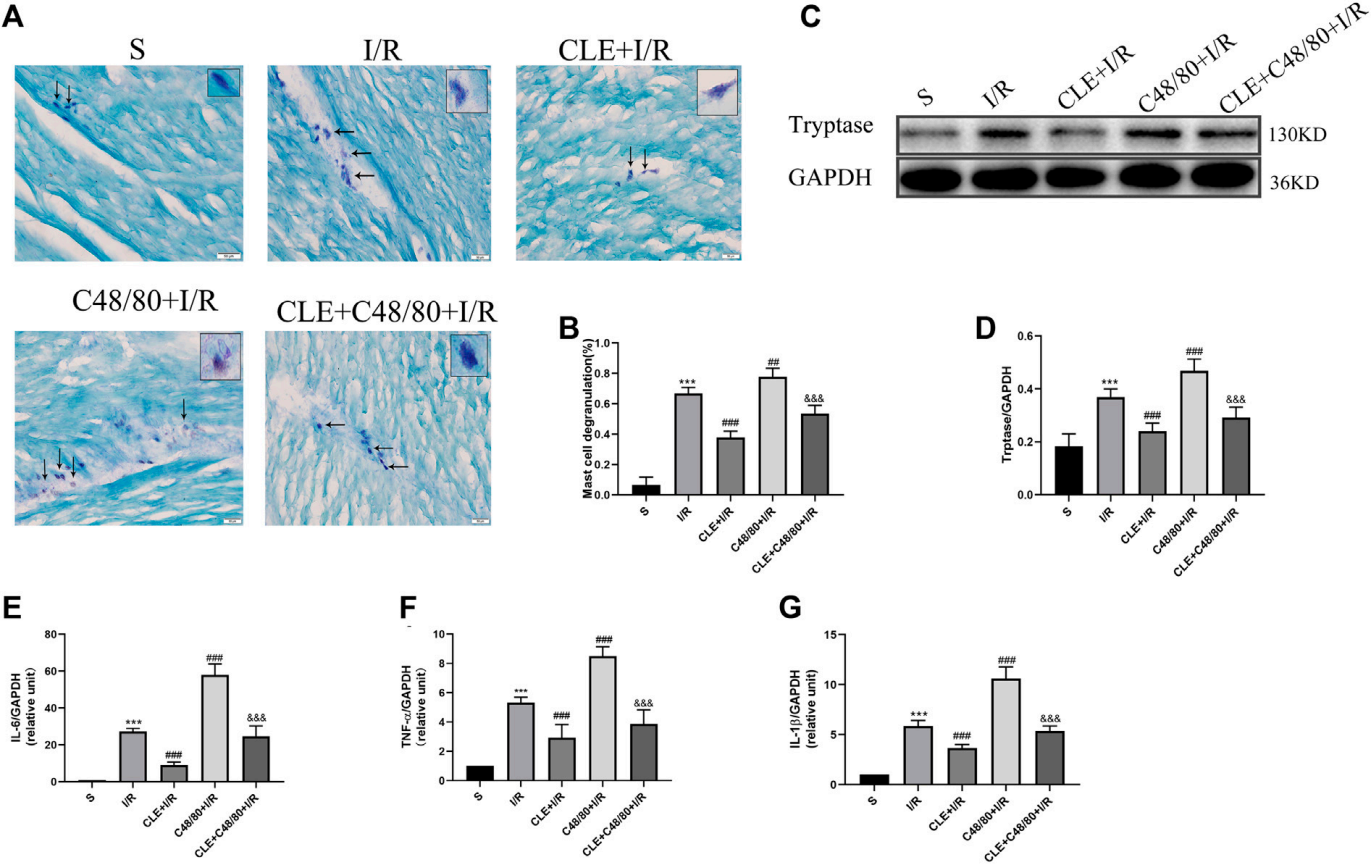
CLE pretreatment inhibited MCs degranulation on MIRI. **(A)** MC degranulation detected by TB staining (×200). The black arrows represent the typical morphology of MCs in different states. **(B)** MC degranulation rate. **(C)** Protein expression of Tryptase and GAPDH determined by Western blot. **(D)** Tryptase protein expression. **(E)** IL-6 mRNA expression. **(F)** TNF-α mRNA expression. **(G)** IL-1β mRNA expression. *n* = 6 in each group. All data are the mean ± SD. ***p < 0.001 *vs.* the Sham group. ^*##*^ *p* < 0.01, ^*###*^ *p* < 0.001 *vs.* the I/R group. ^&&&^ *p* < 0.001 *vs.* the C48/80+I/R group.

Tryptase is the unique mediator of MC degranulation. To further explore the protective mechanism of CLE in MIRI, a series of indicators of mast cell degranulation were determined. As shown in Figure 3A, MCs in the I/R group were degranulated in contrast to the intact granules of MCs in the sham group. C48/80, a mast cell degranulator (Ohta et al., 2017), caused significantly elevated degranulation with I/R damage, as the MCs degranulated to form diffuse granules and vacuolar cells in the C48/80 + I/R group. CLE pretreatment suppressed the degranulation of MCs and the formation of diffuse granules and vacuolar cells in the CLE + I/R and CLE + C48/80 + I/R groups. Moreover, myocardial I/R injury promoted MC degranulation (Figure 3B) and tryptase protein expression (Figures 3C,D) compared with the sham group. MC degranulation and tryptase expression were obviously elevated after using C48/80, whereas CLE preconditioning partially reversed these effects. Activation of MCs generates secondary mediators such as IL-6, TNF-α, and IL-1β (Singh and Saini, 2003). As shown in Figures 3E–G, mRNA levels of IL-6, TNF-α, and IL-1β were upregulated in the I/R group compared to the sham group and C48/80 induced further upregulated mRNA levels than those in the I/R group, while the expression of these inflammatory factors was downregulated by CLE pretreatment in the CLE + I/R group and the CLE + C48/80 + I/R group compared with the I/R group and the C48/80+I/R group.

### Clemastine Fumarate Pretreatment Protected Cardiac Function by Inhibiting Mast Cell Degranulation in MIRI

**FIGURE 4:**
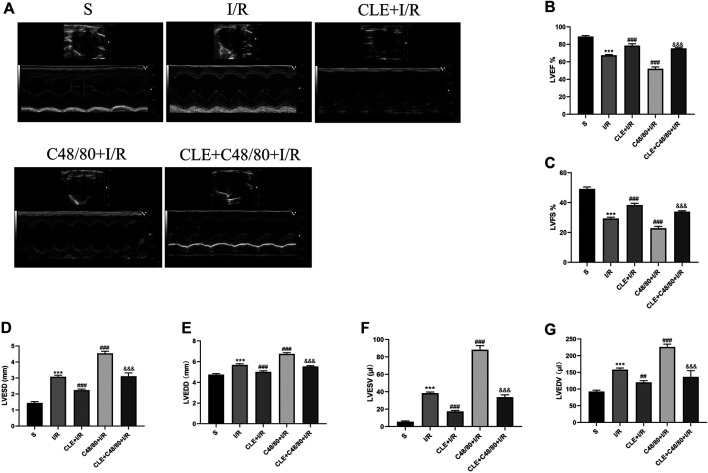
CLE pretreatment mitigated cardiac dysfunction by inhibiting MCs degranulation on MIRI. **(A)** Cardiac function detected by M-mode echocardiography. The vertical yellow lines indicate the diameter of the left ventricle at the end of systole or diastole stage. **(B)** Left ventricular ejection fraction (LVEF). **(C)** Left ventricular fraction shortening (LVFS). **(D)** Left ventricular end-systolic diameter (LVESD). **(E)** Left ventricular end-diastolic diameter (LVEDD). **(F)** Left ventricular end-systolic volume (LVESV). **(G)** Left ventricular end-diastolic volume (LVEDV). *n* = 3 in each group. All data are the mean ± SD. ***p < 0.001 *vs.* the Sham group. ^##^ *p* < 0.01, ^###^ *p* < 0.001 *vs.* the I/R group. ^&&&^ *p* < 0.001 *vs.* the C48/80+I/R group.

To investigate whether CLE pretreatment could protect cardiac function by inhibiting MC degranulation in MIRI, we selected the left ventricular systolic function indicators LVEF and LVFS. As shown in Figures 4A–C, compared with the sham group, the I/R group had reduced LVEF and LVFS and enhanced LVESD, LVEDD, LVESV, and LVEDV (Figures 4D–G). LVEF and LVFS were significantly decreased after using C48/80, and LVESD, LVEDD, LVESV, and LVEDV were significantly enhanced. However, CLE pretreatment reversed these effects.

### Clemastine Fumarate Pretreatment Alleviated Infract Size, Pathological Injury, Arrhythmias, and Myocardial Enzymes by Inhibiting Mast Cell Degranulation in MIRI

**FIGURE 5:**
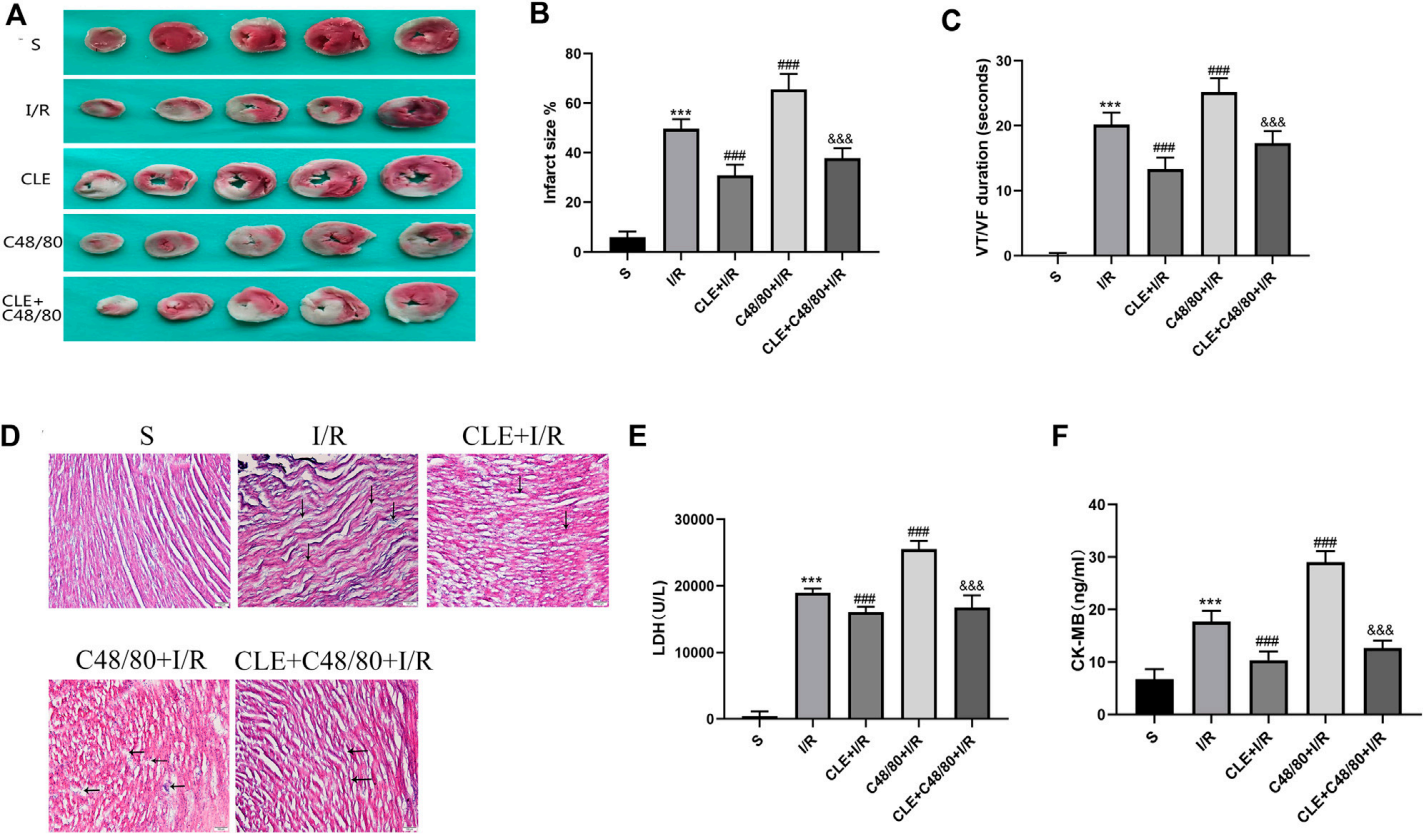
CLE pretreatment alleviated infarct size, pathological injury, arrhythmia, and myocardial enzymes by inhibiting MCs degranulation on MIRI. **(A)** Myocardial infarction area detected by TTC staining. **(B)** Infarct size%. **(C)** VT /VF duration detected by LabChart. **(D)** Cardiac pathological injury detected by HE staining (×100). The black arrows represent the obvious changes in myocardial tissue. **(E,F)** The release of LDH and CK-MB detected by ELISA. *n* = 3–6 in each group. All data are the mean ± SD. ***p < 0.001 *vs.* the Sham group. ^###^ *p* < 0.001 *vs.* the I/R group. ^&&&^ *p* < 0.001 *vs.* the C48/80 + I/R group.

To further evaluate the cardioprotective effects of CLE pretreatment by inhibiting MC degranulation in MIRI. A series of myocardial injury indicators were determined. I/R injury caused myocardial infarction, and IS was significantly increased in the C48/80+I/R group compared with the I/R group (Figures 5A,B). Both effects were attenuated by CLE pretreatment. Similarly, as shown in Figure 5D, I/R injury led to slight edema, wavy myofibers, and a small amount of inflammatory cell infiltration. Notably, C48/80 induced severe myocardial structural disorders, faulty myofibers, and an influx of inflammatory cell infiltration, but these effects were alleviated by pretreatment with CLE. C48/80 also led to prolonged ventricular tachycardia (VT) and ventricular fibrillation (VF) duration compared with those in the I/R group (Figure 5C). However, CLE pretreatment shortened VT/VF duration. Furthermore, the LDH and CK-MB levels in the I/R group were elevated compared with those in the sham group, and C48/80 significantly enhanced the level compared with that in the I/R group (Figures 5E,F). Both effects were alleviated by CLE pretreatment.

### Clemastine Fumarate Inhibited Mast Cell Degranulation *In Vitro*

**FIGURE 6:**
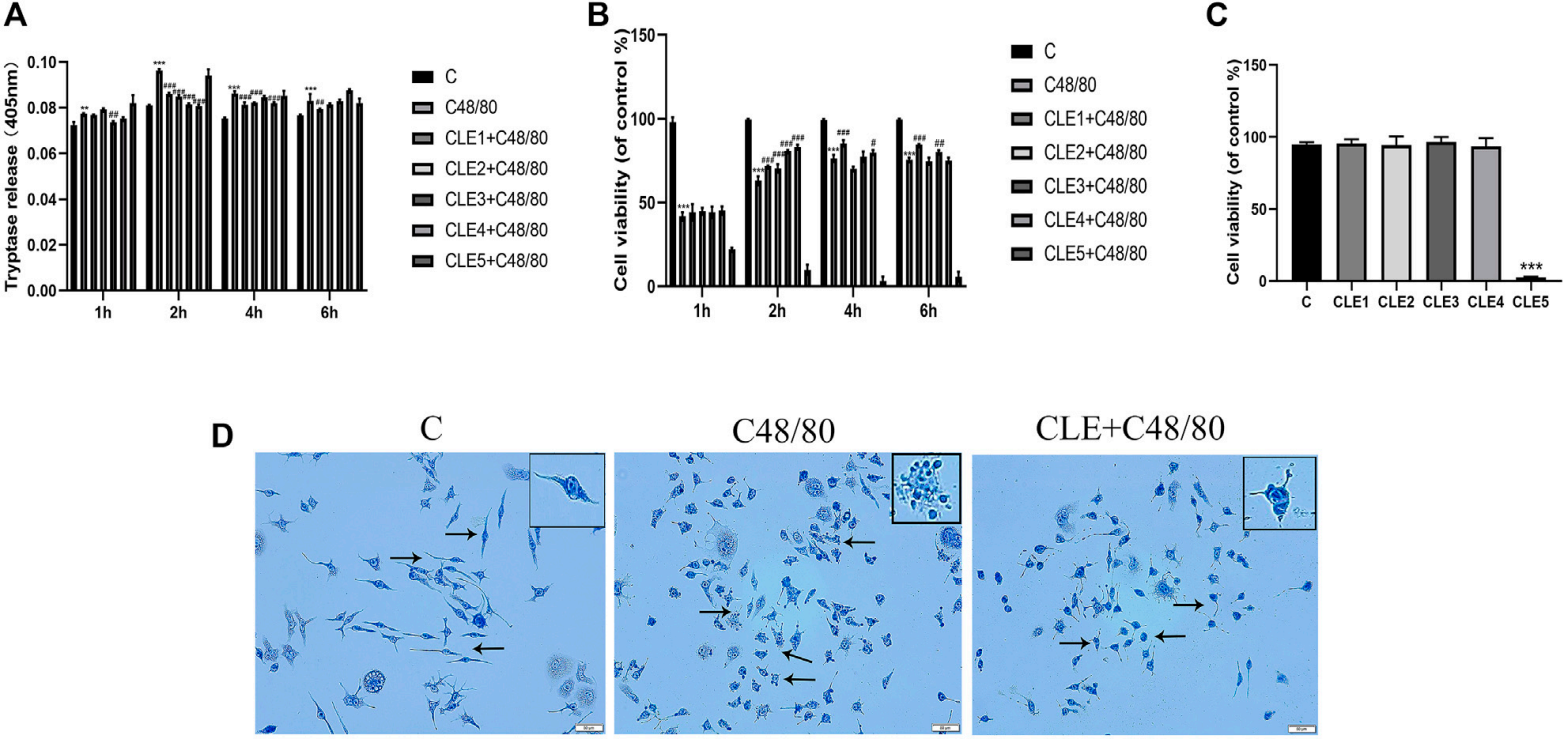
CLE pretreatment reduced MCs degranulation *in vitro*. **(A,B)** CLE interventions on degranulation of RBL-2H3 cells quantified by tryptase and CCK8 assay. **(C)** RBL-2H3 cells were pretreated with these five concentrations for 2 h and the effect of CLE on RBL-2H3 cells viability. **(D)** The effect of CLE on RBL-2H3 cells degranulation detected by TB staining (×200). The black arrows represent the typical morphology of MCs in different states. *n* = 3 independent experiments. All data are the mean ± SD. **p < 0.01 , ***p < 0.001 *vs.* the Control group; ^#^ *p* < 0.05, ^##^ *p* < 0.01, ^###^ *p* < 0.001 *vs.* the C48/80 group.

To further confirm the role of CLE in MC activation, we examined the effects of different concentrations of CLE in different periods on C48/80-induced tryptase release and cell viability. C48/80 caused a pronounced increase in tryptase release (Figure 6A) and a decrease in cell viability (Figure 6B) compared with that of the control group, which was attenuated by pretreatment with CLE at 2, 4, and 6 h. Pretreatment of RBL-2H3 cells with CLE for 2 h reduced the release of tryptase and increased cell viability, except for CLE5 (10 μg/ml), indicating that CLE pretreatment for 2 h is the optimal time. Among all the concentrations tested at 2 h, CLE4 (1 μg/ml) was suitable. As shown in Figure 6C, the other concentrations did not affect MC viability, except for CLE5. Therefore, CLE pretreatment for 2 h at a concentration of 1 μg/ml was selected for the following experiments on RBL-2H3 cells. Then, toluidine blue staining (Figure 6D) was used to evaluate the effect of CLE4 on MC degranulation. Most MCs in the control group were full of metachromatic granules. Exposure to C48/80 resulted in the release of degranulation on the surface of MCs. CLE inhibited the degranulation of MCs.

### Clemastine Fumarate Reversed Cardiomyocyte Apoptosis Caused by Mast Cell Degranulation *In Vitro*

**FIGURE 7:**
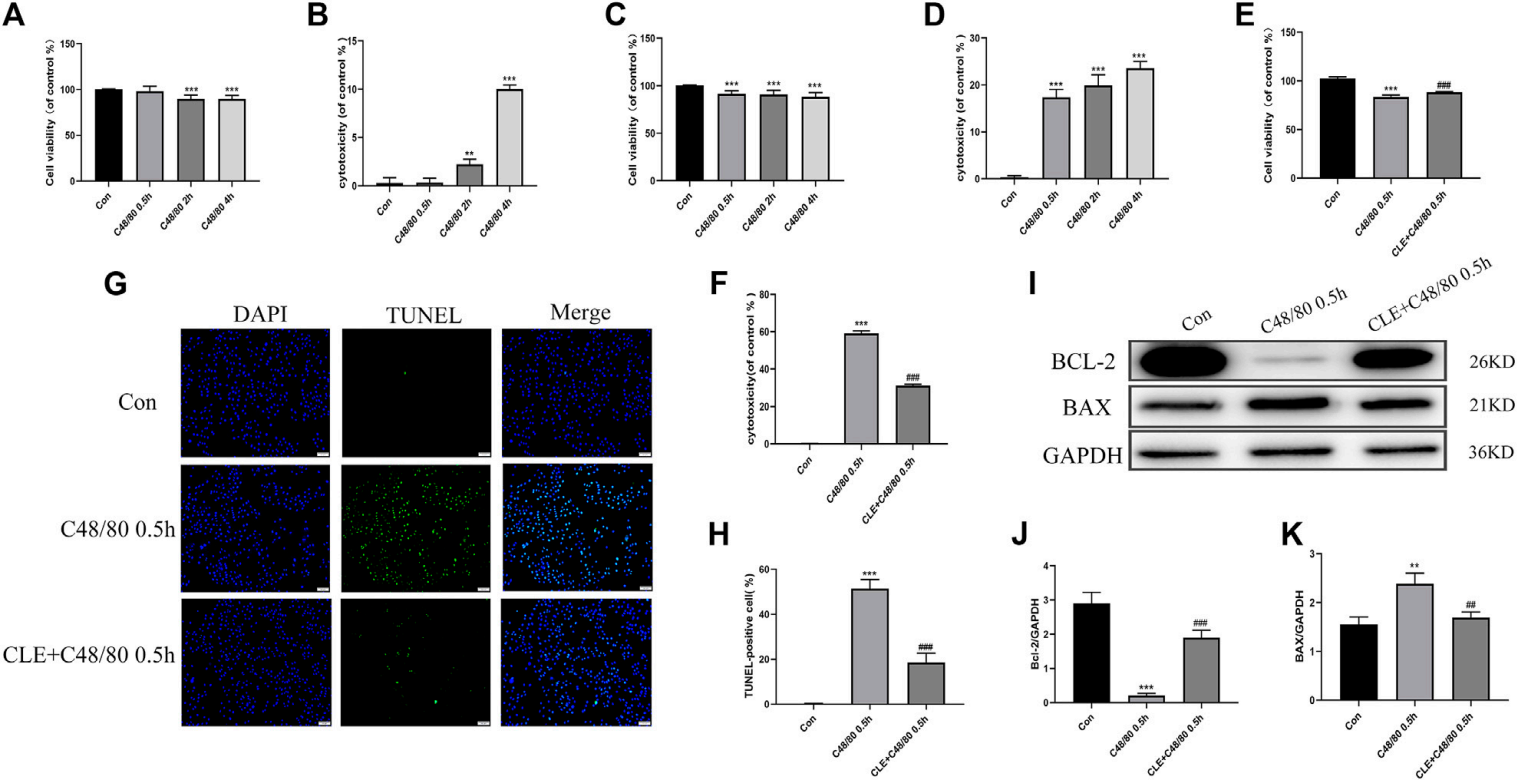
CLE pretreatment reversed the apoptosis of H9C2 cells caused by MCGs. **(A,B)** Effect of C48/80 on H9C2 survival measured by CCK-8 and LDH assay. **(C,D)** Effect of MCGs on H9C2 survival measured by CCK-8 and LDH assay. **(E,F)** Effect of CLE pretreatment with RBL on co-cultured H9C2 survival measured by CCK-8 and LDH assay. **(G)** Apoptosis of H9C2 cell measured by TUNEL staining (×100). **(H)** Apoptosis rate of H9C2 cell. **(I)** Protein expression of Bcl-2, BAX, and GAPDH determined by Western blot. **(J)** Bcl-2 protein expression. **(K)** BAX protein expression. *n* = 3–6 independent experiments. All data are the mean ± SD. **p < 0.01, ***p < 0.01 *vs.* the Control group; ^##^ *p* < 0.01, ^###^ *p* < 0.001 *vs.* the C48/80 0.5 h group.

Myocardial cell apoptosis is an essential process in the pathogenesis of myocardial I/R injury (Li et al., 2018)**.** We used RBL-2H3 cells co-cultured with H9C2 cells to investigate the effect of CLE pretreatment on cardiomyocyte apoptosis. To examine the effect of C48/80 on H9C2 cell survival, H9C2 cells were cultured in the presence of C48/80 for 0.5, 2, and 4 h. As shown in Figures 7A,B, exposure to 0.5 h had no effect on H9C2 viability and cytotoxicity, but the cell viability decreased, and cytotoxicity increased after exposure for 2 h or 4 h. When H9C2 cells were exposed to MCGs for 0.5 h, their cell viability decreased and cytotoxicity increased (Figures 7C,D). The same was true at 2 and 4 h of exposure. MC degranulation caused damage to cardiomyocytes. When we pretreated RBL cells with CLE for 2 h and stimulated them with C48/80 for 0.5 h, the viability of H9C2 cells in the CLE + C48/80 0.5 h group was obviously elevated, and the cytotoxicity was mitigated compared with that in the C48/80 0.5 h group (Figures 7E,F). Similarly, MCGs significantly enhanced H9C2 cell apoptosis compared with the Con group, while RBL cell pretreatment with CLE significantly reduced H9C2 cell apoptosis (Figures 7G,H). Moreover, as shown in Figures 7I–K, MCG injury resulted in increased BAX expression and decreased BCl-2 expression compared with the Con group. However, CLE pretreatment with RBL cells reversed these protein levels.

### Clemastine Fumarate Inhibited Degranulation of MCs by Inhibiting H1R and PKCδ

**FIGURE 8:**
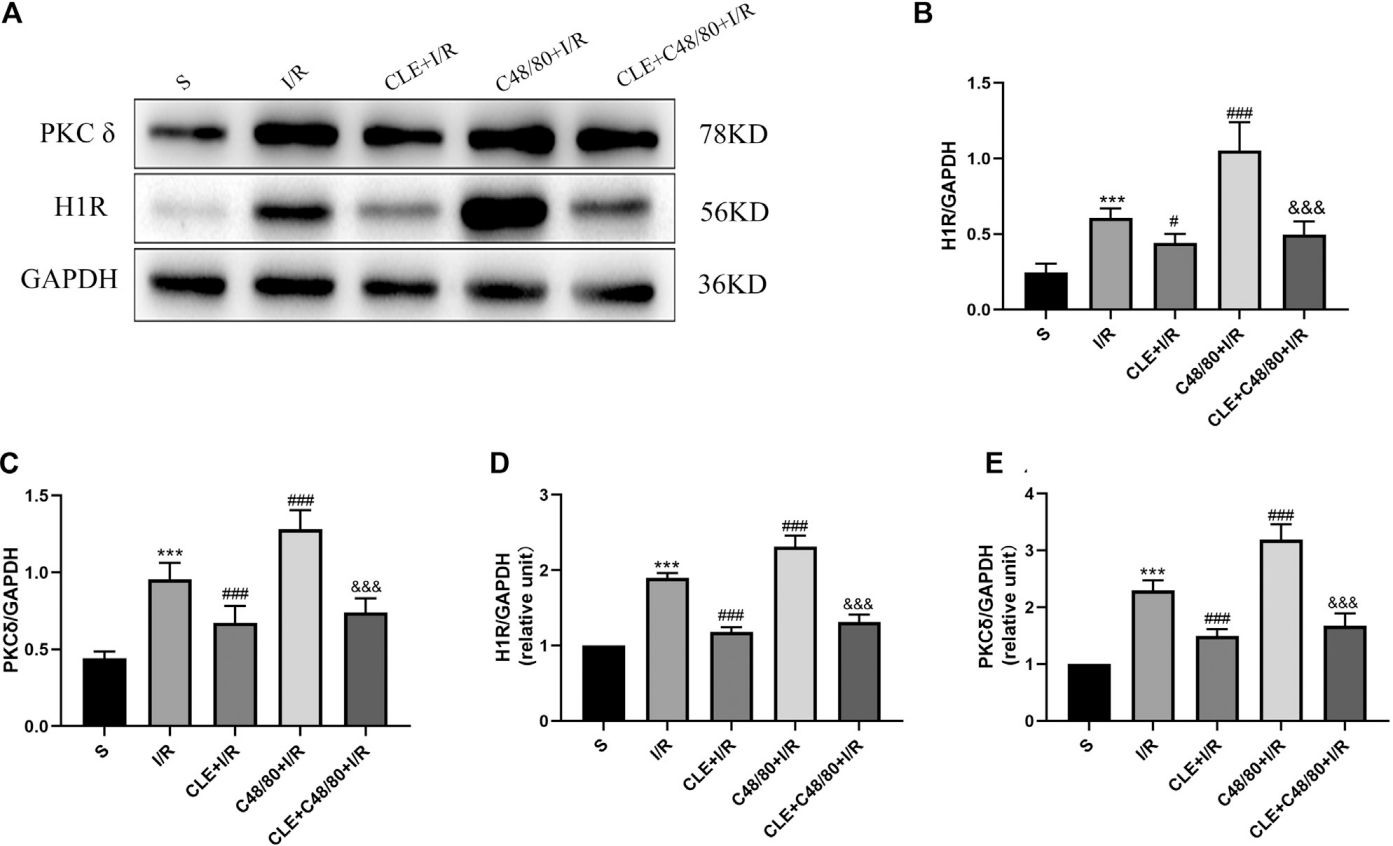
The mechanism of CLE on MC degranulation related to H1R and PKCδ. **(A)** Protein expression of H1R, PKCδ, and GAPDH determined by Western blot. **(B)** H1R protein expression. **(C)** PKCδ protein expression. **(D)** H1R mRNA expression. **(E)** PKCδ mRNA expression. *n* = 6 in each group. All data are the mean ± SD. ***p < 0.001 *vs*. the Sham group. ^#^ *p* < 0.05, ^###^ *p* < 0.001 *vs*. the I/R group. ^&&&^ *p* < 0.001 *vs*. the C48/80+I/R group.

To explore the regulatory mechanism of CLE in inhibiting the degranulation of MCs, we tested the protein expression and mRNA levels of histamine H1 receptor (H1R) and delta protein kinase C (PKCδ) in cardiac tissues. As shown in Figures 8A–E, myocardial I/R elevated the mRNA and protein expression of H1R and PKCδ compared with those in the sham group, and CLE was effectively alleviated. Moreover, the mRNA and protein expression of H1R and PKCδ was further increased by C48/80 during I/R, which was blocked by using CLE in the CLE + C48/80 + I/R group.

## Discussion

In this study, we observed that CLE pretreatment attenuated I/R-induced cardiac dysfunction and alleviated I/R-induced damage by improving infarct size, pathological injury, arrhythmias, and myocardial enzyme levels. The underlying mechanism of CLE protection was shown to be involved in inhibiting MC degranulation. Further *in vitro* results revealed that inhibition of MCGs reduced the apoptosis of cardiomyocytes. Moreover, the mechanism by which CLE inhibits the degranulation of MCs was related to the downregulation of H1R and PKCδ expression. This study could help us understand the pharmacology and mechanism of CLE pretreatment in MIRI.

Although early reperfusion therapy is critical for myocardial ischemia, blood flow recovery exacerbates damage to ischemic tissue, which is called reperfusion injury, and causes further myocardial damage (Ye et al., 2020). Over the past decades, there has been no consensus on treating myocardial I/R injury, and many cardiac protection strategies have been tested clinically. However, the results were unsatisfactory (Hausenloy and Yellon, 2016). Therefore, it is necessary to further explore the mechanism of I/R and develop a novel treatment for MIRI. As a second-generation histamine H1 receptor blocker, CLE is used to treat allergic diseases and has a protective effect on cardiomyocytes in MIRI *in vitro* (Yuan et al., 2020). In this study, a rat model of MIRI was used to discuss the protective effect of CLE. We observed that CLE pretreatment improved cardiac function and pathological injury and reduced myocardial infarct size and arrhythmias. These results indicated that CLE pretreatment attenuated I/R injury. Moreover, we also observed an interesting phenomenon in which serum tryptase was increased in the I/R group but decreased after CLE pretreatment. Furthermore, tryptase is the unique mediator of MC degranulation. Whether CLE protects the myocardium by inhibiting mast cell degranulation is unclear.

Mast cells originate from bone marrow tissue and migrate to the target tissue through the circulatory system to complete differentiation and maturation (Collington et al., 2011). Accumulating evidence has demonstrated that I/R injury is closely related to MC activation and degranulation (He et al., 2019). Myocardial ischemia is a potent stimulus that causes the activation of MCs in the heart and the release of cytotoxic mediators such as IL-6, TNF-α, and IL-1β (Yu et al., 2018). Furthermore, preformed TNF-α from resident cardiac MCs has a negative effect on the myocardium (Squadrito et al., 1993; Frangogiannis et al., 1998). TNF-α plays a crucial role in upregulating IL-6 in permeating leukocytes and initiating the cytokine cascade in charge of myocyte ICAM-1 formation and subsequent injury caused by neutrophils (Frangogiannis et al., 1998). In addition, IL-1β released from MCs also triggers myocyte apoptosis by increasing the expression of iNOS and the development of NO (Gordon et al., 1990; Ing et al., 1999). Therefore, inflammatory factors from the degranulation of MCs also cause damage to the heart. Moreover, MC-derived renin released by I/R initiated the activation of a local renin-angiotensin system (RAS). These factors cause severe arrhythmic dysfunction, such as VT and VF, by improving excessive norepinephrine (NE) release (Aldi et al., 2015). However, ketotifen and low-dose carvedilol, commonly used as mast cell stabilizers, significantly reduced myocardial I/R injury (Jaggi et al., 2007). All these studies support that inhibiting MC degranulation would be beneficial to mitigate MIRI. H1 receptor antagonists can inhibit the release of histamine and proinflammatory cytokines from MCs and the recruitment of MC-dependent leukocytes during I/R (Singh and Saini, 2003). Consistent with these previous studies, we observed that CLE pretreatment could reduce the MC degranulation rate, the release of tryptase, and inflammatory factors during MIRI.

C48/80, as a mast cell activator, can activate and promote the degranulation of MCs. Studies have shown that the use of C48/80 during I/R further promotes MC degranulation and aggravates the original damage to ischemic tissue (Gan et al., 2015). This study used C48/80 to further accelerate MC degranulation in the myocardial I/R state. The results revealed that C48/80 combined with I/R significantly impaired cardiac function, enlarged infarct size, aggravated pathological damage, induced arrhythmia, and increased myocardial enzymes. However, pretreatment with CLE reversed these changes, indicating that the protective effect of CLE is achieved by inhibiting MCs. In *in vitro* experiments, C48/80 was used to stimulate RBL cells to simulate the degranulation of MCs caused by I/R injury, while pretreatment with CLE decreased tryptase release and increased cell viability, resulting in alleviation of MC degranulation. Therefore, it was further verified that CLE improves myocardial I/R injury by inhibiting MC degranulation.

Apoptosis, also known as programmed cell death, is another crucial process in the pathogenesis of MIRI. Increased apoptosis may lead to tissue damage and dysfunction under I/R pathophysiologic conditions (Li et al., 2018). Resident MC degranulation is an early event in I/R injury. Acute stress activates MCs usually existing in the myocardium in a complete form; at the same time, enzymes (such as chymase and tryptase) and proinflammatory mediators (such as TNF-α, IL-6, and IL-1β) are released into the cardiac interstitium (Zheng et al., 2014; Varricchi et al., 2019). When cardiomyocytes are exposed to MCGs, cardiomyocyte apoptosis might occur. NR4A1, as an orphan nuclear receptor, causes rapid structural changes and mitochondrial damage in myocytes following chymase entry into cardiomyocytes (Zheng et al., 2014). Calcium-independent phospholipase A2 (iPLA2) is activated by tryptase stimulation in ventricular myocytes, leading to the origination or transmission of inflammation. Thus, MC-releasing tryptase influences cardiac myocytes (Sharma and Mchowat, 2011). In addition, TNF-α and IL-1β released by MCs can also cause cardiomyocyte damage. These results indicate that MC degranulation may cause cardiomyocyte damage, consistent with our conditional training results. MCGs decreased H9C2 cell viability and the antiapoptotic protein Bcl-2. Moreover, it increased H9C2 cell cytotoxicity, the cell apoptosis rate, and the proapoptotic protein BAX. However, RBL cell pretreatment with CLE reversed these effects. These results revealed that CLE could mitigate the apoptosis of cardiomyocytes caused by MC degranulation.

In our present study, as a histamine H1 receptor blocker, CLE had a protective effect on cardiac I/R and inhibited the degranulation of MCs. Similarly, our research result is to consistent with the results of previous studies on blockers of H1 receptors (Valen et al., 1994; Singh and Saini, 2003). However, the mechanism by which H1 receptor blockers and CLE inhibit MC degranulation is not clear. We hypothesize that it works by inhibiting the H1R-PKCδ pathway. During the cardiac reperfusion phase, ROS are increased and enter the cell, or G-protein-coupled receptors (GPCRs) expressed on the MC surface are activated by complement molecules C3a and C5a, causing IP3 and DAG fabrication. DAG is mediated by phospholipase C issues in calcium leakage out of the endoplasmic reticulum and stimulation of protein kinase C (PKC), leading to degranulation of the MC (Krystel-Whittemore et al., 2015). H1R is located in many tissues and cells, including MCs (Thangam et al., 2018). H1R also belongs to the superfamily of GPCRs. The inositol phospholipid signaling pathways were activated by H1R-coupled Gq/11 activation, leading to the formation of IP3 and DAG, resulting in intracellular calcium improvement and PKC stimulation, and H1R-PKC was shown to be involved in activating MCs (Leurs et al., 2002). PKC expressed downstream of H1R contains a family of 10 related serine/threonine kinases (Zhao et al., 2016; Parra-Abarca et al., 2019). PKCε and PKCδ are the two most studied subtypes related to MIRI. They have opposing roles in regulating myocardial damage induced by I/R. PKCε is activated to save the heart from ischemia-induced damage, whereas PKCδ inhibition during reperfusion saves the heart from reperfusion-induced injury (Budas et al., 2007). Recent research has shown that local RAS activation, Ang II generation, and NE release are inhibited by activating MC GPCRs and the consequent stimulation of the PKCε/ALDH2 pathway, leading to ventricular arrhythmias in myocardial ischemia (Marino and Levi, 2018). Due to the opposite effects of PKCε and PKCδ in MIRI, PKCε is related to inhibiting MC degranulation, and PKCδ may be related to promoting MC degranulation. Our results showed that the mRNA and protein expression of H1R and PKCδ increased in I/R. C48/80 intensified the increase in the mRNA and protein expression of H1R and PKCδ. In contrast, CLE could reverse the rise of both. Therefore, the H1R-PKCδ signaling pathway may be involved in MC degranulation during I/R, and whether changes in PKCε exist in MCs in MIRI progression with CLE pretreatment will be researched in future studies.

Our research has some limitations. First, animal heart I/R injury cannot completely simulate the patient’s heart I/R injury. The mechanism by which CLE protects against MIRI may not only inhibit MC degranulation but also involve other mechanisms. Second, the best assessment of infarct size (IS) is TTC and Evans blue double staining and evaluates ventricular % area at risk (AAR). TTC staining alone may have limitations on the level of LAD ligation, which causes our results on IS to be meaningful only at the TTC level. Third, we did not rule out other types of PKC downstream of H1R and did not further explore the H1R and PKCδ pathways in MIRI. We will continue to determine the cardioprotective effects of CLE on MC degranulation *in vivo* and *in vitro*, particularly the exact involvement of the H1R-PKCδ signaling pathway.

**FIGURE 9:**
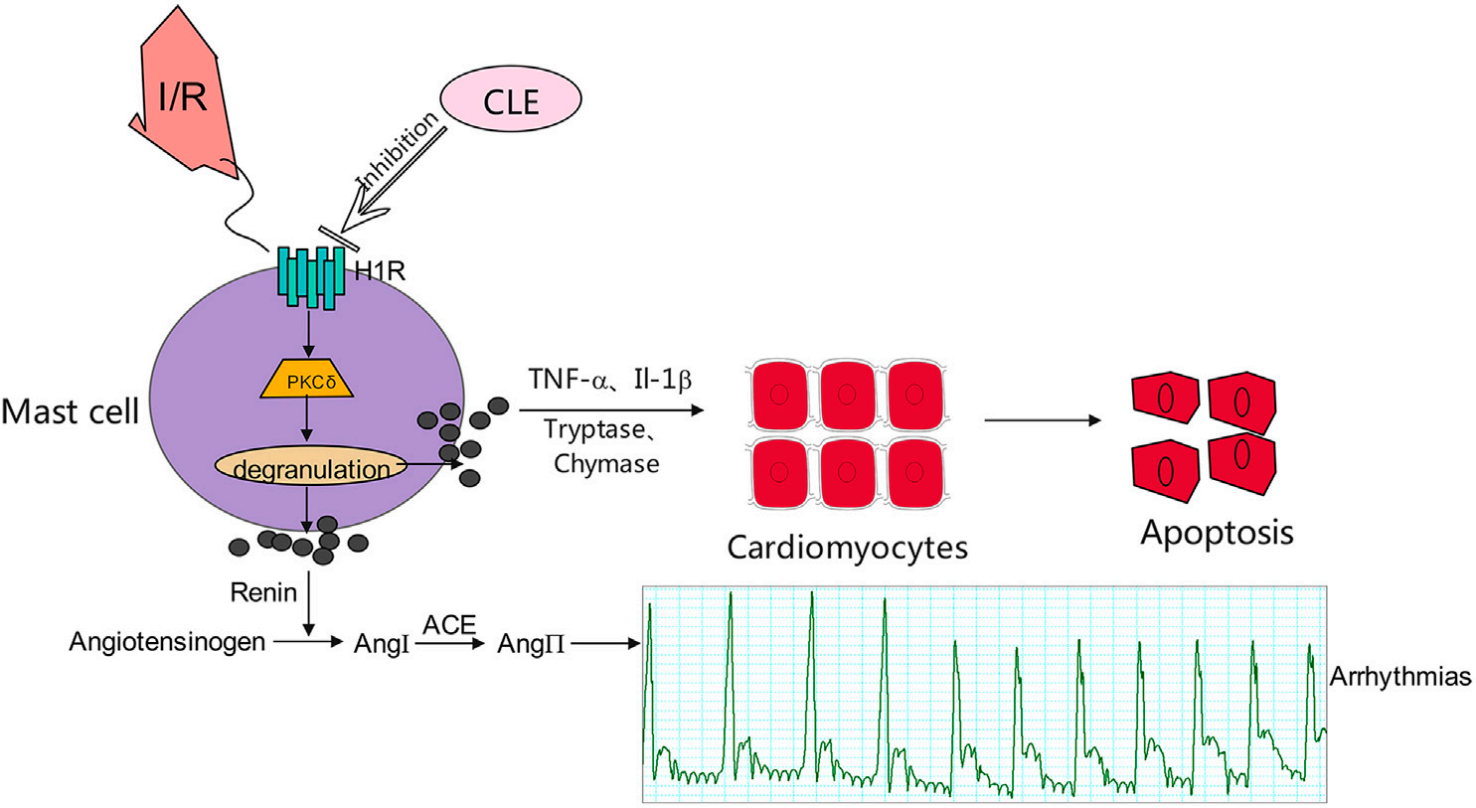
Proposed mechanisms for the effect of CLE in inhibiting degranulation of MCs through the H1R- PKCδ signaling pathway.

Overall, we speculate that CLE prevented MIRI by inhibiting MC degranulation in a rat MIRI model. CLE could mitigate the apoptosis of cardiomyocytes caused by MC degranulation. The mechanism of action of CLE may be involved in the H1R and PKCδ signaling pathways (Figure 9). This study is the first to provide evidence that the protective effect of CLE is associated with preventing MCs from degranulating in MIRI and to show a possible mechanism of CLE acting on MCs. This novel H1R-PKCδ signaling pathway for the action of CLE could be exploited for more extensive pharmacological action beyond the antiallergic effect.

## Data Availability

The original contributions presented in the study are included in the article/Supplementary Material, further inquiries can be directed to the corresponding author.
