# Supplementary material for: Clemastine Fumarate Attenuates Myocardial Ischemia Reperfusion Injury Through Inhibition of Mast Cell Degranulation
Source: Front Pharmacol. 2021 Aug 27;12:704852. doi: 10.3389/fphar.2021.704852 (PMC8430029; doi:10.3389/fphar.2021.704852)
Supplement: Supplementary file 1 [file DataSheet1.ZIP › supplementary/Data Analysis/FIgure 8.pdf]

Oneway

Descriptives

		N	Mean	Std. Deviation	Std. Error	95% Confidence Interval for Mean Lower Bound
FIG.8B	S	6	.2467	.05820	.02376	.1856
	I/R	6	.6067	.06408	.02616	.5394
	CLE+I/R	6	.4433	.05888	.02404	.3815
	C48/80+I/R	6	1.0533	.18758	.07658	.8565
	CLE+C48/80+I/R	6	.4983	.08542	.03487	.4087
	Total	30	.5697	.28955	.05286	.4615
FIG.8C	S	6	.3783	.07885	.03219	.2956
	I/R	6	1.0117	.08060	.03291	.9271
	CLE+I/R	6	.7150	.09311	.03801	.6173
	C48/80+I/R	6	1.4800	.18111	.07394	1.2899
	CLE+C48/80+I/R	6	.6633	.14473	.05909	.5114
	Total	30	.8497	.39685	.07245	.7015
FIG.8D	S	6	1.00000	.000000	.000000	1.00000
	I/R	6	1.89633	.067878	.027711	1.82510
	CLE+I/R	6	1.18400	.060936	.024877	1.12005
	C48/80+I/R	6	2.31040	.151602	.061891	2.15130
	CLE+C48/80+I/R	6	1.31155	.100290	.040943	1.20630
	Total	30	1.54046	.503536	.091933	1.35243
FIG.8E	S	6	1.0000	.00000	.00000	1.0000
	I/R	6	2.2960	.18045	.07367	2.1066
	CLE+I/R	6	1.4942	.12169	.04968	1.3665
	C48/80+I/R	6	3.1903	.26913	.10987	2.9079
	CLE+C48/80+I/R	6	1.6757	.21949	.08961	1.4453
	Total	30	1.9312	.78546	.14340	1.6379

Descriptives

		95% Confidence Interval for Mean Upper Bound	Minimum	Maximum
FIG.8B	S	.3077	.16	.32
	I/R	.6739	.53	.68
	CLE+I/R	.5051	.37	.53
	C48/80+I/R	1.2502	.91	1.42
	CLE+C48/80+I/R	.5880	.37	.59

	Total	.6778	.16	1.42
FIG.8C	S	.4611	.28	.49
	I/R	1.0963	.93	1.12
	CLE+I/R	.8127	.61	.88
	C48/80+I/R	1.6701	1.25	1.67
	CLE+C48/80+I/R	.8152	.42	.81
	Total	.9979	.28	1.67
FIG.8D	S	1.00000	1.000	1.000
	I/R	1.96757	1.800	1.992
	CLE+I/R	1.24795	1.112	1.265
	C48/80+I/R	2.46950	2.136	2.582
	CLE+C48/80+I/R	1.41679	1.206	1.474
	Total	1.72848	1.000	2.582
FIG.8E	S	1.0000	1.00	1.00
	I/R	2.4854	2.05	2.58
	CLE+I/R	1.6219	1.32	1.65
	C48/80+I/R	3.4728	2.81	3.59
	CLE+C48/80+I/R	1.9060	1.41	1.99
	Total	2.2245	1.00	3.59

ANOVA

		Sum of Squares	df	Mean Square	F	Sig.
FIG.8B	Between Groups	2.164	4	.541	50.616	.000
	Within Groups	.267	25	.011		
	Total	2.431	29			
FIG.8C	Between Groups	4.191	4	1.048	69.737	.000
	Within Groups	.376	25	.015		
	Total	4.567	29			
FIG.8D	Between Groups	7.146	4	1.787	215.963	.000
	Within Groups	.207	25	.008		
	Total	7.353	29			
FIG.8E	Between Groups	17.052	4	4.263	126.890	.000
	Within Groups	.840	25	.034		
	Total	17.891	29			

Post Hoc Tests

Multiple Comparisons

Dependent Variable		(I) Groups	(J) Groups	Mean Difference (I-J)	Std. Error	Sig.	95% Confidence Interval Lower Bound	Upper Bound
FIG.8B	LSD	S	I/R	-.36000*	.05969	.000	-.4829	-.2371
			CLE+I/R	-.19667*	.05969	.003	-.3196	-.0737
			C48/80+I/R	-.80667*	.05969	.000	-.9296	-.6837
			CLE+C48/80+I/R	-.25167*	.05969	.000	-.3746	-.1287
		I/R	S	.36000*	.05969	.000	.2371	.4829
			CLE+I/R	.16333*	.05969	.011	.0404	.2863
			C48/80+I/R	-.44667*	.05969	.000	-.5696	-.3237
			CLE+C48/80+I/R	.10833	.05969	.082	-.0146	.2313
		CLE+I/R	S	.19667*	.05969	.003	.0737	.3196
			I/R	-.16333*	.05969	.011	-.2863	-.0404
			C48/80+I/R	-.61000*	.05969	.000	-.7329	-.4871
			CLE+C48/80+I/R	-.05500	.05969	.366	-.1779	.0679
		C48/80+I/R	S	.80667*	.05969	.000	.6837	.9296
			I/R	.44667*	.05969	.000	.3237	.5696
			CLE+I/R	.61000*	.05969	.000	.4871	.7329
			CLE+C48/80+I/R	.55500*	.05969	.000	.4321	.6779
		CLE+C48/80+I/R	S	.25167*	.05969	.000	.1287	.3746
			I/R	-.10833	.05969	.082	-.2313	.0146
			CLE+I/R	.05500	.05969	.366	-.0679	.1779
			C48/80+I/R	-.55500*	.05969	.000	-.6779	-.4321
FIG.8C	LSD	S	I/R	-.63333*	.07077	.000	-.7791	-.4876
			CLE+I/R	-.33667*	.07077	.000	-.4824	-.1909
			C48/80+I/R	-1.10167*	.07077	.000	-1.2474	-.9559
			CLE+C48/80+I/R	-.28500*	.07077	.000	-.4308	-.1392
		I/R	S	.63333*	.07077	.000	.4876	.7791
			CLE+I/R	.29667*	.07077	.000	.1509	.4424
			C48/80+I/R	-.46833*	.07077	.000	-.6141	-.3226
			CLE+C48/80+I/R	.34833*	.07077	.000	.2026	.4941

		CLE+I/R	S	.33667*	.07077	.000	.1909	.4824
			I/R	-.29667*	.07077	.000	-.4424	-.1509
			C48/80+I/R	-.76500*	.07077	.000	-.9108	-.6192
			CLE+C48/80+I/R	.05167	.07077	.472	-.0941	.1974
		C48/80+I/R	S	1.10167*	.07077	.000	.9559	1.2474
			I/R	.46833*	.07077	.000	.3226	.6141
			CLE+I/R	.76500*	.07077	.000	.6192	.9108
			CLE+C48/80+I/R	.81667*	.07077	.000	.6709	.9624
		CLE+C48/80+I/R	S	.28500*	.07077	.000	.1392	.4308
			I/R	-.34833*	.07077	.000	-.4941	-.2026
			CLE+I/R	-.05167	.07077	.472	-.1974	.0941
			C48/80+I/R	-.81667*	.07077	.000	-.9624	-.6709
FIG.8D	LSD	S	I/R	-.896333*	.052511	.000	-1.00448	-.78818
			CLE+I/R	-.184000*	.052511	.002	-.29215	-.07585
			C48/80+I/R	-1.310400*	.052511	.000	-1.41855	-1.20225
			CLE+C48/80+I/R	-.311547*	.052511	.000	-.41970	-.20340
		I/R	S	.896333*	.052511	.000	.78818	1.00448
			CLE+I/R	.712333*	.052511	.000	.60418	.82048
			C48/80+I/R	-.414067*	.052511	.000	-.52222	-.30592
			CLE+C48/80+I/R	.584787*	.052511	.000	.47664	.69294
		CLE+I/R	S	.184000*	.052511	.002	.07585	.29215
			I/R	-.712333*	.052511	.000	-.82048	-.60418
			C48/80+I/R	-1.126400*	.052511	.000	-1.23455	-1.01825
			CLE+C48/80+I/R	-.127547*	.052511	.023	-.23570	-.01940
		C48/80+I/R	S	1.310400*	.052511	.000	1.20225	1.41855
			I/R	.414067*	.052511	.000	.30592	.52222
			CLE+I/R	1.126400*	.052511	.000	1.01825	1.23455
			CLE+C48/80+I/R	.998853*	.052511	.000	.89070	1.10700
		CLE+C48/80+I/R	S	.311547*	.052511	.000	.20340	.41970
			I/R	-.584787*	.052511	.000	-.69294	-.47664
			CLE+I/R	.127547*	.052511	.023	.01940	.23570
			C48/80+I/R	-.998853*	.052511	.000	-1.10700	-.89070
FIG.8E	LSD	S	I/R	-1.29600*	.10582	.000	-1.5139	-1.0781
			CLE+I/R	-.49417*	.10582	.000	-.7121	-.2762
			C48/80+I/R	-2.19033*	.10582	.000	-2.4083	-1.9724

	CLE+C48/80+I/R	-.67567*	.10582	.000	-.8936	-.4577
I/R	S	1.29600*	.10582	.000	1.0781	1.5139
	CLE+I/R	.80183*	.10582	.000	.5839	1.0198
	C48/80+I/R	-.89433*	.10582	.000	-1.1123	-.6764
	CLE+C48/80+I/R	.62033*	.10582	.000	.4024	.8383
CLE+I/R	S	.49417*	.10582	.000	.2762	.7121
	I/R	-.80183*	.10582	.000	-1.0198	-.5839
	C48/80+I/R	-1.69617*	.10582	.000	-1.9141	-1.4782
	CLE+C48/80+I/R	-.18150	.10582	.099	-.3994	.0364
C48/80+I/R	S	2.19033*	.10582	.000	1.9724	2.4083
	I/R	.89433*	.10582	.000	.6764	1.1123
	CLE+I/R	1.69617*	.10582	.000	1.4782	1.9141
	CLE+C48/80+I/R	1.51467*	.10582	.000	1.2967	1.7326
CLE+C48/80+I/R	S	.67567*	.10582	.000	.4577	.8936
	I/R	-.62033*	.10582	.000	-.8383	-.4024
	CLE+I/R	.18150	.10582	.099	-.0364	.3994
	C48/80+I/R	-1.51467*	.10582	.000	-1.7326	-1.2967

*. The mean difference is significant at the 0.05 level.

Homogeneous Subsets

FIG.8B

	Groups	N	Subset for alpha = 0.05		
			1	2	3
Student-Newman-Keuls[a]	S	6	.2467		
	CLE+I/R	6		.4433	
	CLE+C48/80+I/R	6		.4983	.4983
	I/R	6			.6067
	C48/80+I/R	6			
	Sig.		1.000	.366	.082

FIG.8B

	Groups	Subset for alpha = 0.05<br>4
Student-Newman-Keuls[a]	S	
	CLE+I/R	
	CLE+C48/80+I/R	
	I/R	
	C48/80+I/R	1.0533
	Sig.	1.000

Means for groups in homogeneous subsets are displayed.

a. Uses Harmonic Mean Sample Size = 6.000.

FIG.8C

	Groups	N	Subset for alpha = 0.05<br>1	2	3
Student-Newman-Keuls[a]	S	6	.3783		
	CLE+C48/80+I/R	6		.6633	
	CLE+I/R	6		.7150	
	I/R	6			1.0117
	C48/80+I/R	6			
	Sig.		1.000	.472	1.000

FIG.8C

	Groups	Subset for alpha = 0.05<br>4
Student-Newman-Keuls[a]	S	
	CLE+C48/80+I/R	
	CLE+I/R	
	I/R	
	C48/80+I/R	1.4800
	Sig.	1.000

Means for groups in homogeneous subsets are displayed.

a. Uses Harmonic Mean Sample Size = 6.000.

FIG.8D

Groups	N	Subset for alpha = 0.05<br>1	2	3

Student-Newman-Keuls[a]	S	6	1.00000		
	CLE+I/R	6		1.18400	
	CLE+C48/80+I/R	6			1.31155
	I/R	6			
	C48/80+I/R	6			
	Sig.		1.000	1.000	1.000

FIG.8D

	Groups	Subset for alpha = 0.05 4	5
Student-Newman-Keuls[a]	S		
	CLE+I/R		
	CLE+C48/80+I/R		
	I/R	1.89633	
	C48/80+I/R		2.31040
	Sig.	1.000	1.000

Means for groups in homogeneous subsets are displayed.

a. Uses Harmonic Mean Sample Size = 6.000.

FIG.8E

	Groups	N	Subset for alpha = 0.05 1	2	3
Student-Newman-Keuls[a]	S	6	1.0000		
	CLE+I/R	6		1.4942	
	CLE+C48/80+I/R	6		1.6757	
	I/R	6			2.2960
	C48/80+I/R	6			
	Sig.		1.000	.099	1.000

FIG.8E

	Groups	Subset for alpha = 0.05 4
Student-Newman-Keuls[a]	S	
	CLE+I/R	
	CLE+C48/80+I/R	
	I/R	
	C48/80+I/R	3.1903
	Sig.	1.000

Means for groups in homogeneous subsets are displayed.

a. Uses Harmonic Mean Sample Size = 6.000.
