# Supplementary material for: Clemastine Fumarate Attenuates Myocardial Ischemia Reperfusion Injury Through Inhibition of Mast Cell Degranulation
Source: Front Pharmacol. 2021 Aug 27;12:704852. doi: 10.3389/fphar.2021.704852 (PMC8430029; doi:10.3389/fphar.2021.704852)
Supplement: Supplementary file 1 [file DataSheet1.ZIP › supplementary/Data Analysis/Figure 2.pdf]

# Oneway

**Descriptives**

| | | N | Mean | Std. Deviation | Std. Error | 95% Confidence Interval for Mean Lower Bound |
|---|---|---|---|---|---|---|
| FIG.2B | S | 3 | .88975 | .022619 | .013059 | .83356 |
| | I/R | 3 | .68475 | .015459 | .008925 | .64634 |
| | CLE+I/R | 3 | .77412 | .018816 | .010864 | .72737 |
| | Total | 9 | .78287 | .090548 | .030183 | .71327 |
| FIG.2C | S | 3 | .49842 | .025903 | .014955 | .43408 |
| | I/R | 3 | .29395 | .009859 | .005692 | .26946 |
| | CLE+I/R | 3 | .37694 | .022831 | .013182 | .32022 |
| | Total | 9 | .38977 | .090851 | .030284 | .31994 |
| FIG.2D | S | 6 | .1667 | .40825 | .16667 | -.2618 |
| | I/R | 6 | 5.5000 | .54772 | .22361 | 4.9252 |
| | CLE+I/R | 6 | 3.3333 | .81650 | .33333 | 2.4765 |
| | Total | 18 | 3.0000 | 2.32632 | .54832 | 1.8431 |
| FIG.2E | S | 3 | .0000 | .00000 | .00000 | .0000 |
| | I/R | 3 | .8583 | .05204 | .03005 | .7291 |
| | CLE+I/R | 3 | .8143 | .06258 | .03613 | .6589 |
| | Total | 9 | .5576 | .42057 | .14019 | .2343 |
| FIG.2G | S | 3 | .05047 | .007441 | .004296 | .03198 |
| | I/R | 3 | .50333 | .020817 | .012019 | .45162 |
| | CLE+I/R | 3 | .33333 | .041633 | .024037 | .22991 |
| | Total | 9 | .29571 | .199514 | .066505 | .14235 |
| FIG.2I | S | 6 | 1.1092 | .02392 | .00977 | 1.0841 |
| | I/R | 6 | 1.3687 | .03204 | .01308 | 1.3350 |
| | CLE+I/R | 6 | 1.2538 | .02570 | .01049 | 1.2269 |
| | Total | 18 | 1.2439 | .11225 | .02646 | 1.1881 |

**Descriptives**

| | | 95% Confidence Interval for Mean Upper Bound | Minimum | Maximum |
|---|---|---|---|---|
| FIG.2B | S | .94594 | .867 | .913 |
| | I/R | .72315 | .667 | .694 |
| | CLE+I/R | .82086 | .759 | .795 |

| | | | | |
|---|---|---|---|---|
| | Total | .85247 | .667 | .913 |
| FIG.2C | S | .56277 | .472 | .524 |
| | I/R | .31844 | .287 | .305 |
| | CLE+I/R | .43366 | .352 | .396 |
| | Total | .45961 | .287 | .524 |
| FIG.2D | S | .5951 | .00 | 1.00 |
| | I/R | 6.0748 | 5.00 | 6.00 |
| | CLE+I/R | 4.1902 | 2.00 | 4.00 |
| | Total | 4.1569 | .00 | 6.00 |
| FIG.2E | S | .0000 | .00 | .00 |
| | I/R | .9876 | .80 | .90 |
| | CLE+I/R | .9698 | .75 | .88 |
| | Total | .8808 | .00 | .90 |
| FIG.2G | S | .06895 | .044 | .059 |
| | I/R | .55504 | .480 | .520 |
| | CLE+I/R | .43676 | .300 | .380 |
| | Total | .44907 | .044 | .520 |
| FIG.2I | S | 1.1343 | 1.06 | 1.13 |
| | I/R | 1.4023 | 1.31 | 1.39 |
| | CLE+I/R | 1.2808 | 1.21 | 1.29 |
| | Total | 1.2997 | 1.06 | 1.39 |

**ANOVA**

| | | Sum of Squares | df | Mean Square | F | Sig. |
|---|---|---|---|---|---|---|
| FIG.2B | Between Groups | .063 | 2 | .032 | 86.065 | .000 |
| | Within Groups | .002 | 6 | .000 | | |
| | Total | .066 | 8 | | | |
| FIG.2C | Between Groups | .063 | 2 | .032 | 73.816 | .000 |
| | Within Groups | .003 | 6 | .000 | | |
| | Total | .066 | 8 | | | |
| FIG.2D | Between Groups | 86.333 | 2 | 43.167 | 114.265 | .000 |
| | Within Groups | 5.667 | 15 | .378 | | |
| | Total | 92.000 | 17 | | | |
| FIG.2E | Between Groups | 1.402 | 2 | .701 | 317.407 | .000 |
| | Within Groups | .013 | 6 | .002 | | |
| | Total | 1.415 | 8 | | | |
| FIG.2G | Between Groups | .314 | 2 | .157 | 211.969 | .000 |

| | | | | | | |
|---|---|---|---|---|---|---|
| | Within Groups | .004 | 6 | .001 | | |
| | Total | .318 | 8 | | | |
| FIG.2I | Between Groups | .203 | 2 | .101 | 134.735 | .000 |
| | Within Groups | .011 | 15 | .001 | | |
| | Total | .214 | 17 | | | |

# Post Hoc Tests

**Multiple Comparisons**

| Dependent Variable | | (I) Groups | (J) Groups | Mean Difference (I-J) | Std. Error | Sig. | 95% Confidence Interval Lower Bound | 95% Confidence Interval Upper Bound |
|---|---|---|---|---|---|---|---|---|
| FIG.2B | LSD | S | I/R | .205000* | .015668 | .000 | .16666 | .24334 |
| | | | CLE+I/R | .115630* | .015668 | .000 | .07729 | .15397 |
| | | I/R | S | -.205000* | .015668 | .000 | -.24334 | -.16666 |
| | | | CLE+I/R | -.089370* | .015668 | .001 | -.12771 | -.05103 |
| | | CLE+I/R | S | -.115630* | .015668 | .000 | -.15397 | -.07729 |
| | | | I/R | .089370* | .015668 | .001 | .05103 | .12771 |
| FIG.2C | LSD | S | I/R | .204470* | .016927 | .000 | .16305 | .24589 |
| | | | CLE+I/R | .121483* | .016927 | .000 | .08006 | .16290 |
| | | I/R | S | -.204470* | .016927 | .000 | -.24589 | -.16305 |
| | | | CLE+I/R | -.082987* | .016927 | .003 | -.12441 | -.04157 |
| | | CLE+I/R | S | -.121483* | .016927 | .000 | -.16290 | -.08006 |
| | | | I/R | .082987* | .016927 | .003 | .04157 | .12441 |
| FIG.2D | LSD | S | I/R | -5.33333* | .35486 | .000 | -6.0897 | -4.5770 |
| | | | CLE+I/R | -3.16667* | .35486 | .000 | -3.9230 | -2.4103 |
| | | I/R | S | 5.33333* | .35486 | .000 | 4.5770 | 6.0897 |
| | | | CLE+I/R | 2.16667* | .35486 | .000 | 1.4103 | 2.9230 |
| | | CLE+I/R | S | 3.16667* | .35486 | .000 | 2.4103 | 3.9230 |
| | | | I/R | -2.16667* | .35486 | .000 | -2.9230 | -1.4103 |
| FIG.2E | LSD | S | I/R | -.85833* | .03837 | .000 | -.9522 | -.7644 |
| | | | CLE+I/R | -.81433* | .03837 | .000 | -.9082 | -.7204 |
| | | I/R | S | .85833* | .03837 | .000 | .7644 | .9522 |
| | | | CLE+I/R | .04400 | .03837 | .295 | -.0499 | .1379 |
| | | CLE+I/R | S | .81433* | .03837 | .000 | .7204 | .9082 |
| | | | I/R | -.04400 | .03837 | .295 | -.1379 | .0499 |
| FIG.2G | LSD | S | I/R | -.452867* | .022221 | .000 | -.50724 | -.39849 |

| | | | | | | | | |
|---|---|---|---|---|---|---|---|---|
| | | | CLE+I/R | -.282867* | .022221 | .000 | -.33724 | -.22849 |
| | | I/R | S | .452867* | .022221 | .000 | .39849 | .50724 |
| | | | CLE+I/R | .170000* | .022221 | .000 | .11563 | .22437 |
| | | CLE+I/R | S | .282867* | .022221 | .000 | .22849 | .33724 |
| | | | I/R | -.170000* | .022221 | .000 | -.22437 | -.11563 |
| FIG.2I | LSD | S | I/R | -.25950* | .01584 | .000 | -.2933 | -.2257 |
| | | | CLE+I/R | -.14467* | .01584 | .000 | -.1784 | -.1109 |
| | | I/R | S | .25950* | .01584 | .000 | .2257 | .2933 |
| | | | CLE+I/R | .11483* | .01584 | .000 | .0811 | .1486 |
| | | CLE+I/R | S | .14467* | .01584 | .000 | .1109 | .1784 |
| | | | I/R | -.11483* | .01584 | .000 | -.1486 | -.0811 |

*. The mean difference is significant at the 0.05 level.

Homogeneous Subsets

## FIG.2B

| | Groups | N | Subset for alpha = 0.05 | | |
|---|---|---|---|---|---|
| | | | 1 | 2 | 3 |
| Student-Newman-Keuls[a] | I/R | 3 | .68475 | | |
| | CLE+I/R | 3 | | .77412 | |
| | S | 3 | | | .88975 |
| | Sig. | | 1.000 | 1.000 | 1.000 |

Means for groups in homogeneous subsets are displayed.
a. Uses Harmonic Mean Sample Size = 3.000.

## FIG.2C

| | Groups | N | Subset for alpha = 0.05 | | |
|---|---|---|---|---|---|
| | | | 1 | 2 | 3 |
| Student-Newman-Keuls[a] | I/R | 3 | .29395 | | |
| | CLE+I/R | 3 | | .37694 | |
| | S | 3 | | | .49842 |
| | Sig. | | 1.000 | 1.000 | 1.000 |

Means for groups in homogeneous subsets are displayed.
a. Uses Harmonic Mean Sample Size = 3.000.

FIG.2D

| | Groups | N | Subset for alpha = 0.05 | | |
|---|---|---|---|---|---|
| | | | 1 | 2 | 3 |
| Student-Newman-Keuls[a] | S | 6 | .1667 | | |
| | CLE+I/R | 6 | | 3.3333 | |
| | I/R | 6 | | | 5.5000 |
| | Sig. | | 1.000 | 1.000 | 1.000 |

Means for groups in homogeneous subsets are displayed.
a. Uses Harmonic Mean Sample Size = 6.000.

FIG.2E

| | Groups | N | Subset for alpha = 0.05 | |
|---|---|---|---|---|
| | | | 1 | 2 |
| Student-Newman-Keuls[a] | S | 3 | .0000 | |
| | CLE+I/R | 3 | | .8143 |
| | I/R | 3 | | .8583 |
| | Sig. | | 1.000 | .295 |

Means for groups in homogeneous subsets are displayed.
a. Uses Harmonic Mean Sample Size = 3.000.

FIG.2G

| | Groups | N | Subset for alpha = 0.05 | | |
|---|---|---|---|---|---|
| | | | 1 | 2 | 3 |
| Student-Newman-Keuls[a] | S | 3 | .05047 | | |
| | CLE+I/R | 3 | | .33333 | |
| | I/R | 3 | | | .50333 |
| | Sig. | | 1.000 | 1.000 | 1.000 |

Means for groups in homogeneous subsets are displayed.
a. Uses Harmonic Mean Sample Size = 3.000.

FIG.2I

| Groups | N | Subset for alpha = 0.05 | | |
|---|---|---|---|---|
| | | 1 | 2 | 3 |

| | | | | | |
|---|---|---|---|---|---|
| Student-Newman-Keuls[a] | S | 6 | 1.1092 | | |
| | CLE+I/R | 6 | | 1.2538 | |
| | I/R | 6 | | | 1.3687 |
| | Sig. | | 1.000 | 1.000 | 1.000 |

Means for groups in homogeneous subsets are displayed.

a. Uses Harmonic Mean Sample Size = 6.000.
