# Supplementary material for: Clemastine Fumarate Attenuates Myocardial Ischemia Reperfusion Injury Through Inhibition of Mast Cell Degranulation
Source: Front Pharmacol. 2021 Aug 27;12:704852. doi: 10.3389/fphar.2021.704852 (PMC8430029; doi:10.3389/fphar.2021.704852)
Supplement: Supplementary file 1 [file DataSheet1.ZIP › supplementary/Data Analysis/Figure 3.pdf]

Oneway

Descriptives

		N	Mean	Std. Deviation	Std. Error	95% Confidence Interval for Mean
						Lower Bound
FIG.4B	S	6	.0667	.05164	.02108	.0125
	I/R	6	.6683	.03920	.01600	.6272
	CLE+I/R	6	.3800	.04147	.01693	.3365
	C48/80+I/R	6	.7783	.05636	.02301	.7192
	CLE+C48/80+I/R	6	.5350	.05577	.02277	.4765
	Total	30	.4857	.25665	.04686	.3898
FIG.4D	S	6	.1833	.04676	.01909	.1343
	I/R	6	.3700	.02966	.01211	.3389
	CLE+I/R	6	.2400	.03098	.01265	.2075
	C48/80+I/R	6	.4683	.04446	.01815	.4217
	CLE+C48/80+I/R	6	.2917	.03971	.01621	.2500
	Total	30	.3107	.10789	.01970	.2704
FIG.4E	S	6	1.0000	.00000	.00000	1.0000
	I/R	6	24.5333	8.11007	3.31092	16.0223
	CLE+I/R	6	9.1417	1.56148	.63747	7.5030
	C48/80+I/R	6	57.9733	5.96278	2.43429	51.7158
	CLE+C48/80+I/R	6	24.7533	5.52057	2.25377	18.9598
	Total	30	23.4803	20.41971	3.72811	15.8555
FIG.4F	S	6	1.0000	.00000	.00000	1.0000
	I/R	6	4.8662	1.13148	.46192	3.6788
	CLE+I/R	6	2.8110	.76418	.31197	2.0090
	C48/80+I/R	6	8.5000	.64290	.26246	7.8253
	CLE+C48/80+I/R	6	3.8773	.95736	.39084	2.8726
	Total	30	4.2109	2.64712	.48330	3.2224
FIG.4G	S	6	1.0000	.00000	.00000	1.0000
	I/R	6	5.8612	.57538	.23490	5.2573
	CLE+I/R	6	3.6542	.35954	.14678	3.2769
	C48/80+I/R	6	10.6210	1.14488	.46739	9.4195
	CLE+C48/80+I/R	6	5.3702	.49723	.20299	4.8484
	Total	30	5.3013	3.26466	.59604	4.0823

Descriptives

		95% Confidence Interval for Mean Upper Bound	Minimum	Maximum
FIG.4B	S	.1209	.00	.10
	I/R	.7095	.60	.71
	CLE+I/R	.4235	.33	.42
	C48/80+I/R	.8375	.70	.86
	CLE+C48/80+I/R	.5935	.46	.60
	Total	.5815	.00	.86
FIG.4D	S	.2324	.10	.24
	I/R	.4011	.32	.41
	CLE+I/R	.2725	.21	.29
	C48/80+I/R	.5150	.41	.52
	CLE+C48/80+I/R	.3333	.24	.34
	Total	.3510	.10	.52
FIG.4E	S	1.0000	1.00	1.00
	I/R	33.0443	8.12	29.39
	CLE+I/R	10.7803	7.76	11.49
	C48/80+I/R	64.2309	50.00	67.81
	CLE+C48/80+I/R	30.5468	20.71	35.81
	Total	31.1052	1.00	67.81
FIG.4F	S	1.0000	1.00	1.00
	I/R	6.0536	2.67	5.78
	CLE+I/R	3.6130	1.92	3.95
	C48/80+I/R	9.1747	7.45	9.03
	CLE+C48/80+I/R	4.8820	2.44	5.29
	Total	5.1994	1.00	9.03
FIG.4G	S	1.0000	1.00	1.00
	I/R	6.4650	5.19	6.60
	CLE+I/R	4.0315	3.23	4.25
	C48/80+I/R	11.8225	9.71	12.80
	CLE+C48/80+I/R	5.8920	4.76	5.98
	Total	6.5203	1.00	12.80

ANOVA

		Sum of Squares	df	Mean Square	F	Sig.
FIG.4B	Between Groups	1.849	4	.462	189.300	.000

	Within Groups	.061	25	.002		
	Total	1.910	29			
FIG.4D	Between Groups	.300	4	.075	49.421	.000
	Within Groups	.038	25	.002		
	Total	.338	29			
FIG.4E	Between Groups	11420.755	4	2855.189	106.344	.000
	Within Groups	671.215	25	26.849		
	Total	12091.969	29			
FIG.4F	Between Groups	187.240	4	46.810	73.276	.000
	Within Groups	15.970	25	.639		
	Total	203.210	29			
FIG.4G	Between Groups	298.990	4	74.747	185.174	.000
	Within Groups	10.092	25	.404		
	Total	309.081	29			

Post Hoc Tests

Multiple Comparisons

Dependent Variable		(I) Groups	(J) Groups	Mean Difference (I-J)	Std. Error	Sig.	95% Confidence Interval Lower Bound	Upper Bound
FIG.4B	LSD	S	I/R	-.60167*	.02853	.000	-.6604	-.5429
			CLE+I/R	-.31333*	.02853	.000	-.3721	-.2546
			C48/80+I/R	-.71167*	.02853	.000	-.7704	-.6529
			CLE+C48/80+I/R	-.46833*	.02853	.000	-.5271	-.4096
		I/R	S	.60167*	.02853	.000	.5429	.6604
			CLE+I/R	.28833*	.02853	.000	.2296	.3471
			C48/80+I/R	-.11000*	.02853	.001	-.1688	-.0512
			CLE+C48/80+I/R	.13333*	.02853	.000	.0746	.1921
		CLE+I/R	S	.31333*	.02853	.000	.2546	.3721
			I/R	-.28833*	.02853	.000	-.3471	-.2296
			C48/80+I/R	-.39833*	.02853	.000	-.4571	-.3396
			CLE+C48/80+I/R	-.15500*	.02853	.000	-.2138	-.0962
		C48/80+I/R	S	.71167*	.02853	.000	.6529	.7704

			I/R	.11000*	.02853	.001	.0512	.1688
			CLE+I/R	.39833*	.02853	.000	.3396	.4571
			CLE+C48/80+I/R	.24333*	.02853	.000	.1846	.3021
		CLE+C48/80+I/R	S	.46833*	.02853	.000	.4096	.5271
			I/R	-.13333*	.02853	.000	-.1921	-.0746
			CLE+I/R	.15500*	.02853	.000	.0962	.2138
			C48/80+I/R	-.24333*	.02853	.000	-.3021	-.1846
FIG.4D	LSD	S	I/R	-.18667*	.02248	.000	-.2330	-.1404
			CLE+I/R	-.05667*	.02248	.018	-.1030	-.0104
			C48/80+I/R	-.28500*	.02248	.000	-.3313	-.2387
			CLE+C48/80+I/R	-.10833*	.02248	.000	-.1546	-.0620
		I/R	S	.18667*	.02248	.000	.1404	.2330
			CLE+I/R	.13000*	.02248	.000	.0837	.1763
			C48/80+I/R	-.09833*	.02248	.000	-.1446	-.0520
			CLE+C48/80+I/R	.07833*	.02248	.002	.0320	.1246
		CLE+I/R	S	.05667*	.02248	.018	.0104	.1030
			I/R	-.13000*	.02248	.000	-.1763	-.0837
			C48/80+I/R	-.22833*	.02248	.000	-.2746	-.1820
			CLE+C48/80+I/R	-.05167*	.02248	.030	-.0980	-.0054
		C48/80+I/R	S	.28500*	.02248	.000	.2387	.3313
			I/R	.09833*	.02248	.000	.0520	.1446
			CLE+I/R	.22833*	.02248	.000	.1820	.2746
			CLE+C48/80+I/R	.17667*	.02248	.000	.1304	.2230
		CLE+C48/80+I/R	S	.10833*	.02248	.000	.0620	.1546
			I/R	-.07833*	.02248	.002	-.1246	-.0320
			CLE+I/R	.05167*	.02248	.030	.0054	.0980
			C48/80+I/R	-.17667*	.02248	.000	-.2230	-.1304
FIG.4E	LSD	S	I/R	-23.53333*	2.99158	.000	-29.6946	-17.3721
			CLE+I/R	-8.14167*	2.99158	.012	-14.3029	-1.9804
			C48/80+I/R	-56.97333*	2.99158	.000	-63.1346	-50.8121
			CLE+C48/80+I/R	-23.75333*	2.99158	.000	-29.9146	-17.5921
		I/R	S	23.53333*	2.99158	.000	17.3721	29.6946
			CLE+I/R	15.39167*	2.99158	.000	9.2304	21.5529
			C48/80+I/R	-33.44000*	2.99158	.000	-39.6013	-27.2787

			CLE+C48/80+I/R	-.22000	2.99158	.942	-6.3813	5.9413
		CLE+I/R	S	8.14167*	2.99158	.012	1.9804	14.3029
			I/R	-15.39167*	2.99158	.000	-21.5529	-9.2304
			C48/80+I/R	-48.83167*	2.99158	.000	-54.9929	-42.6704
			CLE+C48/80+I/R	-15.61167*	2.99158	.000	-21.7729	-9.4504
		C48/80+I/R	S	56.97333*	2.99158	.000	50.8121	63.1346
			I/R	33.44000*	2.99158	.000	27.2787	39.6013
			CLE+I/R	48.83167*	2.99158	.000	42.6704	54.9929
			CLE+C48/80+I/R	33.22000*	2.99158	.000	27.0587	39.3813
		CLE+C48/80+I/R	S	23.75333*	2.99158	.000	17.5921	29.9146
			I/R	.22000	2.99158	.942	-5.9413	6.3813
			CLE+I/R	15.61167*	2.99158	.000	9.4504	21.7729
			C48/80+I/R	-33.22000*	2.99158	.000	-39.3813	-27.0587
FIG.4F	LSD	S	I/R	-3.86617*	.46145	.000	-4.8165	-2.9158
			CLE+I/R	-1.81100*	.46145	.001	-2.7614	-.8606
			C48/80+I/R	-7.50000*	.46145	.000	-8.4504	-6.5496
			CLE+C48/80+I/R	-2.87733*	.46145	.000	-3.8277	-1.9270
		I/R	S	3.86617*	.46145	.000	2.9158	4.8165
			CLE+I/R	2.05517*	.46145	.000	1.1048	3.0055
			C48/80+I/R	-3.63383*	.46145	.000	-4.5842	-2.6835
			CLE+C48/80+I/R	.98883*	.46145	.042	.0385	1.9392
		CLE+I/R	S	1.81100*	.46145	.001	.8606	2.7614
			I/R	-2.05517*	.46145	.000	-3.0055	-1.1048
			C48/80+I/R	-5.68900*	.46145	.000	-6.6394	-4.7386
			CLE+C48/80+I/R	-1.06633*	.46145	.029	-2.0167	-.1160
		C48/80+I/R	S	7.50000*	.46145	.000	6.5496	8.4504
			I/R	3.63383*	.46145	.000	2.6835	4.5842
			CLE+I/R	5.68900*	.46145	.000	4.7386	6.6394
			CLE+C48/80+I/R	4.62267*	.46145	.000	3.6723	5.5730
		CLE+C48/80+I/R	S	2.87733*	.46145	.000	1.9270	3.8277
			I/R	-.98883*	.46145	.042	-1.9392	-.0385
			CLE+I/R	1.06633*	.46145	.029	.1160	2.0167
			C48/80+I/R	-4.62267*	.46145	.000	-5.5730	-3.6723
FIG.4G	LSD	S	I/R	-4.86117*	.36682	.000	-5.6166	-4.1057

		CLE+I/R	-2.65417*	.36682	.000	-3.4096	-1.8987
		C48/80+I/R	-9.62100*	.36682	.000	-10.3765	-8.8655
		CLE+C48/80+I/R	-4.37017*	.36682	.000	-5.1256	-3.6147
	I/R	S	4.86117*	.36682	.000	4.1057	5.6166
		CLE+I/R	2.20700*	.36682	.000	1.4515	2.9625
		C48/80+I/R	-4.75983*	.36682	.000	-5.5153	-4.0044
		CLE+C48/80+I/R	.49100	.36682	.193	-.2645	1.2465
	CLE+I/R	S	2.65417*	.36682	.000	1.8987	3.4096
		I/R	-2.20700*	.36682	.000	-2.9625	-1.4515
		C48/80+I/R	-6.96683*	.36682	.000	-7.7223	-6.2114
		CLE+C48/80+I/R	-1.71600*	.36682	.000	-2.4715	-.9605
	C48/80+I/R	S	9.62100*	.36682	.000	8.8655	10.3765
		I/R	4.75983*	.36682	.000	4.0044	5.5153
		CLE+I/R	6.96683*	.36682	.000	6.2114	7.7223
		CLE+C48/80+I/R	5.25083*	.36682	.000	4.4954	6.0063
	CLE+C48/80+I/R	S	4.37017*	.36682	.000	3.6147	5.1256
		I/R	-.49100	.36682	.193	-1.2465	.2645
		CLE+I/R	1.71600*	.36682	.000	.9605	2.4715
		C48/80+I/R	-5.25083*	.36682	.000	-6.0063	-4.4954

*. The mean difference is significant at the 0.05 level.

Homogeneous Subsets

FIG.4B

	Groups	N	Subset for alpha = 0.05		
			1	2	3
Student-Newman-Keuls[a]	S	6	.0667		
	CLE+I/R	6		.3800	
	CLE+C48/80+I/R	6			.5350
	I/R	6			
	C48/80+I/R	6			
	Sig.		1.000	1.000	1.000

FIG.4B

	Groups	Subset for alpha = 0.05 4	5
Student-Newman-Keuls[a]	S		
	CLE+I/R		
	CLE+C48/80+I/R		
	I/R	.6683	
	C48/80+I/R		.7783
	Sig.	1.000	1.000

Means for groups in homogeneous subsets are displayed.
a. Uses Harmonic Mean Sample Size = 6.000.

FIG.4D

	Groups	N	Subset for alpha = 0.05 1	2	3
Student-Newman-Keuls[a]	S	6	.1833		
	CLE+I/R	6		.2400	
	CLE+C48/80+I/R	6			.2917
	I/R	6			
	C48/80+I/R	6			
	Sig.		1.000	1.000	1.000

FIG.4D

	Groups	Subset for alpha = 0.05 4	5
Student-Newman-Keuls[a]	S		
	CLE+I/R		
	CLE+C48/80+I/R		
	I/R	.3700	
	C48/80+I/R		.4683
	Sig.	1.000	1.000

Means for groups in homogeneous subsets are displayed.
a. Uses Harmonic Mean Sample Size = 6.000.

FIG.4E

Groups	N	Subset for alpha = 0.05 1	2	3

Student-Newman-Keuls[a]	S	6	1.0000		
	CLE+I/R	6		9.1417	
	I/R	6			24.5333
	CLE+C48/80+I/R	6			24.7533
	C48/80+I/R	6			
	Sig.		1.000	1.000	.942

FIG.4E

	Groups	Subset for alpha = 0.05
		4
Student-Newman-Keuls[a]	S	
	CLE+I/R	
	I/R	
	CLE+C48/80+I/R	
	C48/80+I/R	57.9733
	Sig.	1.000

Means for groups in homogeneous subsets are displayed.
a. Uses Harmonic Mean Sample Size = 6.000.

FIG.4F

	Groups	N	Subset for alpha = 0.05		
			1	2	3
Student-Newman-Keuls[a]	S	6	1.0000		
	CLE+I/R	6		2.8110	
	CLE+C48/80+I/R	6			3.8773
	I/R	6			
	C48/80+I/R	6			
	Sig.		1.000	1.000	1.000

FIG.4F

	Groups	Subset for alpha = 0.05	
		4	5
Student-Newman-Keuls[a]	S		
	CLE+I/R		
	CLE+C48/80+I/R		
	I/R	4.8662	
	C48/80+I/R		8.5000
	Sig.	1.000	1.000

Means for groups in homogeneous subsets are displayed.

a. Uses Harmonic Mean Sample Size = 6.000.

FIG.4G

	Groups	N	Subset for alpha = 0.05		
			1	2	3
Student-Newman-Keuls[a]	S	6	1.0000		
	CLE+I/R	6		3.6542	
	CLE+C48/80+I/R	6			5.3702
	I/R	6			5.8612
	C48/80+I/R	6			
	Sig.		1.000	1.000	.193

FIG.4G

	Groups	Subset for alpha = 0.05
		4
Student-Newman-Keuls[a]	S	
	CLE+I/R	
	CLE+C48/80+I/R	
	I/R	
	C48/80+I/R	10.6210
	Sig.	1.000

Means for groups in homogeneous subsets are displayed.

a. Uses Harmonic Mean Sample Size = 6.000.
