# Supplementary material for: Clemastine Fumarate Attenuates Myocardial Ischemia Reperfusion Injury Through Inhibition of Mast Cell Degranulation
Source: Front Pharmacol. 2021 Aug 27;12:704852. doi: 10.3389/fphar.2021.704852 (PMC8430029; doi:10.3389/fphar.2021.704852)
Supplement: Supplementary file 1 [file DataSheet1.ZIP › supplementary/Data Analysis/Figure 4.pdf]

Oneway

Descriptives

		N	Mean	Std. Deviation	Std. Error	95% Confidence Interval for Mean Lower Bound	Upper Bound
FIG.4B	S	3	.88847	.011675	.006741	.85946	.91747
	I/R	3	.67543	.008110	.004682	.65529	.69558
	CLE+I/R	3	.78643	.020905	.012070	.73450	.83836
	C48/80+I/R	3	.52083	.021149	.012210	.46830	.57337
	CLE+C48/80+I/R	3	.75377	.007925	.004576	.73408	.77346
	Total	15	.72499	.127809	.033000	.65421	.79577
FIG.4C	S	3	.49123	.013423	.007750	.45789	.52458
	I/R	3	.29354	.009156	.005286	.27080	.31629
	CLE+I/R	3	.38393	.011784	.006804	.35466	.41321
	C48/80+I/R	3	.22853	.012756	.007364	.19685	.26022
	CLE+C48/80+I/R	3	.33957	.006396	.003692	.32368	.35545
	Total	15	.34736	.092023	.023760	.29640	.39832
FIG.4D	S	3	1.4433	.08622	.04978	1.2292	1.6575
	I/R	3	3.0783	.10128	.05848	2.8267	3.3299
	CLE+I/R	3	2.2490	.06718	.03879	2.0821	2.4159
	C48/80+I/R	3	4.5407	.13191	.07616	4.2130	4.8684
	CLE+C48/80+I/R	3	3.1130	.21045	.12150	2.5902	3.6358
	Total	15	2.8849	1.07266	.27696	2.2908	3.4789
FIG.4E	S	3	4.7493	.10362	.05982	4.4919	5.0067
	I/R	3	5.6833	.11015	.06360	5.4097	5.9570
	CLE+I/R	3	5.0233	.10116	.05840	4.7720	5.2746
	C48/80+I/R	3	6.7493	.11828	.06829	6.4555	7.0431
	CLE+C48/80+I/R	3	5.5337	.08556	.04940	5.3211	5.7462
	Total	15	5.5478	.71880	.18559	5.1497	5.9459
FIG.4F	S	3	5.5767	.86031	.49670	3.4395	7.7138
	I/R	3	38.4067	1.31390	.75858	35.1428	41.6706
	CLE+I/R	3	17.4017	1.01905	.58835	14.8702	19.9331
	C48/80+I/R	3	88.2570	4.78741	2.76401	76.3644	100.1496
	CLE+C48/80+I/R	3	33.7130	2.76447	1.59607	26.8457	40.5803
	Total	15	36.6710	29.40027	7.59112	20.3897	52.9523
FIG.4G	S	3	92.6113	4.24151	2.44884	82.0748	103.1478
	I/R	3	157.9753	5.20469	3.00493	145.0462	170.9045
	CLE+I/R	3	120.3833	4.83046	2.78887	108.3838	132.3829
	C48/80+I/R	3	225.9277	8.83739	5.10227	203.9744	247.8810

CLE+C48/80+I/R	3	136.5167	18.71915	10.80751	90.0157	183.0176
Total	15	146.6829	47.33758	12.22251	120.4682	172.8975

Descriptives

		Minimum	Maximum
FIG.4B	S	.878	.901
	I/R	.667	.683
	CLE+I/R	.770	.810
	C48/80+I/R	.502	.544
	CLE+C48/80+I/R	.747	.763
	Total	.502	.901
FIG.4C	S	.476	.502
	I/R	.287	.304
	CLE+I/R	.373	.396
	C48/80+I/R	.214	.237
	CLE+C48/80+I/R	.334	.347
	Total	.214	.502
FIG.4D	S	1.35	1.52
	I/R	2.97	3.16
	CLE+I/R	2.18	2.31
	C48/80+I/R	4.44	4.69
	CLE+C48/80+I/R	2.95	3.35
	Total	1.35	4.69
FIG.4E	S	4.64	4.85
	I/R	5.61	5.81
	CLE+I/R	4.96	5.14
	C48/80+I/R	6.64	6.87
	CLE+C48/80+I/R	5.45	5.62
	Total	4.64	6.87
FIG.4F	S	4.65	6.35
	I/R	37.15	39.77
	CLE+I/R	16.38	18.42
	C48/80+I/R	82.90	92.12
	CLE+C48/80+I/R	30.83	36.34
	Total	4.65	92.12
FIG.4G	S	88.64	97.08
	I/R	154.48	163.96
	CLE+I/R	116.11	125.63
	C48/80+I/R	216.77	234.40
	CLE+C48/80+I/R	115.03	149.27

Total		88.64	234.40

ANOVA

		Sum of Squares	df	Mean Square	F	Sig.
FIG.4B	Between Groups	.226	4	.057	246.255	.000
	Within Groups	.002	10	.000		
	Total	.229	14			
FIG.4C	Between Groups	.117	4	.029	241.849	.000
	Within Groups	.001	10	.000		
	Total	.119	14			
FIG.4D	Between Groups	15.940	4	3.985	237.506	.000
	Within Groups	.168	10	.017		
	Total	16.108	14			
FIG.4E	Between Groups	7.125	4	1.781	163.670	.000
	Within Groups	.109	10	.011		
	Total	7.233	14			
FIG.4F	Between Groups	12033.127	4	3008.282	441.531	.000
	Within Groups	68.133	10	6.813		
	Total	12101.260	14			
FIG.4G	Between Groups	30378.018	4	7594.504	76.416	.000
	Within Groups	993.837	10	99.384		
	Total	31371.855	14			

Post Hoc Tests

Multiple Comparisons

Dependent Variable		(I) Groups	(J) Groups	Mean Difference (I-J)	Std. Error	Sig.	95% Confidence Interval Lower Bound	Upper Bound
FIG.4B	LSD	S	I/R	.213033*	.012378	.000	.18545	.24061
			CLE+I/R	.102033*	.012378	.000	.07445	.12961
			C48/80+I/R	.367633*	.012378	.000	.34005	.39521
			CLE+C48/80+I/R	.134697*	.012378	.000	.10712	.16228
		I/R	S	-.213033*	.012378	.000	-.24061	-.18545
			CLE+I/R	-.111000*	.012378	.000	-.13858	-.08342

			C48/80+I/R	.154600*	.012378	.000	.12702	.18218
			CLE+C48/80+I/R	-.078337*	.012378	.000	-.10592	-.05076
		CLE+I/R	S	-.102033*	.012378	.000	-.12961	-.07445
			I/R	.111000*	.012378	.000	.08342	.13858
			C48/80+I/R	.265600*	.012378	.000	.23802	.29318
			CLE+C48/80+I/R	.032663*	.012378	.025	.00508	.06024
		C48/80+I/R	S	-.367633*	.012378	.000	-.39521	-.34005
			I/R	-.154600*	.012378	.000	-.18218	-.12702
			CLE+I/R	-.265600*	.012378	.000	-.29318	-.23802
			CLE+C48/80+I/R	-.232937*	.012378	.000	-.26052	-.20536
		CLE+C48/80+I/R	S	-.134697*	.012378	.000	-.16228	-.10712
			I/R	.078337*	.012378	.000	.05076	.10592
			CLE+I/R	-.032663*	.012378	.025	-.06024	-.00508
			C48/80+I/R	.232937*	.012378	.000	.20536	.26052
FIG.4C	LSD	S	I/R	.197690*	.008992	.000	.17765	.21773
			CLE+I/R	.107300*	.008992	.000	.08726	.12734
			C48/80+I/R	.262700*	.008992	.000	.24266	.28274
			CLE+C48/80+I/R	.151667*	.008992	.000	.13163	.17170
		I/R	S	-.197690*	.008992	.000	-.21773	-.17765
			CLE+I/R	-.090390*	.008992	.000	-.11043	-.07035
			C48/80+I/R	.065010*	.008992	.000	.04497	.08505
			CLE+C48/80+I/R	-.046023*	.008992	.000	-.06606	-.02599
		CLE+I/R	S	-.107300*	.008992	.000	-.12734	-.08726
			I/R	.090390*	.008992	.000	.07035	.11043
			C48/80+I/R	.155400*	.008992	.000	.13536	.17544
			CLE+C48/80+I/R	.044367*	.008992	.001	.02433	.06440
		C48/80+I/R	S	-.262700*	.008992	.000	-.28274	-.24266
			I/R	-.065010*	.008992	.000	-.08505	-.04497
			CLE+I/R	-.155400*	.008992	.000	-.17544	-.13536
			CLE+C48/80+I/R	-.111033*	.008992	.000	-.13107	-.09100
		CLE+C48/80+I/R	S	-.151667*	.008992	.000	-.17170	-.13163
			I/R	.046023*	.008992	.000	.02599	.06606
			CLE+I/R	-.044367*	.008992	.001	-.06440	-.02433
			C48/80+I/R	.111033*	.008992	.000	.09100	.13107

FIG.4D	LSD	S	I/R	-1.63500*	.10576	.000	-1.8707	-1.3993
			CLE+I/R	-.80567*	.10576	.000	-1.0413	-.5700
			C48/80+I/R	-3.09733*	.10576	.000	-3.3330	-2.8617
			CLE+C48/80+I/R	-1.66967*	.10576	.000	-1.9053	-1.4340
		I/R	S	1.63500*	.10576	.000	1.3993	1.8707
			CLE+I/R	.82933*	.10576	.000	.5937	1.0650
			C48/80+I/R	-1.46233*	.10576	.000	-1.6980	-1.2267
			CLE+C48/80+I/R	-.03467	.10576	.750	-.2703	.2010
		CLE+I/R	S	.80567*	.10576	.000	.5700	1.0413
			I/R	-.82933*	.10576	.000	-1.0650	-.5937
			C48/80+I/R	-2.29167*	.10576	.000	-2.5273	-2.0560
			CLE+C48/80+I/R	-.86400*	.10576	.000	-1.0997	-.6283
		C48/80+I/R	S	3.09733*	.10576	.000	2.8617	3.3330
			I/R	1.46233*	.10576	.000	1.2267	1.6980
			CLE+I/R	2.29167*	.10576	.000	2.0560	2.5273
			CLE+C48/80+I/R	1.42767*	.10576	.000	1.1920	1.6633
		CLE+C48/80+I/R	S	1.66967*	.10576	.000	1.4340	1.9053
			I/R	.03467	.10576	.750	-.2010	.2703
			CLE+I/R	.86400*	.10576	.000	.6283	1.0997
			C48/80+I/R	-1.42767*	.10576	.000	-1.6633	-1.1920
FIG.4E	LSD	S	I/R	-.93400*	.08518	.000	-1.1238	-.7442
			CLE+I/R	-.27400*	.08518	.009	-.4638	-.0842
			C48/80+I/R	-2.00000*	.08518	.000	-2.1898	-1.8102
			CLE+C48/80+I/R	-.78433*	.08518	.000	-.9741	-.5945
		I/R	S	.93400*	.08518	.000	.7442	1.1238
			CLE+I/R	.66000*	.08518	.000	.4702	.8498
			C48/80+I/R	-1.06600*	.08518	.000	-1.2558	-.8762
			CLE+C48/80+I/R	.14967	.08518	.109	-.0401	.3395
		CLE+I/R	S	.27400*	.08518	.009	.0842	.4638
			I/R	-.66000*	.08518	.000	-.8498	-.4702
			C48/80+I/R	-1.72600*	.08518	.000	-1.9158	-1.5362
			CLE+C48/80+I/R	-.51033*	.08518	.000	-.7001	-.3205
		C48/80+I/R	S	2.00000*	.08518	.000	1.8102	2.1898
			I/R	1.06600*	.08518	.000	.8762	1.2558

			CLE+I/R	1.72600*	.08518	.000	1.5362	1.9158
			CLE+C48/80+I/R	1.21567*	.08518	.000	1.0259	1.4055
		CLE+C48/80+I/R	S	.78433*	.08518	.000	.5945	.9741
			I/R	-.14967	.08518	.109	-.3395	.0401
			CLE+I/R	.51033*	.08518	.000	.3205	.7001
			C48/80+I/R	-1.21567*	.08518	.000	-1.4055	-1.0259
FIG.4F	LSD	S	I/R	-32.83000*	2.13124	.000	-37.5787	-28.0813
			CLE+I/R	-11.82500*	2.13124	.000	-16.5737	-7.0763
			C48/80+I/R	-82.68033*	2.13124	.000	-87.4290	-77.9316
			CLE+C48/80+I/R	-28.13633*	2.13124	.000	-32.8850	-23.3876
		I/R	S	32.83000*	2.13124	.000	28.0813	37.5787
			CLE+I/R	21.00500*	2.13124	.000	16.2563	25.7537
			C48/80+I/R	-49.85033*	2.13124	.000	-54.5990	-45.1016
			CLE+C48/80+I/R	4.69367	2.13124	.052	-.0550	9.4424
		CLE+I/R	S	11.82500*	2.13124	.000	7.0763	16.5737
			I/R	-21.00500*	2.13124	.000	-25.7537	-16.2563
			C48/80+I/R	-70.85533*	2.13124	.000	-75.6040	-66.1066
			CLE+C48/80+I/R	-16.31133*	2.13124	.000	-21.0600	-11.5626
		C48/80+I/R	S	82.68033*	2.13124	.000	77.9316	87.4290
			I/R	49.85033*	2.13124	.000	45.1016	54.5990
			CLE+I/R	70.85533*	2.13124	.000	66.1066	75.6040
			CLE+C48/80+I/R	54.54400*	2.13124	.000	49.7953	59.2927
		CLE+C48/80+I/R	S	28.13633*	2.13124	.000	23.3876	32.8850
			I/R	-4.69367	2.13124	.052	-9.4424	.0550
			CLE+I/R	16.31133*	2.13124	.000	11.5626	21.0600
			C48/80+I/R	-54.54400*	2.13124	.000	-59.2927	-49.7953
FIG.4G	LSD	S	I/R	-65.36400*	8.13977	.000	-83.5005	-47.2275
			CLE+I/R	-27.77200*	8.13977	.007	-45.9085	-9.6355
			C48/80+I/R	-133.31633*	8.13977	.000	-151.4529	-115.1798
			CLE+C48/80+I/R	-43.90533*	8.13977	.000	-62.0419	-25.7688
		I/R	S	65.36400*	8.13977	.000	47.2275	83.5005
			CLE+I/R	37.59200*	8.13977	.001	19.4555	55.7285
			C48/80+I/R	-67.95233*	8.13977	.000	-86.0889	-49.8158
			CLE+C48/80+I/R	21.45867*	8.13977	.025	3.3221	39.5952

	CLE+I/R	S	27.77200*	8.13977	.007	9.6355	45.9085
		I/R	-37.59200*	8.13977	.001	-55.7285	-19.4555
		C48/80+I/R	-105.54433*	8.13977	.000	-123.6809	-87.4078
		CLE+C48/80+I/R	-16.13333	8.13977	.076	-34.2699	2.0032
	C48/80+I/R	S	133.31633*	8.13977	.000	115.1798	151.4529
		I/R	67.95233*	8.13977	.000	49.8158	86.0889
		CLE+I/R	105.54433*	8.13977	.000	87.4078	123.6809
		CLE+C48/80+I/R	89.41100*	8.13977	.000	71.2745	107.5475
	CLE+C48/80+I/R	S	43.90533*	8.13977	.000	25.7688	62.0419
		I/R	-21.45867*	8.13977	.025	-39.5952	-3.3221
		CLE+I/R	16.13333	8.13977	.076	-2.0032	34.2699
		C48/80+I/R	-89.41100*	8.13977	.000	-107.5475	-71.2745

*. The mean difference is significant at the 0.05 level.

Homogeneous Subsets

FIG.4B

	Groups	N	Subset for alpha = 0.05		
			1	2	3
Student-Newman-Keuls[a]	C48/80+I/R	3	.52083		
	I/R	3		.67543	
	CLE+C48/80+I/R	3			.75377
	CLE+I/R	3			
	S	3			
	Sig.		1.000	1.000	1.000

FIG.4B

	Groups	Subset for alpha = 0.05	
		4	5
Student-Newman-Keuls[a]	C48/80+I/R		
	I/R		
	CLE+C48/80+I/R		
	CLE+I/R	.78643	
	S		.88847
	Sig.	1.000	1.000

Means for groups in homogeneous subsets are displayed.

a. Uses Harmonic Mean Sample Size = 3.000.

FIG.4C

	Groups	N	Subset for alpha = 0.05		
			1	2	3
Student-Newman-Keuls[a]	C48/80+I/R	3	.22853		
	I/R	3		.29354	
	CLE+C48/80+I/R	3			.33957
	CLE+I/R	3			
	S	3			
	Sig.		1.000	1.000	1.000

FIG.4C

	Groups	Subset for alpha = 0.05	
		4	5
Student-Newman-Keuls[a]	C48/80+I/R		
	I/R		
	CLE+C48/80+I/R		
	CLE+I/R	.38393	
	S		.49123
	Sig.	1.000	1.000

Means for groups in homogeneous subsets are displayed.

a. Uses Harmonic Mean Sample Size = 3.000.

FIG.4D

	Groups	N	Subset for alpha = 0.05		
			1	2	3
Student-Newman-Keuls[a]	S	3	1.4433		
	CLE+I/R	3		2.2490	
	I/R	3			3.0783
	CLE+C48/80+I/R	3			3.1130
	C48/80+I/R	3			
	Sig.		1.000	1.000	.750

FIG.4D

Groups	Subset for alpha = 0.05

		4
Student-Newman-Keuls[a]	S	
	CLE+I/R	
	I/R	
	CLE+C48/80+I/R	
	C48/80+I/R	4.5407
	Sig.	1.000

Means for groups in homogeneous subsets are displayed.

a. Uses Harmonic Mean Sample Size = 3.000.

FIG.4E

	Groups	N	Subset for alpha = 0.05		
			1	2	3
Student-Newman-Keuls[a]	S	3	4.7493		
	CLE+I/R	3		5.0233	
	CLE+C48/80+I/R	3			5.5337
	I/R	3			5.6833
	C48/80+I/R	3			
	Sig.		1.000	1.000	.109

FIG.4E

	Groups	Subset for alpha = 0.05
		4
Student-Newman-Keuls[a]	S	
	CLE+I/R	
	CLE+C48/80+I/R	
	I/R	
	C48/80+I/R	6.7493
	Sig.	1.000

Means for groups in homogeneous subsets are displayed.

a. Uses Harmonic Mean Sample Size = 3.000.

FIG.4F

	Groups	N	Subset for alpha = 0.05		
			1	2	3
Student-Newman-Keuls[a]	S	3	5.5767		

	CLE+I/R	3		17.4017	
	CLE+C48/80+I/R	3			33.7130
	I/R	3			38.4067
	C48/80+I/R	3			
	Sig.		1.000	1.000	.052

FIG.4F

	Groups	Subset for alpha = 0.05
		4
Student-Newman-Keuls[a]	S	
	CLE+I/R	
	CLE+C48/80+I/R	
	I/R	
	C48/80+I/R	88.2570
	Sig.	1.000

Means for groups in homogeneous subsets are displayed.

a. Uses Harmonic Mean Sample Size = 3.000.

FIG.4G

	Groups	N	Subset for alpha = 0.05		
			1	2	3
Student-Newman-Keuls[a]	S	3	92.6113		
	CLE+I/R	3		120.3833	
	CLE+C48/80+I/R	3		136.5167	
	I/R	3			157.9753
	C48/80+I/R	3			
	Sig.		1.000	.076	1.000

FIG.4G

	Groups	Subset for alpha = 0.05
		4
Student-Newman-Keuls[a]	S	
	CLE+I/R	
	CLE+C48/80+I/R	
	I/R	
	C48/80+I/R	225.9277
	Sig.	1.000

Means for groups in homogeneous subsets are displayed.

a. Uses Harmonic Mean Sample Size = 3.000.
