# Supplementary material for: Clemastine Fumarate Attenuates Myocardial Ischemia Reperfusion Injury Through Inhibition of Mast Cell Degranulation
Source: Front Pharmacol. 2021 Aug 27;12:704852. doi: 10.3389/fphar.2021.704852 (PMC8430029; doi:10.3389/fphar.2021.704852)
Supplement: Supplementary file 1 [file DataSheet1.ZIP › supplementary/Data Analysis/Figure 5.pdf]

Oneway

Descriptives

		N	Mean	Std. Deviation	Std. Error	95% Confidence Interval for Mean Lower Bound
FIG.5B	S	6	.058838	.0235661	.0096208	.034107
	I/R	6	.496667	.0382971	.0156347	.456476
	CLE+I/R	6	.308333	.0440076	.0179660	.262150
	C48/80+I/R	6	.655000	.0628490	.0256580	.589044
	CLE+C48/80+I/R	6	.378333	.0397073	.0162104	.336663
	Total	30	.379434	.2061584	.0376392	.302453
FIG.5C	S	6	.1667	.25820	.10541	-.1043
	I/R	6	20.1667	1.83485	.74907	18.2411
	CLE+I/R	6	13.3333	1.75119	.71492	11.4956
	C48/80+I/R	6	25.1667	2.13698	.87242	22.9240
	CLE+C48/80+I/R	6	17.3333	1.86190	.76012	15.3794
	Total	30	15.2333	8.75109	1.59772	11.9656
FIG.5E	S	6	438.6667	701.87145	286.53782	-297.9022
	I/R	6	18993.9833	589.40692	240.62437	18375.4387
	CLE+I/R	6	16047.3167	826.81766	337.54690	15179.6247
	C48/80+I/R	6	25511.1667	1241.06783	506.66382	24208.7459
	CLE+C48/80+I/R	6	16770.4333	1813.27075	740.26469	14867.5224
	Total	30	15552.3133	8466.58611	1545.78007	12390.8381
FIG.5F	S	6	6.7333	1.92527	.78599	4.7129
	I/R	6	17.7167	2.07501	.84712	15.5391
	CLE+I/R	6	10.3833	1.62901	.66504	8.6738
	C48/80+I/R	6	29.0000	2.14849	.87712	26.7453
	CLE+C48/80+I/R	6	12.6750	1.42820	.58306	11.1762
	Total	30	15.3017	8.03978	1.46786	12.2996

Descriptives

		95% Confidence Interval for Mean Upper Bound	Minimum	Maximum
FIG.5B	S	.083569	.0440	.1057
	I/R	.536857	.4400	.5500
	CLE+I/R	.354516	.2500	.3800
	C48/80+I/R	.720956	.5900	.7300
	CLE+C48/80+I/R	.420004	.3100	.4100

	Total	.456415	.0440	.7300
FIG.5C	S	.4376	.00	.50
	I/R	22.0922	18.00	22.00
	CLE+I/R	15.1711	11.00	16.00
	C48/80+I/R	27.4093	23.00	29.00
	CLE+C48/80+I/R	19.2873	15.00	19.00
	Total	18.5010	.00	29.00
FIG.5E	S	1175.2356	.00	1852.60
	I/R	19612.5280	18073.00	19786.00
	CLE+I/R	16915.0086	15030.40	17126.00
	C48/80+I/R	26813.5875	23756.00	26687.00
	CLE+C48/80+I/R	18673.3443	14503.00	19107.00
	Total	18713.7885	.00	26687.00
FIG.5F	S	8.7538	3.30	8.40
	I/R	19.8943	15.40	20.60
	CLE+I/R	12.0929	8.80	13.10
	C48/80+I/R	31.2547	26.00	32.10
	CLE+C48/80+I/R	14.1738	11.20	15.00
	Total	18.3038	3.30	32.10

ANOVA

		Sum of Squares	df	Mean Square	F	Sig.
FIG.5B	Between Groups	1.185	4	.296	156.176	.000
	Within Groups	.047	25	.002		
	Total	1.233	29			
FIG.5C	Between Groups	2148.200	4	537.050	184.765	.000
	Within Groups	72.667	25	2.907		
	Total	2220.867	29			
FIG.5E	Between Groups	2047050073.818	4	511762518.455	402.845	.000
	Within Groups	31759258.442	25	1270370.338		
	Total	2078809332.260	29			
FIG.5F	Between Groups	1787.894	4	446.973	129.021	.000
	Within Groups	86.609	25	3.464		
	Total	1874.502	29			

Post Hoc Tests

Multiple Comparisons

Dependent Variable		(I) Groups	(J) Groups	Mean Difference (I-J)	Std. Error	Sig.	95% Confidence Interval Lower Bound	95% Confidence Interval Upper Bound
FIG.5B	LSD	S	I/R	-.4378287*	.0251467	.000	-.489619	-.386038
			CLE+I/R	-.2494954*	.0251467	.000	-.301286	-.197705
			C48/80+I/R	-.5961620*	.0251467	.000	-.647953	-.544371
			CLE+C48/80+I/R	-.3194954*	.0251467	.000	-.371286	-.267705
		I/R	S	.4378287*	.0251467	.000	.386038	.489619
			CLE+I/R	.1883333*	.0251467	.000	.136543	.240124
			C48/80+I/R	-.1583333*	.0251467	.000	-.210124	-.106543
			CLE+C48/80+I/R	.1183333*	.0251467	.000	.066543	.170124
		CLE+I/R	S	.2494954*	.0251467	.000	.197705	.301286
			I/R	-.1883333*	.0251467	.000	-.240124	-.136543
			C48/80+I/R	-.3466667*	.0251467	.000	-.398457	-.294876
			CLE+C48/80+I/R	-.0700000*	.0251467	.010	-.121791	-.018209
		C48/80+I/R	S	.5961620*	.0251467	.000	.544371	.647953
			I/R	.1583333*	.0251467	.000	.106543	.210124
			CLE+I/R	.3466667*	.0251467	.000	.294876	.398457
			CLE+C48/80+I/R	.2766667*	.0251467	.000	.224876	.328457
		CLE+C48/80+I/R	S	.3194954*	.0251467	.000	.267705	.371286
			I/R	-.1183333*	.0251467	.000	-.170124	-.066543
			CLE+I/R	.0700000*	.0251467	.010	.018209	.121791
			C48/80+I/R	-.2766667*	.0251467	.000	-.328457	-.224876
FIG.5C	LSD	S	I/R	-20.00000*	.98432	.000	-22.0272	-17.9728
			CLE+I/R	-13.16667*	.98432	.000	-15.1939	-11.1394
			C48/80+I/R	-25.00000*	.98432	.000	-27.0272	-22.9728
			CLE+C48/80+I/R	-17.16667*	.98432	.000	-19.1939	-15.1394
		I/R	S	20.00000*	.98432	.000	17.9728	22.0272
			CLE+I/R	6.83333*	.98432	.000	4.8061	8.8606
			C48/80+I/R	-5.00000*	.98432	.000	-7.0272	-2.9728
			CLE+C48/80+I/R	2.83333*	.98432	.008	.8061	4.8606

		CLE+I/R	S	13.16667*	.98432	.000	11.1394	15.1939
			I/R	-6.83333*	.98432	.000	-8.8606	-4.8061
			C48/80+I/R	-11.83333*	.98432	.000	-13.8606	-9.8061
			CLE+C48/80+I/R	-4.00000*	.98432	.000	-6.0272	-1.9728
		C48/80+I/R	S	25.00000*	.98432	.000	22.9728	27.0272
			I/R	5.00000*	.98432	.000	2.9728	7.0272
			CLE+I/R	11.83333*	.98432	.000	9.8061	13.8606
			CLE+C48/80+I/R	7.83333*	.98432	.000	5.8061	9.8606
		CLE+C48/80+I/R	S	17.16667*	.98432	.000	15.1394	19.1939
			I/R	-2.83333*	.98432	.008	-4.8606	-.8061
			CLE+I/R	4.00000*	.98432	.000	1.9728	6.0272
			C48/80+I/R	-7.83333*	.98432	.000	-9.8606	-5.8061
FIG.5E	LSD	S	I/R	-18555.31667*	650.73557	.000	-19895.5317	-17215.1017
			CLE+I/R	-15608.65000*	650.73557	.000	-16948.8650	-14268.4350
			C48/80+I/R	-25072.50000*	650.73557	.000	-26412.7150	-23732.2850
			CLE+C48/80+I/R	-16331.76667*	650.73557	.000	-17671.9817	-14991.5517
		I/R	S	18555.31667*	650.73557	.000	17215.1017	19895.5317
			CLE+I/R	2946.66667*	650.73557	.000	1606.4517	4286.8817
			C48/80+I/R	-6517.18333*	650.73557	.000	-7857.3983	-5176.9683
			CLE+C48/80+I/R	2223.55000*	650.73557	.002	883.3350	3563.7650
		CLE+I/R	S	15608.65000*	650.73557	.000	14268.4350	16948.8650
			I/R	-2946.66667*	650.73557	.000	-4286.8817	-1606.4517
			C48/80+I/R	-9463.85000*	650.73557	.000	-10804.0650	-8123.6350
			CLE+C48/80+I/R	-723.11667	650.73557	.277	-2063.3317	617.0983
		C48/80+I/R	S	25072.50000*	650.73557	.000	23732.2850	26412.7150

			I/R	6517.18333*	650.73557	.000	5176.9683	7857.3983
			CLE+I/R	9463.85000*	650.73557	.000	8123.6350	10804.0650
			CLE+C48/80+I/R	8740.73333*	650.73557	.000	7400.5183	10080.9483
		CLE+C48/80+I/R	S	16331.76667*	650.73557	.000	14991.5517	17671.9817
			I/R	-2223.55000*	650.73557	.002	-3563.7650	-883.3350
			CLE+I/R	723.11667	650.73557	.277	-617.0983	2063.3317
			C48/80+I/R	-8740.73333*	650.73557	.000	-10080.9483	-7400.5183
FIG.5F	LSD	S	I/R	-10.98333*	1.07461	.000	-13.1965	-8.7701
			CLE+I/R	-3.65000*	1.07461	.002	-5.8632	-1.4368
			C48/80+I/R	-22.26667*	1.07461	.000	-24.4799	-20.0535
			CLE+C48/80+I/R	-5.94167*	1.07461	.000	-8.1549	-3.7285
		I/R	S	10.98333*	1.07461	.000	8.7701	13.1965
			CLE+I/R	7.33333*	1.07461	.000	5.1201	9.5465
			C48/80+I/R	-11.28333*	1.07461	.000	-13.4965	-9.0701
			CLE+C48/80+I/R	5.04167*	1.07461	.000	2.8285	7.2549
		CLE+I/R	S	3.65000*	1.07461	.002	1.4368	5.8632
			I/R	-7.33333*	1.07461	.000	-9.5465	-5.1201
			C48/80+I/R	-18.61667*	1.07461	.000	-20.8299	-16.4035
			CLE+C48/80+I/R	-2.29167*	1.07461	.043	-4.5049	-.0785
		C48/80+I/R	S	22.26667*	1.07461	.000	20.0535	24.4799
			I/R	11.28333*	1.07461	.000	9.0701	13.4965
			CLE+I/R	18.61667*	1.07461	.000	16.4035	20.8299
			CLE+C48/80+I/R	16.32500*	1.07461	.000	14.1118	18.5382
		CLE+C48/80+I/R	S	5.94167*	1.07461	.000	3.7285	8.1549
			I/R	-5.04167*	1.07461	.000	-7.2549	-2.8285
			CLE+I/R	2.29167*	1.07461	.043	.0785	4.5049
			C48/80+I/R	-16.32500*	1.07461	.000	-18.5382	-14.1118

*. The mean difference is significant at the 0.05 level.

Homogeneous Subsets

FIG.5B

	Groups	N	Subset for alpha = 0.05 1	2	3
Student-Newman-Keuls[a]	S	6	.058838		
	CLE+I/R	6		.308333	
	CLE+C48/80+I/R	6			.378333
	I/R	6			
	C48/80+I/R	6			
	Sig.		1.000	1.000	1.000

FIG.5B

	Groups	Subset for alpha = 0.05 4	5
Student-Newman-Keuls[a]	S		
	CLE+I/R		
	CLE+C48/80+I/R		
	I/R	.496667	
	C48/80+I/R		.655000
	Sig.	1.000	1.000

Means for groups in homogeneous subsets are displayed.

a. Uses Harmonic Mean Sample Size = 6.000.

FIG.5C

	Groups	N	Subset for alpha = 0.05 1	2	3
Student-Newman-Keuls[a]	S	6	.1667		
	CLE+I/R	6		13.3333	
	CLE+C48/80+I/R	6			17.3333
	I/R	6			
	C48/80+I/R	6			
	Sig.		1.000	1.000	1.000

FIG.5C

Groups	Subset for alpha = 0.05

		4	5
Student-Newman-Keuls[a]	S		
	CLE+I/R		
	CLE+C48/80+I/R		
	I/R	20.1667	
	C48/80+I/R		25.1667
	Sig.	1.000	1.000

Means for groups in homogeneous subsets are displayed.

a. Uses Harmonic Mean Sample Size = 6.000.

FIG.5E

	Groups	N	Subset for alpha = 0.05		
			1	2	3
Student-Newman-Keuls[a]	S	6	438.6667		
	CLE+I/R	6		16047.3167	
	CLE+C48/80+I/R	6		16770.4333	
	I/R	6			18993.9833
	C48/80+I/R	6			
	Sig.		1.000	.277	1.000

FIG.5E

	Groups	Subset for alpha = 0.05
		4
Student-Newman-Keuls[a]	S	
	CLE+I/R	
	CLE+C48/80+I/R	
	I/R	
	C48/80+I/R	25511.1667
	Sig.	1.000

Means for groups in homogeneous subsets are displayed.

a. Uses Harmonic Mean Sample Size = 6.000.

FIG.5F

	Groups	N	Subset for alpha = 0.05		
			1	2	3
Student-Newman-Keuls[a]	S	6	6.7333		
	CLE+I/R	6		10.3833	

	Groups	N			
	CLE+C48/80+I/R	6			12.6750
	I/R	6			
	C48/80+I/R	6			
	Sig.		1.000	1.000	1.000

FIG.5F

	Groups	Subset for alpha = 0.05	
		4	5
Student-Newman-Keuls[a]	S		
	CLE+I/R		
	CLE+C48/80+I/R		
	I/R	17.7167	
	C48/80+I/R		29.0000
	Sig.	1.000	1.000

Means for groups in homogeneous subsets are displayed.

a. Uses Harmonic Mean Sample Size = 6.000.
