# Supplementary material for: Clemastine Fumarate Attenuates Myocardial Ischemia Reperfusion Injury Through Inhibition of Mast Cell Degranulation
Source: Front Pharmacol. 2021 Aug 27;12:704852. doi: 10.3389/fphar.2021.704852 (PMC8430029; doi:10.3389/fphar.2021.704852)
Supplement: Supplementary file 1 [file DataSheet1.ZIP › supplementary/Data Analysis/Figure 6A.pdf]

Oneway

Descriptives

		N	Mean	Std. Deviation	Std. Error	95% Confidence Interval for Mean
						Lower Bound
TPS1h	C	3	.072467	.0013650	.0007881	.069076
	C48/80	3	.077433	.0002309	.0001333	.076860
	CLE1+C48/80	3	.076900	.0001732	.0001000	.076470
	CLE2+C48/80	3	.079233	.0005033	.0002906	.077983
	CLE3+C48/80	3	.073667	.0006506	.0003756	.072050
	CLE4+C48/80	3	.075333	.0005774	.0003333	.073899
	CLE5+C48/80	3	.082033	.0035119	.0020276	.073309
	Total	21	.076724	.0033456	.0007301	.075201
TPS2h	C	3	.081100	.0001732	.0001000	.080670
	C48/80	3	.096333	.0006506	.0003756	.094717
	CLE1+C48/80	3	.086023	.0005600	.0003233	.084632
	CLE2+C48/80	3	.084900	.0007211	.0004163	.083109
	CLE3+C48/80	3	.081467	.0004041	.0002333	.080463
	CLE4+C48/80	3	.080767	.0006807	.0003930	.079076
	CLE5+C48/80	3	.094133	.0026764	.0015452	.087485
	Total	21	.086389	.0061369	.0013392	.083596
TPS4h	C	3	.075333	.0003512	.0002028	.074461
	C48/80	3	.086200	.0010149	.0005859	.083679
	CLE1+C48/80	3	.081333	.0011547	.0006667	.078465
	CLE2+C48/80	3	.082100	.0001732	.0001000	.081670
	CLE3+C48/80	3	.084533	.0006807	.0003930	.082842
	CLE4+C48/80	3	.082100	.0003464	.0002000	.081239
	CLE5+C48/80	3	.085333	.0020817	.0012019	.080162
	Total	21	.082419	.0035455	.0007737	.080805
TPS6h	C	3	.076667	.0003512	.0002028	.075794
	C48/80	3	.083100	.0028844	.0016653	.075935
	CLE1+C48/80	3	.079433	.0002309	.0001333	.078860
	CLE2+C48/80	3	.081467	.0004041	.0002333	.080463
	CLE3+C48/80	3	.082900	.0007211	.0004163	.081109
	CLE4+C48/80	3	.087567	.0005132	.0002963	.086292
	CLE5+C48/80	3	.082100	.0018248	.0010536	.077567
	Total	21	.081890	.0033934	.0007405	.080346

Descriptives

		95% Confidence Interval for Mean		
		Upper Bound	Minimum	Maximum
TPS1h	C	.075858	.0710	.0737
	C48/80	.078007	.0773	.0777
	CLE1+C48/80	.077330	.0767	.0770
	CLE2+C48/80	.080484	.0787	.0797
	CLE3+C48/80	.075283	.0730	.0743
	CLE4+C48/80	.076768	.0750	.0760
	CLE5+C48/80	.090757	.0787	.0857
	Total	.078247	.0710	.0857
TPS2h	C	.081530	.0810	.0813
	C48/80	.097950	.0957	.0970
	CLE1+C48/80	.087415	.0857	.0867
	CLE2+C48/80	.086691	.0843	.0857
	CLE3+C48/80	.082471	.0810	.0817
	CLE4+C48/80	.082458	.0800	.0813
	CLE5+C48/80	.100782	.0917	.0970
	Total	.089183	.0800	.0970
TPS4h	C	.076206	.0750	.0757
	C48/80	.088721	.0853	.0873
	CLE1+C48/80	.084202	.0800	.0820
	CLE2+C48/80	.082530	.0820	.0823
	CLE3+C48/80	.086224	.0840	.0853
	CLE4+C48/80	.082961	.0817	.0823
	CLE5+C48/80	.090504	.0830	.0870
	Total	.084033	.0750	.0873
TPS6h	C	.077539	.0763	.0770
	C48/80	.090265	.0807	.0863
	CLE1+C48/80	.080007	.0793	.0797
	CLE2+C48/80	.082471	.0810	.0817
	CLE3+C48/80	.084691	.0823	.0837
	CLE4+C48/80	.088841	.0870	.0880
	CLE5+C48/80	.086633	.0800	.0833
	Total	.083435	.0763	.0880

ANOVA

		Sum of Squares	df	Mean Square	F	Sig.
TPS1h	Between Groups	.000	6	.000	14.748	.000

	Within Groups	.000	14	.000		
	Total	.000	20			
TPS2h	Between Groups	.001	6	.000	94.481	.000
	Within Groups	.000	14	.000		
	Total	.001	20			
TPS4h	Between Groups	.000	6	.000	37.126	.000
	Within Groups	.000	14	.000		
	Total	.000	20			
TPS6h	Between Groups	.000	6	.000	18.701	.000
	Within Groups	.000	14	.000		
	Total	.000	20			

Post Hoc Tests

Multiple Comparisons

Dependent Variable		(I) Groups	(J) Groups	Mean Difference (I-J)	Std. Error	Sig.	95% Confidence Interval	
							Lower Bound	Upper Bound
TPS1h	LSD	C	C48/80	-.0049667*	.0012067	.001	-.007555	-.002378
			CLE1+C48/80	-.0044333*	.0012067	.003	-.007022	-.001845
			CLE2+C48/80	-.0067667*	.0012067	.000	-.009355	-.004178
			CLE3+C48/80	-.0012000	.0012067	.337	-.003788	.001388
			CLE4+C48/80	-.0028667*	.0012067	.032	-.005455	-.000278
			CLE5+C48/80	-.0095667*	.0012067	.000	-.012155	-.006978
		C48/80	C	.0049667*	.0012067	.001	.002378	.007555
			CLE1+C48/80	.0005333	.0012067	.665	-.002055	.003122
			CLE2+C48/80	-.0018000	.0012067	.158	-.004388	.000788
			CLE3+C48/80	.0037667*	.0012067	.008	.001178	.006355
			CLE4+C48/80	.0021000	.0012067	.104	-.000488	.004688
			CLE5+C48/80	-.0046000*	.0012067	.002	-.007188	-.002012
		CLE1+C48/80	C	.0044333*	.0012067	.003	.001845	.007022
			C48/80	-.0005333	.0012067	.665	-.003122	.002055
			CLE2+C48/80	-.0023333	.0012067	.074	-.004922	.000255
			CLE3+C48/80	.0032333*	.0012067	.018	.000645	.005822
			CLE4+C48/80	.0015667	.0012067	.215	-.001022	.004155
			CLE5+C48/80	-.0051333*	.0012067	.001	-.007722	-.002545

		CLE2+C48/80	C	.0067667*	.0012067	.000	.004178	.009355
			C48/80	.0018000	.0012067	.158	-.000788	.004388
			CLE1+C48/80	.0023333	.0012067	.074	-.000255	.004922
			CLE3+C48/80	.0055667*	.0012067	.000	.002978	.008155
			CLE4+C48/80	.0039000*	.0012067	.006	.001312	.006488
			CLE5+C48/80	-.0028000*	.0012067	.036	-.005388	-.000212
		CLE3+C48/80	C	.0012000	.0012067	.337	-.001388	.003788
			C48/80	-.0037667*	.0012067	.008	-.006355	-.001178
			CLE1+C48/80	-.0032333*	.0012067	.018	-.005822	-.000645
			CLE2+C48/80	-.0055667*	.0012067	.000	-.008155	-.002978
			CLE4+C48/80	-.0016667	.0012067	.189	-.004255	.000922
			CLE5+C48/80	-.0083667*	.0012067	.000	-.010955	-.005778
		CLE4+C48/80	C	.0028667*	.0012067	.032	.000278	.005455
			C48/80	-.0021000	.0012067	.104	-.004688	.000488
			CLE1+C48/80	-.0015667	.0012067	.215	-.004155	.001022
			CLE2+C48/80	-.0039000*	.0012067	.006	-.006488	-.001312
			CLE3+C48/80	.0016667	.0012067	.189	-.000922	.004255
			CLE5+C48/80	-.0067000*	.0012067	.000	-.009288	-.004112
		CLE5+C48/80	C	.0095667*	.0012067	.000	.006978	.012155
			C48/80	.0046000*	.0012067	.002	.002012	.007188
			CLE1+C48/80	.0051333*	.0012067	.001	.002545	.007722
			CLE2+C48/80	.0028000*	.0012067	.036	.000212	.005388
			CLE3+C48/80	.0083667*	.0012067	.000	.005778	.010955
			CLE4+C48/80	.0067000*	.0012067	.000	.004112	.009288
TPS2h	LSD	C	C48/80	-.0152333*	.0009298	.000	-.017227	-.013239
			CLE1+C48/80	-.0049233*	.0009298	.000	-.006917	-.002929
			CLE2+C48/80	-.0038000*	.0009298	.001	-.005794	-.001806
			CLE3+C48/80	-.0003667	.0009298	.699	-.002361	.001627
			CLE4+C48/80	.0003333	.0009298	.725	-.001661	.002327
			CLE5+C48/80	-.0130333*	.0009298	.000	-.015027	-.011039
		C48/80	C	.0152333*	.0009298	.000	.013239	.017227
			CLE1+C48/80	.0103100*	.0009298	.000	.008316	.012304
			CLE2+C48/80	.0114333*	.0009298	.000	.009439	.013427
			CLE3+C48/80	.0148667*	.0009298	.000	.012873	.016861
			CLE4+C48/80	.0155667*	.0009298	.000	.013573	.017561
			CLE5+C48/80	.0022000*	.0009298	.033	.000206	.004194
		CLE1+C48/80	C	.0049233*	.0009298	.000	.002929	.006917
			C48/80	-.0103100*	.0009298	.000	-.012304	-.008316
			CLE2+C48/80	.0011233	.0009298	.247	-.000871	.003117
			CLE3+C48/80	.0045567*	.0009298	.000	.002563	.006551

			CLE4+C48/80	.0052567*	.0009298	.000	.003263	.007251
			CLE5+C48/80	-.0081100*	.0009298	.000	-.010104	-.006116
		CLE2+C48/80	C	.0038000*	.0009298	.001	.001806	.005794
			C48/80	-.0114333*	.0009298	.000	-.013427	-.009439
			CLE1+C48/80	-.0011233	.0009298	.247	-.003117	.000871
			CLE3+C48/80	.0034333*	.0009298	.002	.001439	.005427
			CLE4+C48/80	.0041333*	.0009298	.001	.002139	.006127
			CLE5+C48/80	-.0092333*	.0009298	.000	-.011227	-.007239
		CLE3+C48/80	C	.0003667	.0009298	.699	-.001627	.002361
			C48/80	-.0148667*	.0009298	.000	-.016861	-.012873
			CLE1+C48/80	-.0045567*	.0009298	.000	-.006551	-.002563
			CLE2+C48/80	-.0034333*	.0009298	.002	-.005427	-.001439
			CLE4+C48/80	.0007000	.0009298	.464	-.001294	.002694
			CLE5+C48/80	-.0126667*	.0009298	.000	-.014661	-.010673
		CLE4+C48/80	C	-.0003333	.0009298	.725	-.002327	.001661
			C48/80	-.0155667*	.0009298	.000	-.017561	-.013573
			CLE1+C48/80	-.0052567*	.0009298	.000	-.007251	-.003263
			CLE2+C48/80	-.0041333*	.0009298	.001	-.006127	-.002139
			CLE3+C48/80	-.0007000	.0009298	.464	-.002694	.001294
			CLE5+C48/80	-.0133667*	.0009298	.000	-.015361	-.011373
		CLE5+C48/80	C	.0130333*	.0009298	.000	.011039	.015027
			C48/80	-.0022000*	.0009298	.033	-.004194	-.000206
			CLE1+C48/80	.0081100*	.0009298	.000	.006116	.010104
			CLE2+C48/80	.0092333*	.0009298	.000	.007239	.011227
			CLE3+C48/80	.0126667*	.0009298	.000	.010673	.014661
			CLE4+C48/80	.0133667*	.0009298	.000	.011373	.015361
TPS4h	LSD	C	C48/80	-.0108667*	.0008414	.000	-.012671	-.009062
			CLE1+C48/80	-.0060000*	.0008414	.000	-.007805	-.004195
			CLE2+C48/80	-.0067667*	.0008414	.000	-.008571	-.004962
			CLE3+C48/80	-.0092000*	.0008414	.000	-.011005	-.007395
			CLE4+C48/80	-.0067667*	.0008414	.000	-.008571	-.004962
			CLE5+C48/80	-.0100000*	.0008414	.000	-.011805	-.008195
		C48/80	C	.0108667*	.0008414	.000	.009062	.012671
			CLE1+C48/80	.0048667*	.0008414	.000	.003062	.006671
			CLE2+C48/80	.0041000*	.0008414	.000	.002295	.005905
			CLE3+C48/80	.0016667	.0008414	.068	-.000138	.003471
			CLE4+C48/80	.0041000*	.0008414	.000	.002295	.005905
			CLE5+C48/80	.0008667	.0008414	.320	-.000938	.002671
		CLE1+C48/80	C	.0060000*	.0008414	.000	.004195	.007805
			C48/80	-.0048667*	.0008414	.000	-.006671	-.003062

			CLE2+C48/80	-.0007667	.0008414	.378	-.002571	.001038
			CLE3+C48/80	-.0032000*	.0008414	.002	-.005005	-.001395
			CLE4+C48/80	-.0007667	.0008414	.378	-.002571	.001038
			CLE5+C48/80	-.0040000*	.0008414	.000	-.005805	-.002195
		CLE2+C48/80	C	.0067667*	.0008414	.000	.004962	.008571
			C48/80	-.0041000*	.0008414	.000	-.005905	-.002295
			CLE1+C48/80	.0007667	.0008414	.378	-.001038	.002571
			CLE3+C48/80	-.0024333*	.0008414	.012	-.004238	-.000629
			CLE4+C48/80	.0000000	.0008414	1.000	-.001805	.001805
			CLE5+C48/80	-.0032333*	.0008414	.002	-.005038	-.001429
		CLE3+C48/80	C	.0092000*	.0008414	.000	.007395	.011005
			C48/80	-.0016667	.0008414	.068	-.003471	.000138
			CLE1+C48/80	.0032000*	.0008414	.002	.001395	.005005
			CLE2+C48/80	.0024333*	.0008414	.012	.000629	.004238
			CLE4+C48/80	.0024333*	.0008414	.012	.000629	.004238
			CLE5+C48/80	-.0008000	.0008414	.358	-.002605	.001005
		CLE4+C48/80	C	.0067667*	.0008414	.000	.004962	.008571
			C48/80	-.0041000*	.0008414	.000	-.005905	-.002295
			CLE1+C48/80	.0007667	.0008414	.378	-.001038	.002571
			CLE2+C48/80	.0000000	.0008414	1.000	-.001805	.001805
			CLE3+C48/80	-.0024333*	.0008414	.012	-.004238	-.000629
			CLE5+C48/80	-.0032333*	.0008414	.002	-.005038	-.001429
		CLE5+C48/80	C	.0100000*	.0008414	.000	.008195	.011805
			C48/80	-.0008667	.0008414	.320	-.002671	.000938
			CLE1+C48/80	.0040000*	.0008414	.000	.002195	.005805
			CLE2+C48/80	.0032333*	.0008414	.002	.001429	.005038
			CLE3+C48/80	.0008000	.0008414	.358	-.001005	.002605
			CLE4+C48/80	.0032333*	.0008414	.002	.001429	.005038
TPS6h	LSD	C	C48/80	-.0064333*	.0011030	.000	-.008799	-.004068
			CLE1+C48/80	-.0027667*	.0011030	.025	-.005132	-.000401
			CLE2+C48/80	-.0048000*	.0011030	.001	-.007166	-.002434
			CLE3+C48/80	-.0062333*	.0011030	.000	-.008599	-.003868
			CLE4+C48/80	-.0109000*	.0011030	.000	-.013266	-.008534
			CLE5+C48/80	-.0054333*	.0011030	.000	-.007799	-.003068
		C48/80	C	.0064333*	.0011030	.000	.004068	.008799
			CLE1+C48/80	.0036667*	.0011030	.005	.001301	.006032
			CLE2+C48/80	.0016333	.0011030	.161	-.000732	.003999
			CLE3+C48/80	.0002000	.0011030	.859	-.002166	.002566
			CLE4+C48/80	-.0044667*	.0011030	.001	-.006832	-.002101
			CLE5+C48/80	.0010000	.0011030	.380	-.001366	.003366

	CLE1+C48/80	C	.0027667*	.0011030	.025	.000401	.005132
		C48/80	-.0036667*	.0011030	.005	-.006032	-.001301
		CLE2+C48/80	-.0020333	.0011030	.087	-.004399	.000332
		CLE3+C48/80	-.0034667*	.0011030	.007	-.005832	-.001101
		CLE4+C48/80	-.0081333*	.0011030	.000	-.010499	-.005768
		CLE5+C48/80	-.0026667*	.0011030	.030	-.005032	-.000301
	CLE2+C48/80	C	.0048000*	.0011030	.001	.002434	.007166
		C48/80	-.0016333	.0011030	.161	-.003999	.000732
		CLE1+C48/80	.0020333	.0011030	.087	-.000332	.004399
		CLE3+C48/80	-.0014333	.0011030	.215	-.003799	.000932
		CLE4+C48/80	-.0061000*	.0011030	.000	-.008466	-.003734
		CLE5+C48/80	-.0006333	.0011030	.575	-.002999	.001732
	CLE3+C48/80	C	.0062333*	.0011030	.000	.003868	.008599
		C48/80	-.0002000	.0011030	.859	-.002566	.002166
		CLE1+C48/80	.0034667*	.0011030	.007	.001101	.005832
		CLE2+C48/80	.0014333	.0011030	.215	-.000932	.003799
		CLE4+C48/80	-.0046667*	.0011030	.001	-.007032	-.002301
		CLE5+C48/80	.0008000	.0011030	.480	-.001566	.003166
	CLE4+C48/80	C	.0109000*	.0011030	.000	.008534	.013266
		C48/80	.0044667*	.0011030	.001	.002101	.006832
		CLE1+C48/80	.0081333*	.0011030	.000	.005768	.010499
		CLE2+C48/80	.0061000*	.0011030	.000	.003734	.008466
		CLE3+C48/80	.0046667*	.0011030	.001	.002301	.007032
		CLE5+C48/80	.0054667*	.0011030	.000	.003101	.007832
	CLE5+C48/80	C	.0054333*	.0011030	.000	.003068	.007799
		C48/80	-.0010000	.0011030	.380	-.003366	.001366
		CLE1+C48/80	.0026667*	.0011030	.030	.000301	.005032
		CLE2+C48/80	.0006333	.0011030	.575	-.001732	.002999
		CLE3+C48/80	-.0008000	.0011030	.480	-.003166	.001566
		CLE4+C48/80	-.0054667*	.0011030	.000	-.007832	-.003101

*. The mean difference is significant at the 0.05 level.

Homogeneous Subsets

TPS1h

Groups	N	Subset for alpha = 0.05

			1	2	3	4
Student-Newman-Keuls[a]	C	3	.072467			
	CLE3+C48/80	3	.073667			
	CLE4+C48/80	3	.075333	.075333		
	CLE1+C48/80	3		.076900	.076900	
	C48/80	3		.077433	.077433	
	CLE2+C48/80	3			.079233	
	CLE5+C48/80	3				.082033
	Sig.		.078	.225	.166	1.000

Means for groups in homogeneous subsets are displayed.

a. Uses Harmonic Mean Sample Size = 3.000.

TPS2h

			Subset for alpha = 0.05			
	Groups	N	1	2	3	4
Student-Newman-Keuls[a]	CLE4+C48/80	3	.080767			
	C	3	.081100			
	CLE3+C48/80	3	.081467			
	CLE2+C48/80	3		.084900		
	CLE1+C48/80	3		.086023		
	CLE5+C48/80	3			.094133	
	C48/80	3				.096333
	Sig.		.737	.247	1.000	1.000

Means for groups in homogeneous subsets are displayed.

a. Uses Harmonic Mean Sample Size = 3.000.

TPS4h

			Subset for alpha = 0.05		
	Groups	N	1	2	3
Student-Newman-Keuls[a]	C	3	.075333		
	CLE1+C48/80	3		.081333	
	CLE4+C48/80	3		.082100	
	CLE2+C48/80	3		.082100	
	CLE3+C48/80	3			.084533
	CLE5+C48/80	3			.085333
	C48/80	3			.086200
	Sig.		1.000	.642	.154

Means for groups in homogeneous subsets are displayed.
a. Uses Harmonic Mean Sample Size = 3.000.

TPS6h

	Groups	N	Subset for alpha = 0.05			
			1	2	3	4
Student-Newman-Keuls[a]	C	3	.076667			
	CLE1+C48/80	3		.079433		
	CLE2+C48/80	3		.081467	.081467	
	CLE5+C48/80	3		.082100	.082100	
	CLE3+C48/80	3			.082900	
	C48/80	3			.083100	
	CLE4+C48/80	3				.087567
	Sig.		1.000	.072	.474	1.000

Means for groups in homogeneous subsets are displayed.
a. Uses Harmonic Mean Sample Size = 3.000.
