# Supplementary material for: Clemastine Fumarate Attenuates Myocardial Ischemia Reperfusion Injury Through Inhibition of Mast Cell Degranulation
Source: Front Pharmacol. 2021 Aug 27;12:704852. doi: 10.3389/fphar.2021.704852 (PMC8430029; doi:10.3389/fphar.2021.704852)
Supplement: Supplementary file 1 [file DataSheet1.ZIP › supplementary/Data Analysis/Figure 6B.pdf]

# Oneway

Descriptives

| | | N | Mean | Std. Deviation | Std. Error | 95% Confidence Interval for Mean |
|---|---|---|---|---|---|---|
| | | | | | | Lower Bound |
| CCK8OD1h | C | 3 | 98.023333 | 2.9195262 | 1.6855892 | 90.770828 |
| | C48/80 | 3 | 41.770000 | 2.4224574 | 1.3986064 | 35.752282 |
| | CLE1+C48/80 | 3 | 44.330000 | 4.8764639 | 2.8154277 | 32.216192 |
| | CLE2+C48/80 | 3 | 44.756667 | 2.1129206 | 1.2198953 | 39.507881 |
| | CLE3+C48/80 | 3 | 44.327056 | 3.3324087 | 1.9239670 | 36.048894 |
| | CLE4+C48/80 | 3 | 45.253407 | 2.6005262 | 1.5014145 | 38.793342 |
| | CLE5+C48/80 | 3 | 22.033379 | 1.0116953 | .5841026 | 19.520189 |
| | Total | 21 | 48.641977 | 22.2437675 | 4.8539880 | 38.516736 |
| CCK8OD2h | C | 3 | 99.6583 | .39142 | .22599 | 98.6860 |
| | C48/80 | 3 | 63.0579 | 2.58028 | 1.48973 | 56.6481 |
| | CLE1+C48/80 | 3 | 71.7702 | .35282 | .20370 | 70.8938 |
| | CLE2+C48/80 | 3 | 70.4463 | 2.48880 | 1.43691 | 64.2638 |
| | CLE3+C48/80 | 3 | 80.9951 | .38614 | .22294 | 80.0359 |
| | CLE4+C48/80 | 3 | 83.1732 | 1.54591 | .89253 | 79.3329 |
| | CLE5+C48/80 | 3 | 9.9722 | 2.92189 | 1.68695 | 2.7139 |
| | Total | 21 | 68.4390 | 26.91091 | 5.87244 | 56.1893 |
| CCK8OD4h | C | 3 | 99.4687 | .46855 | .27052 | 98.3048 |
| | C48/80 | 3 | 76.4266 | 2.16522 | 1.25009 | 71.0479 |
| | CLE1+C48/80 | 3 | 85.4782 | 1.97470 | 1.14009 | 80.5727 |
| | CLE2+C48/80 | 3 | 70.1495 | 1.30717 | .75470 | 66.9024 |
| | CLE3+C48/80 | 3 | 77.4301 | 3.09134 | 1.78479 | 69.7508 |
| | CLE4+C48/80 | 3 | 79.9882 | 1.43266 | .82715 | 76.4293 |
| | CLE5+C48/80 | 3 | 3.3909 | 2.35930 | 1.36214 | -2.4700 |
| | Total | 21 | 70.3332 | 29.39065 | 6.41357 | 56.9547 |
| CCK8OD6h | C | 3 | 99.5437 | .64201 | .37067 | 97.9488 |
| | C48/80 | 3 | 75.6944 | 1.07198 | .61891 | 73.0315 |
| | CLE1+C48/80 | 3 | 84.5635 | .34366 | .19841 | 83.7098 |
| | CLE2+C48/80 | 3 | 74.6627 | 2.16042 | 1.24732 | 69.2959 |
| | CLE3+C48/80 | 3 | 80.2381 | 1.10240 | .63647 | 77.4996 |
| | CLE4+C48/80 | 3 | 75.1984 | 1.61081 | .93000 | 71.1969 |
| | CLE5+C48/80 | 3 | 5.8333 | 2.91397 | 1.68238 | -1.4054 |
| | Total | 21 | 70.8192 | 28.45003 | 6.20831 | 57.8689 |

Descriptives

| | | 95% Confidence Interval for Mean Upper Bound | Minimum | Maximum |
|---|---|---|---|---|
| CCK8OD1h | C | 105.275839 | 94.6700 | 100.0000 |
| | C48/80 | 47.787718 | 39.1400 | 43.9100 |
| | CLE1+C48/80 | 56.443808 | 38.7000 | 47.2300 |
| | CLE2+C48/80 | 50.005452 | 42.9500 | 47.0800 |
| | CLE3+C48/80 | 52.605218 | 40.6063 | 47.0372 |
| | CLE4+C48/80 | 51.713472 | 43.4313 | 48.2315 |
| | CLE5+C48/80 | 24.546570 | 21.2448 | 23.1741 |
| | Total | 58.767219 | 21.2448 | 100.0000 |
| CCK8OD2h | C | 100.6307 | 99.23 | 100.00 |
| | C48/80 | 69.4676 | 60.15 | 65.09 |
| | CLE1+C48/80 | 72.6467 | 71.36 | 72.01 |
| | CLE2+C48/80 | 76.6288 | 67.71 | 72.58 |
| | CLE3+C48/80 | 81.9543 | 80.59 | 81.36 |
| | CLE4+C48/80 | 87.0134 | 81.87 | 84.88 |
| | CLE5+C48/80 | 17.2306 | 7.17 | 13.00 |
| | Total | 80.6887 | 7.17 | 100.00 |
| CCK8OD4h | C | 100.6327 | 99.11 | 100.00 |
| | C48/80 | 81.8053 | 74.73 | 78.87 |
| | CLE1+C48/80 | 90.3836 | 84.00 | 87.72 |
| | CLE2+C48/80 | 73.3967 | 68.65 | 71.07 |
| | CLE3+C48/80 | 85.1095 | 75.44 | 80.99 |
| | CLE4+C48/80 | 83.5471 | 78.81 | 81.58 |
| | CLE5+C48/80 | 9.2517 | 1.06 | 5.78 |
| | Total | 83.7116 | 1.06 | 100.00 |
| CCK8OD6h | C | 101.1385 | 98.81 | 100.00 |
| | C48/80 | 78.3574 | 74.46 | 76.43 |
| | CLE1+C48/80 | 85.4172 | 84.17 | 84.76 |
| | CLE2+C48/80 | 80.0295 | 72.68 | 76.96 |
| | CLE3+C48/80 | 82.9766 | 79.40 | 81.49 |
| | CLE4+C48/80 | 79.1999 | 73.39 | 76.49 |
| | CLE5+C48/80 | 13.0720 | 3.13 | 8.92 |
| | Total | 83.7695 | 3.13 | 100.00 |

## ANOVA

| | Sum of Squares | df | Mean Square | F | Sig. |
|---|---|---|---|---|---|

| | | | | | | |
|---|---|---|---|---|---|---|
| CCK8OD1h | Between Groups | 9772.649 | 6 | 1628.775 | 185.306 | .000 |
| | Within Groups | 123.055 | 14 | 8.790 | | |
| | Total | 9895.704 | 20 | | | |
| CCK8OD2h | Between Groups | 14435.530 | 6 | 2405.922 | 695.755 | .000 |
| | Within Groups | 48.412 | 14 | 3.458 | | |
| | Total | 14483.942 | 20 | | | |
| CCK8OD4h | Between Groups | 17220.822 | 6 | 2870.137 | 725.539 | .000 |
| | Within Groups | 55.382 | 14 | 3.956 | | |
| | Total | 17276.204 | 20 | | | |
| CCK8OD6h | Between Groups | 16150.788 | 6 | 2691.798 | 1010.431 | .000 |
| | Within Groups | 37.296 | 14 | 2.664 | | |
| | Total | 16188.084 | 20 | | | |

# Post Hoc Tests

## Multiple Comparisons

| Dependent Variable | | (I) Groups | (J) Groups | Mean Difference (I-J) | Std. Error | Sig. | 95% Confidence Interval Lower Bound | 95% Confidence Interval Upper Bound |
|---|---|---|---|---|---|---|---|---|
| CCK8OD1h | LSD | C | C48/80 | 56.2533333* | 2.4206941 | .000 | 51.061461 | 61.445206 |
| | | | CLE1+C48/80 | 53.6933333* | 2.4206941 | .000 | 48.501461 | 58.885206 |
| | | | CLE2+C48/80 | 53.2666667* | 2.4206941 | .000 | 48.074794 | 58.458539 |
| | | | CLE3+C48/80 | 53.6962777* | 2.4206941 | .000 | 48.504405 | 58.888150 |
| | | | CLE4+C48/80 | 52.7699265* | 2.4206941 | .000 | 47.578054 | 57.961799 |
| | | | CLE5+C48/80 | 75.9899541* | 2.4206941 | .000 | 70.798082 | 81.181827 |
| | | C48/80 | C | -56.2533333* | 2.4206941 | .000 | -61.445206 | -51.061461 |
| | | | CLE1+C48/80 | -2.5600000 | 2.4206941 | .308 | -7.751872 | 2.631872 |
| | | | CLE2+C48/80 | -2.9866667 | 2.4206941 | .238 | -8.178539 | 2.205206 |

| | | | | | | | |
|---|---|---|---|---|---|---|---|
| | | CLE3+C48/80 | -2.5570556 | 2.4206941 | .309 | -7.748928 | 2.634817 |
| | | CLE4+C48/80 | -3.4834068 | 2.4206941 | .172 | -8.675279 | 1.708466 |
| | | CLE5+C48/80 | 19.7366207* | 2.4206941 | .000 | 14.544748 | 24.928493 |
| | CLE1+C48/80 | C | -53.6933333* | 2.4206941 | .000 | -58.885206 | -48.501461 |
| | | C48/80 | 2.5600000 | 2.4206941 | .308 | -2.631872 | 7.751872 |
| | | CLE2+C48/80 | -.4266667 | 2.4206941 | .863 | -5.618539 | 4.765206 |
| | | CLE3+C48/80 | .0029444 | 2.4206941 | .999 | -5.188928 | 5.194817 |
| | | CLE4+C48/80 | -.9234068 | 2.4206941 | .709 | -6.115279 | 4.268466 |
| | | CLE5+C48/80 | 22.2966207* | 2.4206941 | .000 | 17.104748 | 27.488493 |
| | CLE2+C48/80 | C | -53.2666667* | 2.4206941 | .000 | -58.458539 | -48.074794 |
| | | C48/80 | 2.9866667 | 2.4206941 | .238 | -2.205206 | 8.178539 |
| | | CLE1+C48/80 | .4266667 | 2.4206941 | .863 | -4.765206 | 5.618539 |
| | | CLE3+C48/80 | .4296111 | 2.4206941 | .862 | -4.762261 | 5.621484 |
| | | CLE4+C48/80 | -.4967402 | 2.4206941 | .840 | -5.688613 | 4.695132 |
| | | CLE5+C48/80 | 22.7232874* | 2.4206941 | .000 | 17.531415 | 27.915160 |
| | CLE3+C48/80 | C | -53.6962777* | 2.4206941 | .000 | -58.888150 | -48.504405 |
| | | C48/80 | 2.5570556 | 2.4206941 | .309 | -2.634817 | 7.748928 |
| | | CLE1+C48/80 | -.0029444 | 2.4206941 | .999 | -5.194817 | 5.188928 |
| | | CLE2+C48/80 | -.4296111 | 2.4206941 | .862 | -5.621484 | 4.762261 |
| | | CLE4+C48/80 | -.9263512 | 2.4206941 | .708 | -6.118224 | 4.265521 |
| | | CLE5+C48/80 | 22.2936763* | 2.4206941 | .000 | 17.101804 | 27.485549 |

| | | | | | | | | |
|---|---|---|---|---|---|---|---|---|
| | | CLE4+C48/80 | C | -52.7699265* | 2.4206941 | .000 | -57.961799 | -47.578054 |
| | | | C48/80 | 3.4834068 | 2.4206941 | .172 | -1.708466 | 8.675279 |
| | | | CLE1+C48/80 | .9234068 | 2.4206941 | .709 | -4.268466 | 6.115279 |
| | | | CLE2+C48/80 | .4967402 | 2.4206941 | .840 | -4.695132 | 5.688613 |
| | | | CLE3+C48/80 | .9263512 | 2.4206941 | .708 | -4.265521 | 6.118224 |
| | | | CLE5+C48/80 | 23.2200276* | 2.4206941 | .000 | 18.028155 | 28.411900 |
| | | CLE5+C48/80 | C | -75.9899541* | 2.4206941 | .000 | -81.181827 | -70.798082 |
| | | | C48/80 | -19.7366207* | 2.4206941 | .000 | -24.928493 | -14.544748 |
| | | | CLE1+C48/80 | -22.2966207* | 2.4206941 | .000 | -27.488493 | -17.104748 |
| | | | CLE2+C48/80 | -22.7232874* | 2.4206941 | .000 | -27.915160 | -17.531415 |
| | | | CLE3+C48/80 | -22.2936763* | 2.4206941 | .000 | -27.485549 | -17.101804 |
| | | | CLE4+C48/80 | -23.2200276* | 2.4206941 | .000 | -28.411900 | -18.028155 |
| CCK8OD2h | LSD | C | C48/80 | 36.60047* | 1.51833 | .000 | 33.3440 | 39.8570 |
| | | | CLE1+C48/80 | 27.88811* | 1.51833 | .000 | 24.6316 | 31.1446 |
| | | | CLE2+C48/80 | 29.21204* | 1.51833 | .000 | 25.9555 | 32.4685 |
| | | | CLE3+C48/80 | 18.66325* | 1.51833 | .000 | 15.4067 | 21.9198 |
| | | | CLE4+C48/80 | 16.48516* | 1.51833 | .000 | 13.2287 | 19.7417 |
| | | | CLE5+C48/80 | 89.68610* | 1.51833 | .000 | 86.4296 | 92.9426 |
| | | C48/80 | C | -36.60047* | 1.51833 | .000 | -39.8570 | -33.3440 |
| | | | CLE1+C48/80 | -8.71236* | 1.51833 | .000 | -11.9689 | -5.4559 |
| | | | CLE2+C48/80 | -7.38843* | 1.51833 | .000 | -10.6449 | -4.1319 |
| | | | CLE3+C48/80 | -17.93722* | 1.51833 | .000 | -21.1937 | -14.6807 |
| | | | CLE4+C48/80 | -20.11531* | 1.51833 | .000 | -23.3718 | -16.8588 |
| | | | CLE5+C48/80 | 53.08563* | 1.51833 | .000 | 49.8291 | 56.3421 |
| | | CLE1+C48/80 | C | -27.88811* | 1.51833 | .000 | -31.1446 | -24.6316 |
| | | | C48/80 | 8.71236* | 1.51833 | .000 | 5.4559 | 11.9689 |
| | | | CLE2+C48/80 | 1.32394 | 1.51833 | .398 | -1.9326 | 4.5804 |
| | | | CLE3+C48/80 | -9.22486* | 1.51833 | .000 | -12.4814 | -5.9684 |
| | | | CLE4+C48/80 | -11.40295* | 1.51833 | .000 | -14.6594 | -8.1464 |

| | | | | | | | | |
|---|---|---|---|---|---|---|---|---|
| | | | CLE5+C48/80 | 61.79799* | 1.51833 | .000 | 58.5415 | 65.0545 |
| | | CLE2+C48/80 | C | -29.21204* | 1.51833 | .000 | -32.4685 | -25.9555 |
| | | | C48/80 | 7.38843* | 1.51833 | .000 | 4.1319 | 10.6449 |
| | | | CLE1+C48/80 | -1.32394 | 1.51833 | .398 | -4.5804 | 1.9326 |
| | | | CLE3+C48/80 | -10.54879* | 1.51833 | .000 | -13.8053 | -7.2923 |
| | | | CLE4+C48/80 | -12.72688* | 1.51833 | .000 | -15.9834 | -9.4704 |
| | | | CLE5+C48/80 | 60.47406* | 1.51833 | .000 | 57.2176 | 63.7306 |
| | | CLE3+C48/80 | C | -18.66325* | 1.51833 | .000 | -21.9198 | -15.4067 |
| | | | C48/80 | 17.93722* | 1.51833 | .000 | 14.6807 | 21.1937 |
| | | | CLE1+C48/80 | 9.22486* | 1.51833 | .000 | 5.9684 | 12.4814 |
| | | | CLE2+C48/80 | 10.54879* | 1.51833 | .000 | 7.2923 | 13.8053 |
| | | | CLE4+C48/80 | -2.17809 | 1.51833 | .173 | -5.4346 | 1.0784 |
| | | | CLE5+C48/80 | 71.02285* | 1.51833 | .000 | 67.7663 | 74.2793 |
| | | CLE4+C48/80 | C | -16.48516* | 1.51833 | .000 | -19.7417 | -13.2287 |
| | | | C48/80 | 20.11531* | 1.51833 | .000 | 16.8588 | 23.3718 |
| | | | CLE1+C48/80 | 11.40295* | 1.51833 | .000 | 8.1464 | 14.6594 |
| | | | CLE2+C48/80 | 12.72688* | 1.51833 | .000 | 9.4704 | 15.9834 |
| | | | CLE3+C48/80 | 2.17809 | 1.51833 | .173 | -1.0784 | 5.4346 |
| | | | CLE5+C48/80 | 73.20094* | 1.51833 | .000 | 69.9444 | 76.4574 |
| | | CLE5+C48/80 | C | -89.68610* | 1.51833 | .000 | -92.9426 | -86.4296 |
| | | | C48/80 | -53.08563* | 1.51833 | .000 | -56.3421 | -49.8291 |
| | | | CLE1+C48/80 | -61.79799* | 1.51833 | .000 | -65.0545 | -58.5415 |
| | | | CLE2+C48/80 | -60.47406* | 1.51833 | .000 | -63.7306 | -57.2176 |
| | | | CLE3+C48/80 | -71.02285* | 1.51833 | .000 | -74.2793 | -67.7663 |
| | | | CLE4+C48/80 | -73.20094* | 1.51833 | .000 | -76.4574 | -69.9444 |
| CCK8OD4h | LSD | C | C48/80 | 23.04211* | 1.62396 | .000 | 19.5591 | 26.5252 |
| | | | CLE1+C48/80 | 13.99055* | 1.62396 | .000 | 10.5075 | 17.4736 |
| | | | CLE2+C48/80 | 29.31917* | 1.62396 | .000 | 25.8361 | 32.8022 |
| | | | CLE3+C48/80 | 22.03857* | 1.62396 | .000 | 18.5555 | 25.5216 |
| | | | CLE4+C48/80 | 19.48052* | 1.62396 | .000 | 15.9975 | 22.9636 |
| | | | CLE5+C48/80 | 96.07786* | 1.62396 | .000 | 92.5948 | 99.5609 |
| | | C48/80 | C | -23.04211* | 1.62396 | .000 | -26.5252 | -19.5591 |
| | | | CLE1+C48/80 | -9.05155* | 1.62396 | .000 | -12.5346 | -5.5685 |
| | | | CLE2+C48/80 | 6.27706* | 1.62396 | .002 | 2.7940 | 9.7601 |
| | | | CLE3+C48/80 | -1.00354 | 1.62396 | .547 | -4.4866 | 2.4795 |
| | | | CLE4+C48/80 | -3.56159* | 1.62396 | .046 | -7.0446 | -.0785 |
| | | | CLE5+C48/80 | 73.03575* | 1.62396 | .000 | 69.5527 | 76.5188 |
| | | CLE1+C48/80 | C | -13.99055* | 1.62396 | .000 | -17.4736 | -10.5075 |
| | | | C48/80 | 9.05155* | 1.62396 | .000 | 5.5685 | 12.5346 |
| | | | CLE2+C48/80 | 15.32861* | 1.62396 | .000 | 11.8456 | 18.8117 |

| | | | | | | | |
|---|---|---|---|---|---|---|---|
| | | | CLE3+C48/80 | 8.04801* | 1.62396 | .000 | 4.5650 | 11.5311 |
| | | | CLE4+C48/80 | 5.48996* | 1.62396 | .004 | 2.0069 | 8.9730 |
| | | | CLE5+C48/80 | 82.08730* | 1.62396 | .000 | 78.6043 | 85.5703 |
| | | CLE2+C48/80 | C | -29.31917* | 1.62396 | .000 | -32.8022 | -25.8361 |
| | | | C48/80 | -6.27706* | 1.62396 | .002 | -9.7601 | -2.7940 |
| | | | CLE1+C48/80 | -15.32861* | 1.62396 | .000 | -18.8117 | -11.8456 |
| | | | CLE3+C48/80 | -7.28060* | 1.62396 | .001 | -10.7636 | -3.7976 |
| | | | CLE4+C48/80 | -9.83865* | 1.62396 | .000 | -13.3217 | -6.3556 |
| | | | CLE5+C48/80 | 66.75869* | 1.62396 | .000 | 63.2756 | 70.2417 |
| | | CLE3+C48/80 | C | -22.03857* | 1.62396 | .000 | -25.5216 | -18.5555 |
| | | | C48/80 | 1.00354 | 1.62396 | .547 | -2.4795 | 4.4866 |
| | | | CLE1+C48/80 | -8.04801* | 1.62396 | .000 | -11.5311 | -4.5650 |
| | | | CLE2+C48/80 | 7.28060* | 1.62396 | .001 | 3.7976 | 10.7636 |
| | | | CLE4+C48/80 | -2.55805 | 1.62396 | .138 | -6.0411 | .9250 |
| | | | CLE5+C48/80 | 74.03929* | 1.62396 | .000 | 70.5562 | 77.5223 |
| | | CLE4+C48/80 | C | -19.48052* | 1.62396 | .000 | -22.9636 | -15.9975 |
| | | | C48/80 | 3.56159* | 1.62396 | .046 | .0785 | 7.0446 |
| | | | CLE1+C48/80 | -5.48996* | 1.62396 | .004 | -8.9730 | -2.0069 |
| | | | CLE2+C48/80 | 9.83865* | 1.62396 | .000 | 6.3556 | 13.3217 |
| | | | CLE3+C48/80 | 2.55805 | 1.62396 | .138 | -.9250 | 6.0411 |
| | | | CLE5+C48/80 | 76.59734* | 1.62396 | .000 | 73.1143 | 80.0804 |
| | | CLE5+C48/80 | C | -96.07786* | 1.62396 | .000 | -99.5609 | -92.5948 |
| | | | C48/80 | -73.03575* | 1.62396 | .000 | -76.5188 | -69.5527 |
| | | | CLE1+C48/80 | -82.08730* | 1.62396 | .000 | -85.5703 | -78.6043 |
| | | | CLE2+C48/80 | -66.75869* | 1.62396 | .000 | -70.2417 | -63.2756 |
| | | | CLE3+C48/80 | -74.03929* | 1.62396 | .000 | -77.5223 | -70.5562 |
| | | | CLE4+C48/80 | -76.59734* | 1.62396 | .000 | -80.0804 | -73.1143 |
| CCK8OD6h | LSD | C | C48/80 | 23.84921* | 1.33267 | .000 | 20.9909 | 26.7075 |
| | | | CLE1+C48/80 | 14.98016* | 1.33267 | .000 | 12.1219 | 17.8384 |
| | | | CLE2+C48/80 | 24.88095* | 1.33267 | .000 | 22.0227 | 27.7392 |
| | | | CLE3+C48/80 | 19.30556* | 1.33267 | .000 | 16.4473 | 22.1638 |
| | | | CLE4+C48/80 | 24.34524* | 1.33267 | .000 | 21.4869 | 27.2035 |
| | | | CLE5+C48/80 | 93.71032* | 1.33267 | .000 | 90.8520 | 96.5686 |
| | | C48/80 | C | -23.84921* | 1.33267 | .000 | -26.7075 | -20.9909 |
| | | | CLE1+C48/80 | -8.86905* | 1.33267 | .000 | -11.7273 | -6.0108 |
| | | | CLE2+C48/80 | 1.03175 | 1.33267 | .452 | -1.8265 | 3.8900 |
| | | | CLE3+C48/80 | -4.54365* | 1.33267 | .004 | -7.4019 | -1.6854 |
| | | | CLE4+C48/80 | .49603 | 1.33267 | .715 | -2.3623 | 3.3543 |
| | | | CLE5+C48/80 | 69.86111* | 1.33267 | .000 | 67.0028 | 72.7194 |
| | | CLE1+C48/80 | C | -14.98016* | 1.33267 | .000 | -17.8384 | -12.1219 |

| | | | | | | |
|---|---|---|---|---|---|---|
| | C48/80 | 8.86905* | 1.33267 | .000 | 6.0108 | 11.7273 |
| | CLE2+C48/80 | 9.90079* | 1.33267 | .000 | 7.0425 | 12.7591 |
| | CLE3+C48/80 | 4.32540* | 1.33267 | .006 | 1.4671 | 7.1837 |
| | CLE4+C48/80 | 9.36508* | 1.33267 | .000 | 6.5068 | 12.2234 |
| | CLE5+C48/80 | 78.73016* | 1.33267 | .000 | 75.8719 | 81.5884 |
| CLE2+C48/80 | C | -24.88095* | 1.33267 | .000 | -27.7392 | -22.0227 |
| | C48/80 | -1.03175 | 1.33267 | .452 | -3.8900 | 1.8265 |
| | CLE1+C48/80 | -9.90079* | 1.33267 | .000 | -12.7591 | -7.0425 |
| | CLE3+C48/80 | -5.57540* | 1.33267 | .001 | -8.4337 | -2.7171 |
| | CLE4+C48/80 | -.53571 | 1.33267 | .694 | -3.3940 | 2.3226 |
| | CLE5+C48/80 | 68.82937* | 1.33267 | .000 | 65.9711 | 71.6877 |
| CLE3+C48/80 | C | -19.30556* | 1.33267 | .000 | -22.1638 | -16.4473 |
| | C48/80 | 4.54365* | 1.33267 | .004 | 1.6854 | 7.4019 |
| | CLE1+C48/80 | -4.32540* | 1.33267 | .006 | -7.1837 | -1.4671 |
| | CLE2+C48/80 | 5.57540* | 1.33267 | .001 | 2.7171 | 8.4337 |
| | CLE4+C48/80 | 5.03968* | 1.33267 | .002 | 2.1814 | 7.8980 |
| | CLE5+C48/80 | 74.40476* | 1.33267 | .000 | 71.5465 | 77.2631 |
| CLE4+C48/80 | C | -24.34524* | 1.33267 | .000 | -27.2035 | -21.4869 |
| | C48/80 | -.49603 | 1.33267 | .715 | -3.3543 | 2.3623 |
| | CLE1+C48/80 | -9.36508* | 1.33267 | .000 | -12.2234 | -6.5068 |
| | CLE2+C48/80 | .53571 | 1.33267 | .694 | -2.3226 | 3.3940 |
| | CLE3+C48/80 | -5.03968* | 1.33267 | .002 | -7.8980 | -2.1814 |
| | CLE5+C48/80 | 69.36508* | 1.33267 | .000 | 66.5068 | 72.2234 |
| CLE5+C48/80 | C | -93.71032* | 1.33267 | .000 | -96.5686 | -90.8520 |
| | C48/80 | -69.86111* | 1.33267 | .000 | -72.7194 | -67.0028 |
| | CLE1+C48/80 | -78.73016* | 1.33267 | .000 | -81.5884 | -75.8719 |
| | CLE2+C48/80 | -68.82937* | 1.33267 | .000 | -71.6877 | -65.9711 |
| | CLE3+C48/80 | -74.40476* | 1.33267 | .000 | -77.2631 | -71.5465 |
| | CLE4+C48/80 | -69.36508* | 1.33267 | .000 | -72.2234 | -66.5068 |

*. The mean difference is significant at the 0.05 level.

Homogeneous Subsets

**CCK8OD1h**

| Groups | N | Subset for alpha = 0.05 | | |
|---|---|---|---|---|
| | | 1 | 2 | 3 |

| | | | | | |
|---|---|---|---|---|---|
| Student-Newman-Keuls[a] | CLE5+C48/80 | 3 | 22.033379 | | |
| | C48/80 | 3 | | 41.770000 | |
| | CLE3+C48/80 | 3 | | 44.327056 | |
| | CLE1+C48/80 | 3 | | 44.330000 | |
| | CLE2+C48/80 | 3 | | 44.756667 | |
| | CLE4+C48/80 | 3 | | 45.253407 | |
| | C | 3 | | | 98.023333 |
| | Sig. | | 1.000 | .615 | 1.000 |

Means for groups in homogeneous subsets are displayed.

a. Uses Harmonic Mean Sample Size = 3.000.

### CCK8OD2h

| | Groups | N | Subset for alpha = 0.05 1 | 2 | 3 | 4 |
|---|---|---|---|---|---|---|
| Student-Newman-Keuls[a] | CLE5+C48/80 | 3 | 9.9722 | | | |
| | C48/80 | 3 | | 63.0579 | | |
| | CLE2+C48/80 | 3 | | | 70.4463 | |
| | CLE1+C48/80 | 3 | | | 71.7702 | |
| | CLE3+C48/80 | 3 | | | | 80.9951 |
| | CLE4+C48/80 | 3 | | | | 83.1732 |
| | C | 3 | | | | |
| | Sig. | | 1.000 | 1.000 | .398 | .173 |

### CCK8OD2h

| | Groups | Subset for alpha = 0.05 5 |
|---|---|---|
| Student-Newman-Keuls[a] | CLE5+C48/80 | |
| | C48/80 | |
| | CLE2+C48/80 | |
| | CLE1+C48/80 | |
| | CLE3+C48/80 | |
| | CLE4+C48/80 | |
| | C | 99.6583 |
| | Sig. | 1.000 |

Means for groups in homogeneous subsets are displayed.

a. Uses Harmonic Mean Sample Size = 3.000.

## CCK8OD4h

| | Groups | N | Subset for alpha = 0.05 | | | |
|---|---|---|---|---|---|---|
| | | | 1 | 2 | 3 | 4 |
| Student-Newman-Keuls[a] | CLE5+C48/80 | 3 | 3.3909 | | | |
| | CLE2+C48/80 | 3 | | 70.1495 | | |
| | C48/80 | 3 | | | 76.4266 | |
| | CLE3+C48/80 | 3 | | | 77.4301 | |
| | CLE4+C48/80 | 3 | | | 79.9882 | |
| | CLE1+C48/80 | 3 | | | | 85.4782 |
| | C | 3 | | | | |
| | Sig. | | 1.000 | 1.000 | .107 | 1.000 |

## CCK8OD4h

| | Groups | Subset for alpha = 0.05 |
|---|---|---|
| | | 5 |
| Student-Newman-Keuls[a] | CLE5+C48/80 | |
| | CLE2+C48/80 | |
| | C48/80 | |
| | CLE3+C48/80 | |
| | CLE4+C48/80 | |
| | CLE1+C48/80 | |
| | C | 99.4687 |
| | Sig. | 1.000 |

Means for groups in homogeneous subsets are displayed.

a. Uses Harmonic Mean Sample Size = 3.000.

## CCK8OD6h

| | Groups | N | Subset for alpha = 0.05 | | | |
|---|---|---|---|---|---|---|
| | | | 1 | 2 | 3 | 4 |
| Student-Newman-Keuls[a] | CLE5+C48/80 | 3 | 5.8333 | | | |
| | CLE2+C48/80 | 3 | | 74.6627 | | |
| | CLE4+C48/80 | 3 | | 75.1984 | | |
| | C48/80 | 3 | | 75.6944 | | |
| | CLE3+C48/80 | 3 | | | 80.2381 | |
| | CLE1+C48/80 | 3 | | | | 84.5635 |
| | C | 3 | | | | |
| | Sig. | | 1.000 | .724 | 1.000 | 1.000 |

## CCK8OD6h

| | Groups | Subset for alpha = 0.05 |
|---|---|---|
| | | 5 |
| Student-Newman-Keuls[a] | CLE5+C48/80 | |
| | CLE2+C48/80 | |
| | CLE4+C48/80 | |
| | C48/80 | |
| | CLE3+C48/80 | |
| | CLE1+C48/80 | |
| | C | 99.5437 |
| | Sig. | 1.000 |

Means for groups in homogeneous subsets are displayed.

a. Uses Harmonic Mean Sample Size = 3.000.
