# Supplementary material for: Clemastine Fumarate Attenuates Myocardial Ischemia Reperfusion Injury Through Inhibition of Mast Cell Degranulation
Source: Front Pharmacol. 2021 Aug 27;12:704852. doi: 10.3389/fphar.2021.704852 (PMC8430029; doi:10.3389/fphar.2021.704852)
Supplement: Supplementary file 1 [file DataSheet1.ZIP › supplementary/Data Analysis/Figure 6C.pdf]

Oneway

Descriptives

CCK8OD

	N	Mean	Std. Deviation	Std. Error	95% Confidence Interval for Mean	
					Lower Bound	Upper Bound
C	3	94.9250	1.59820	.92272	90.9549	98.8952
CLE1	3	95.6171	2.79686	1.61477	88.6693	102.5648
CLE2	3	94.4637	5.91278	3.41375	79.7755	109.1518
CLE3	3	96.5398	3.46021	1.99775	87.9442	105.1354
CLE4	3	93.5409	5.80380	3.35083	79.1235	107.9584
CLE5	3	2.6528	.39955	.23068	1.6603	3.6454
Total	18	79.6232	35.58405	8.38724	61.9277	97.3187

Descriptives

CCK8OD

	Minimum	Maximum
C	93.08	95.85
CLE1	93.08	98.62
CLE2	88.93	100.69
CLE3	93.08	100.00
CLE4	86.85	97.23
CLE5	2.42	3.11
Total	2.42	100.69

ANOVA

CCK8OD

	Sum of Squares	df	Mean Square	F	Sig.
Between Groups	21343.506	5	4268.701	280.976	.000
Within Groups	182.309	12	15.192		
Total	21525.815	17			

Post Hoc Tests

Multiple Comparisons

Dependent Variable: CCK8OD

	(I) Groups	(J) Groups	Mean Difference (I-J)	Std. Error	Sig.	95% Confidence Interval	
						Lower Bound	Upper Bound
LSD	C	CLE1	-.69204	3.18249	.832	-7.6261	6.2420
		CLE2	.46137	3.18249	.887	-6.4727	7.3954
		CLE3	-1.61476	3.18249	.621	-8.5488	5.3193
		CLE4	1.38408	3.18249	.671	-5.5500	8.3181
		CLE5	92.27220*	3.18249	.000	85.3381	99.2063
	CLE1	C	.69204	3.18249	.832	-6.2420	7.6261
		CLE2	1.15341	3.18249	.723	-5.7806	8.0875
		CLE3	-.92272	3.18249	.777	-7.8568	6.0113
		CLE4	2.07613	3.18249	.526	-4.8579	9.0102
		CLE5	92.96424*	3.18249	.000	86.0302	99.8983
	CLE2	C	-.46137	3.18249	.887	-7.3954	6.4727
		CLE1	-1.15341	3.18249	.723	-8.0875	5.7806
		CLE3	-2.07613	3.18249	.526	-9.0102	4.8579
		CLE4	.92271	3.18249	.777	-6.0113	7.8568
		CLE5	91.81083*	3.18249	.000	84.8768	98.7449
	CLE3	C	1.61476	3.18249	.621	-5.3193	8.5488
		CLE1	.92272	3.18249	.777	-6.0113	7.8568
		CLE2	2.07613	3.18249	.526	-4.8579	9.0102
		CLE4	2.99885	3.18249	.365	-3.9352	9.9329
		CLE5	93.88696*	3.18249	.000	86.9529	100.8210
	CLE4	C	-1.38408	3.18249	.671	-8.3181	5.5500
		CLE1	-2.07613	3.18249	.526	-9.0102	4.8579
		CLE2	-.92271	3.18249	.777	-7.8568	6.0113
		CLE3	-2.99885	3.18249	.365	-9.9329	3.9352
		CLE5	90.88812*	3.18249	.000	83.9541	97.8222
	CLE5	C	-92.27220*	3.18249	.000	-99.2063	-85.3381
		CLE1	-92.96424*	3.18249	.000	-99.8983	-86.0302
		CLE2	-91.81083*	3.18249	.000	-98.7449	-84.8768
		CLE3	-93.88696*	3.18249	.000	-100.8210	-86.9529
		CLE4	-90.88812*	3.18249	.000	-97.8222	-83.9541

*. The mean difference is significant at the 0.05 level.

Homogeneous Subsets

CCK8OD

	Groups	N	Subset for alpha = 0.05	
			1	2
Student-Newman-Keuls[a]	CLE5	3	2.6528	
	CLE4	3		93.5409
	CLE2	3		94.4637
	C	3		94.9250
	CLE1	3		95.6171
	CLE3	3		96.5398
	Sig.		1.000	.875

Means for groups in homogeneous subsets are displayed.

a. Uses Harmonic Mean Sample Size = 3.000.
