# Supplementary material for: Clemastine Fumarate Attenuates Myocardial Ischemia Reperfusion Injury Through Inhibition of Mast Cell Degranulation
Source: Front Pharmacol. 2021 Aug 27;12:704852. doi: 10.3389/fphar.2021.704852 (PMC8430029; doi:10.3389/fphar.2021.704852)
Supplement: Supplementary file 1 [file DataSheet1.ZIP › supplementary/Data Analysis/Figure 7A-D.pdf]

Oneway

Descriptives

		N	Mean	Std. Deviation	Std. Error	95% Confidence Interval for Mean Lower Bound
FIG.7A	Con	6	100.2717	.42353	.17290	99.8272
	C48/80 0.5h	6	98.4133	5.13036	2.09446	93.0294
	C48/80 2h	6	89.8283	4.19194	1.71135	85.4292
	C48/80 4h	6	89.7217	3.79464	1.54915	85.7394
	Total	24	94.5587	6.08624	1.24235	91.9888
FIG.7B	Con	3	.3113	.53925	.31133	-1.0282
	C48/80 0.5h	3	.3400	.44978	.25968	-.7773
	C48/80 2h	3	2.2400	.53226	.30730	.9178
	C48/80 4h	3	10.0333	.41633	.24037	8.9991
	Total	12	3.2312	4.20290	1.21327	.5608
FIG.7C	Con	6	100.3733	.51376	.20974	99.8342
	C48/80 0.5h	6	91.5617	3.17752	1.29722	88.2271
	C48/80 2h	6	90.8600	4.43664	1.81125	86.2040
	C48/80 4h	6	88.3517	4.61642	1.88465	83.5070
	Total	24	92.7867	5.71579	1.16673	90.3731
FIG.7D	Con	6	.3117	.37392	.15265	-.0807
	C48/80 0.5h	6	17.3717	1.67892	.68542	15.6097
	C48/80 2h	6	19.8433	2.22846	.90976	17.5047
	C48/80 4h	6	23.6100	1.39502	.56951	22.1460
	Total	24	15.2842	9.23412	1.88491	11.3849

Descriptives

		95% Confidence Interval for Mean Upper Bound	Minimum	Maximum
FIG.7A	Con	100.7161	100.00	100.89
	C48/80 0.5h	103.7973	93.02	106.68
	C48/80 2h	94.2275	86.33	95.56
	C48/80 4h	93.7039	86.82	96.44
	Total	97.1287	86.33	106.68
FIG.7B	Con	1.6509	.00	.93
	C48/80 0.5h	1.4573	.00	.85
	C48/80 2h	3.5622	1.87	2.85

	C48/80 4h	11.0676	9.70	10.50
	Total	5.9016	.00	10.50
FIG.7C	Con	100.9125	100.00	101.30
	C48/80 0.5h	94.8963	88.44	97.42
	C48/80 2h	95.5160	82.92	94.71
	C48/80 4h	93.1963	81.36	94.66
	Total	95.2002	81.36	101.30
FIG.7D	Con	.7041	.00	.95
	C48/80 0.5h	19.1336	15.97	20.62
	C48/80 2h	22.1820	17.43	22.97
	C48/80 4h	25.0740	22.02	25.26
	Total	19.1834	.00	25.26

ANOVA

		Sum of Squares	df	Mean Square	F	Sig.
FIG.7A	Between Groups	559.617	3	186.539	12.761	.000
	Within Groups	292.358	20	14.618		
	Total	851.975	23			
FIG.7B	Between Groups	192.408	3	64.136	270.127	.000
	Within Groups	1.899	8	.237		
	Total	194.308	11			
FIG.7C	Between Groups	494.636	3	164.879	12.842	.000
	Within Groups	256.778	20	12.839		
	Total	751.415	23			
FIG.7D	Between Groups	1911.833	3	637.278	258.250	.000
	Within Groups	49.353	20	2.468		
	Total	1961.187	23			

Post Hoc Tests

Multiple Comparisons

Dependent Variable	(I) Groups	(J) Groups	Mean Difference (I-J)	Std. Error	Sig.	95% Confidence Interval	
						Lower Bound	Upper Bound

FIG.7A	LSD	Con	C48/80 0.5h	1.85833	2.20740	.410	-2.7462	6.4629
			C48/80 2h	10.44333*	2.20740	.000	5.8388	15.0479
			C48/80 4h	10.55000*	2.20740	.000	5.9454	15.1546
		C48/80 0.5h	Con	-1.85833	2.20740	.410	-6.4629	2.7462
			C48/80 2h	8.58500*	2.20740	.001	3.9804	13.1896
			C48/80 4h	8.69167*	2.20740	.001	4.0871	13.2962
		C48/80 2h	Con	-10.44333*	2.20740	.000	-15.0479	-5.8388
			C48/80 0.5h	-8.58500*	2.20740	.001	-13.1896	-3.9804
			C48/80 4h	.10667	2.20740	.962	-4.4979	4.7112
		C48/80 4h	Con	-10.55000*	2.20740	.000	-15.1546	-5.9454
			C48/80 0.5h	-8.69167*	2.20740	.001	-13.2962	-4.0871
			C48/80 2h	-.10667	2.20740	.962	-4.7112	4.4979
FIG.7B	LSD	Con	C48/80 0.5h	-.02867	.39785	.944	-.9461	.8888
			C48/80 2h	-1.92867*	.39785	.001	-2.8461	-1.0112
			C48/80 4h	-9.72200*	.39785	.000	-10.6394	-8.8046
		C48/80 0.5h	Con	.02867	.39785	.944	-.8888	.9461
			C48/80 2h	-1.90000*	.39785	.001	-2.8174	-.9826
			C48/80 4h	-9.69333*	.39785	.000	-10.6108	-8.7759
		C48/80 2h	Con	1.92867*	.39785	.001	1.0112	2.8461
			C48/80 0.5h	1.90000*	.39785	.001	.9826	2.8174
			C48/80 4h	-7.79333*	.39785	.000	-8.7108	-6.8759
		C48/80 4h	Con	9.72200*	.39785	.000	8.8046	10.6394
			C48/80 0.5h	9.69333*	.39785	.000	8.7759	10.6108
			C48/80 2h	7.79333*	.39785	.000	6.8759	8.7108
FIG.7C	LSD	Con	C48/80 0.5h	8.81167*	2.06873	.000	4.4964	13.1270
			C48/80 2h	9.51333*	2.06873	.000	5.1980	13.8286
			C48/80 4h	12.02167*	2.06873	.000	7.7064	16.3370
		C48/80 0.5h	Con	-8.81167*	2.06873	.000	-13.1270	-4.4964
			C48/80 2h	.70167	2.06873	.738	-3.6136	5.0170
			C48/80 4h	3.21000	2.06873	.136	-1.1053	7.5253
		C48/80 2h	Con	-9.51333*	2.06873	.000	-13.8286	-5.1980
			C48/80 0.5h	-.70167	2.06873	.738	-5.0170	3.6136
			C48/80 4h	2.50833	2.06873	.239	-1.8070	6.8236
		C48/80 4h	Con	-12.02167*	2.06873	.000	-16.3370	-7.7064
			C48/80 0.5h	-3.21000	2.06873	.136	-7.5253	1.1053
			C48/80 2h	-2.50833	2.06873	.239	-6.8236	1.8070
FIG.7D	LSD	Con	C48/80 0.5h	-17.06000*	.90695	.000	-18.9519	-15.1681
			C48/80 2h	-19.53167*	.90695	.000	-21.4235	-17.6398
			C48/80 4h	-23.29833*	.90695	.000	-25.1902	-21.4065
		C48/80 0.5h	Con	17.06000*	.90695	.000	15.1681	18.9519

		C48/80 2h	-2.47167*	.90695	.013	-4.3635	-.5798
		C48/80 4h	-6.23833*	.90695	.000	-8.1302	-4.3465
	C48/80 2h	Con	19.53167*	.90695	.000	17.6398	21.4235
		C48/80 0.5h	2.47167*	.90695	.013	.5798	4.3635
		C48/80 4h	-3.76667*	.90695	.000	-5.6585	-1.8748
	C48/80 4h	Con	23.29833*	.90695	.000	21.4065	25.1902
		C48/80 0.5h	6.23833*	.90695	.000	4.3465	8.1302
		C48/80 2h	3.76667*	.90695	.000	1.8748	5.6585

*. The mean difference is significant at the 0.05 level.

Homogeneous Subsets

FIG.7A

	Groups	N	Subset for alpha = 0.05	
			1	2
Student-Newman-Keuls[a]	C48/80 4h	6	89.7217	
	C48/80 2h	6	89.8283	
	C48/80 0.5h	6		98.4133
	Con	6		100.2717
	Sig.		.962	.410

Means for groups in homogeneous subsets are displayed.

a. Uses Harmonic Mean Sample Size = 6.000.

FIG.7B

	Groups	N	Subset for alpha = 0.05		
			1	2	3
Student-Newman-Keuls[a]	Con	3	.3113		
	C48/80 0.5h	3	.3400		
	C48/80 2h	3		2.2400	
	C48/80 4h	3			10.0333
	Sig.		.944	1.000	1.000

Means for groups in homogeneous subsets are displayed.

a. Uses Harmonic Mean Sample Size = 3.000.

FIG.7C

	Groups	N	Subset for alpha = 0.05	
			1	2
Student-Newman-Keuls[a]	C48/80 4h	6	88.3517	
	C48/80 2h	6	90.8600	
	C48/80 0.5h	6	91.5617	
	Con	6		100.3733
	Sig.		.289	1.000

Means for groups in homogeneous subsets are displayed.

a. Uses Harmonic Mean Sample Size = 6.000.

FIG.7D

	Groups	N	Subset for alpha = 0.05			
			1	2	3	4
Student-Newman-Keuls[a]	Con	6	.3117			
	C48/80 0.5h	6		17.3717		
	C48/80 2h	6			19.8433	
	C48/80 4h	6				23.6100
	Sig.		1.000	1.000	1.000	1.000

Means for groups in homogeneous subsets are displayed.

a. Uses Harmonic Mean Sample Size = 6.000.
