# Supplementary material for: Clemastine Fumarate Attenuates Myocardial Ischemia Reperfusion Injury Through Inhibition of Mast Cell Degranulation
Source: Front Pharmacol. 2021 Aug 27;12:704852. doi: 10.3389/fphar.2021.704852 (PMC8430029; doi:10.3389/fphar.2021.704852)
Supplement: Supplementary file 1 [file DataSheet1.ZIP › supplementary/Data Analysis/Figure 7E-K.pdf]

Oneway

Descriptives

		N	Mean	Std. Deviation	Std. Error	95% Confidence Interval for Mean Lower Bound
FIG.E	Con	5	102.3580	1.68727	.75457	100.2630
	C48/80 0.5h	5	83.3260	2.22157	.99352	80.5676
	CLE+C48/80 0.5h	5	88.4160	.67774	.30309	87.5745
	Total	15	91.3667	8.46762	2.18633	86.6775
FIG.F	Con	5	.1460	.11824	.05288	-.0008
	C48/80 0.5h	5	59.1140	1.40390	.62784	57.3708
	CLE+C48/80 0.5h	5	31.3040	.58641	.26225	30.5759
	Total	15	30.1880	24.94525	6.44084	16.3738
FIG.H	Con	3	.0033	.00577	.00333	-.0110
	C48/80 0.5h	3	.5133	.04163	.02404	.4099
	CLE+C48/80 0.5h	3	.1867	.04041	.02333	.0863
	Total	9	.2344	.22562	.07521	.0610
FIG.J	Con	3	2.9033	.31817	.18370	2.1130
	C48/80 0.5h	3	.2100	.06000	.03464	.0610
	CLE+C48/80 0.5h	3	1.9067	.21127	.12197	1.3819
	Total	9	1.6733	1.19504	.39835	.7547
FIG.K	Con	3	1.5500	.15620	.09018	1.1620
	C48/80 0.5h	3	2.3833	.21825	.12601	1.8412
	CLE+C48/80 0.5h	3	1.6933	.11590	.06692	1.4054
	Total	9	1.8756	.41262	.13754	1.5584

Descriptives

		95% Confidence Interval for Mean Upper Bound	Minimum	Maximum
FIG.E	Con	104.4530	100.00	104.57
	C48/80 0.5h	86.0844	79.79	85.40
	CLE+C48/80 0.5h	89.2575	87.71	89.54
	Total	96.0559	79.79	104.57
FIG.F	Con	.2928	.00	.32
	C48/80 0.5h	60.8572	56.89	60.69
	CLE+C48/80 0.5h	32.0321	30.62	32.00
	Total	44.0022	.00	60.69
FIG.H	Con	.0177	.00	.01
	C48/80 0.5h	.6168	.48	.56

	CLE+C48/80 0.5h	.2871	.15	.23
	Total	.4079	.00	.56
FIG.J	Con	3.6937	2.70	3.27
	C48/80 0.5h	.3590	.15	.27
	CLE+C48/80 0.5h	2.4315	1.71	2.13
	Total	2.5919	.15	3.27
FIG.K	Con	1.9380	1.37	1.65
	C48/80 0.5h	2.9255	2.19	2.62
	CLE+C48/80 0.5h	1.9813	1.57	1.80
	Total	2.1927	1.37	2.62

ANOVA

		Sum of Squares	df	Mean Square	F	Sig.
FIG.E	Between Groups	970.841	2	485.420	176.697	.000
	Within Groups	32.966	12	2.747		
	Total	1003.807	14			
FIG.F	Between Groups	8702.403	2	4351.202	5605.317	.000
	Within Groups	9.315	12	.776		
	Total	8711.719	14			
FIG.H	Between Groups	.400	2	.200	176.657	.000
	Within Groups	.007	6	.001		
	Total	.407	8			
FIG.J	Between Groups	11.126	2	5.563	111.658	.000
	Within Groups	.299	6	.050		
	Total	11.425	8			
FIG.K	Between Groups	1.191	2	.596	20.904	.002
	Within Groups	.171	6	.028		
	Total	1.362	8			

Post Hoc Tests

Multiple Comparisons

Dependent Variable	(I) Groups	(J) Groups	Mean Difference	Std. Error	Sig.

				(I-J)		
FIG.E	LSD	Con	C48/80 0.5h	19.03200*	1.04827	.000
			CLE+C48/80 0.5h	13.94200*	1.04827	.000
		C48/80 0.5h	Con	-19.03200*	1.04827	.000
			CLE+C48/80 0.5h	-5.09000*	1.04827	.000
		CLE+C48/80 0.5h	Con	-13.94200*	1.04827	.000
			C48/80 0.5h	5.09000*	1.04827	.000
FIG.F	LSD	Con	C48/80 0.5h	-58.96800*	.55723	.000
			CLE+C48/80 0.5h	-31.15800*	.55723	.000
		C48/80 0.5h	Con	58.96800*	.55723	.000
			CLE+C48/80 0.5h	27.81000*	.55723	.000
		CLE+C48/80 0.5h	Con	31.15800*	.55723	.000
			C48/80 0.5h	-27.81000*	.55723	.000
FIG.H	LSD	Con	C48/80 0.5h	-.51000*	.02749	.000
			CLE+C48/80 0.5h	-.18333*	.02749	.001
		C48/80 0.5h	Con	.51000*	.02749	.000
			CLE+C48/80 0.5h	.32667*	.02749	.000
		CLE+C48/80 0.5h	Con	.18333*	.02749	.001
			C48/80 0.5h	-.32667*	.02749	.000
FIG.J	LSD	Con	C48/80 0.5h	2.69333*	.18225	.000
			CLE+C48/80 0.5h	.99667*	.18225	.002
		C48/80 0.5h	Con	-2.69333*	.18225	.000
			CLE+C48/80 0.5h	-1.69667*	.18225	.000
		CLE+C48/80 0.5h	Con	-.99667*	.18225	.002
			C48/80 0.5h	1.69667*	.18225	.000
FIG.K	LSD	Con	C48/80 0.5h	-.83333*	.13781	.001
			CLE+C48/80 0.5h	-.14333	.13781	.338
		C48/80 0.5h	Con	.83333*	.13781	.001
			CLE+C48/80 0.5h	.69000*	.13781	.002
		CLE+C48/80 0.5h	Con	.14333	.13781	.338
			C48/80 0.5h	-.69000*	.13781	.002

Multiple Comparisons

Dependent Variable		(I) Groups	(J) Groups	95% Confidence Interval Lower Bound	Upper Bound
FIG.E	LSD	Con	C48/80 0.5h	16.7480	21.3160
			CLE+C48/80 0.5h	11.6580	16.2260
		C48/80 0.5h	Con	-21.3160	-16.7480
			CLE+C48/80 0.5h	-7.3740	-2.8060
		CLE+C48/80 0.5h	Con	-16.2260	-11.6580
			C48/80 0.5h	2.8060	7.3740

FIG.F	LSD	Con	C48/80 0.5h	-60.1821	-57.7539
			CLE+C48/80 0.5h	-32.3721	-29.9439
		C48/80 0.5h	Con	57.7539	60.1821
			CLE+C48/80 0.5h	26.5959	29.0241
		CLE+C48/80 0.5h	Con	29.9439	32.3721
			C48/80 0.5h	-29.0241	-26.5959
FIG.H	LSD	Con	C48/80 0.5h	-.5773	-.4427
			CLE+C48/80 0.5h	-.2506	-.1161
		C48/80 0.5h	Con	.4427	.5773
			CLE+C48/80 0.5h	.2594	.3939
		CLE+C48/80 0.5h	Con	.1161	.2506
			C48/80 0.5h	-.3939	-.2594
FIG.J	LSD	Con	C48/80 0.5h	2.2474	3.1393
			CLE+C48/80 0.5h	.5507	1.4426
		C48/80 0.5h	Con	-3.1393	-2.2474
			CLE+C48/80 0.5h	-2.1426	-1.2507
		CLE+C48/80 0.5h	Con	-1.4426	-.5507
			C48/80 0.5h	1.2507	2.1426
FIG.K	LSD	Con	C48/80 0.5h	-1.1706	-.4961
			CLE+C48/80 0.5h	-.4806	.1939
		C48/80 0.5h	Con	.4961	1.1706
			CLE+C48/80 0.5h	.3528	1.0272
		CLE+C48/80 0.5h	Con	-.1939	.4806
			C48/80 0.5h	-1.0272	-.3528

*. The mean difference is significant at the 0.05 level.

Homogeneous Subsets

FIG.E

			Subset for alpha = 0.05		
	Groups	N	1	2	3
Student-Newman-Keuls[a]	C48/80 0.5h	5	83.3260		
	CLE+C48/80 0.5h	5		88.4160	
	Con	5			102.3580
	Sig.		1.000	1.000	1.000

Means for groups in homogeneous subsets are displayed.

a. Uses Harmonic Mean Sample Size = 5.000.

FIG.F

	Groups	N	Subset for alpha = 0.05		
			1	2	3
Student-Newman-Keuls[a]	Con	5	.1460		
	CLE+C48/80 0.5h	5		31.3040	
	C48/80 0.5h	5			59.1140
	Sig.		1.000	1.000	1.000

Means for groups in homogeneous subsets are displayed.

a. Uses Harmonic Mean Sample Size = 5.000.

FIG.H

	Groups	N	Subset for alpha = 0.05		
			1	2	3
Student-Newman-Keuls[a]	Con	3	.0033		
	CLE+C48/80 0.5h	3		.1867	
	C48/80 0.5h	3			.5133
	Sig.		1.000	1.000	1.000

Means for groups in homogeneous subsets are displayed.

a. Uses Harmonic Mean Sample Size = 3.000.

FIG.J

	Groups	N	Subset for alpha = 0.05		
			1	2	3
Student-Newman-Keuls[a]	C48/80 0.5h	3	.2100		
	CLE+C48/80 0.5h	3		1.9067	
	Con	3			2.9033
	Sig.		1.000	1.000	1.000

Means for groups in homogeneous subsets are displayed.

a. Uses Harmonic Mean Sample Size = 3.000.

FIG.K

Groups	N	Subset for alpha = 0.05

			1	2
Student-Newman-Keuls[a]	Con	3	1.5500	
	CLE+C48/80 0.5h	3	1.6933	
	C48/80 0.5h	3		2.3833
	Sig.		.338	1.000

Means for groups in homogeneous subsets are displayed.

a. Uses Harmonic Mean Sample Size = 3.000.
