# Supplementary material for: Clemastine Fumarate Attenuates Myocardial Ischemia Reperfusion Injury Through Inhibition of Mast Cell Degranulation
Source: Front Pharmacol. 2021 Aug 27;12:704852. doi: 10.3389/fphar.2021.704852 (PMC8430029; doi:10.3389/fphar.2021.704852)
Supplement: Supplementary file 1 [file DataSheet1.ZIP › supplementary/Supplementary Image.pdf]

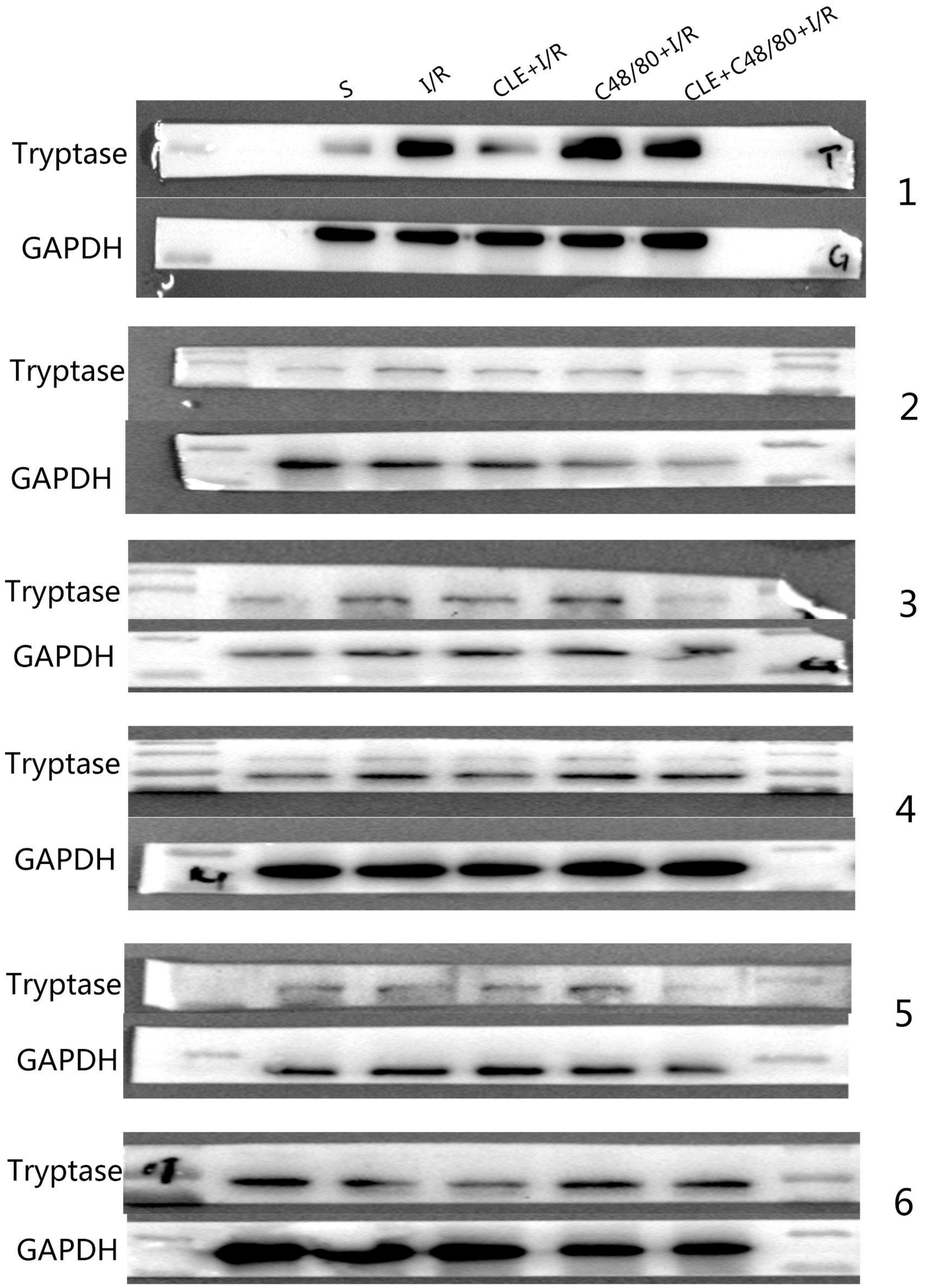

Supplementary 1. WB of tryptase protein in heart tissue.

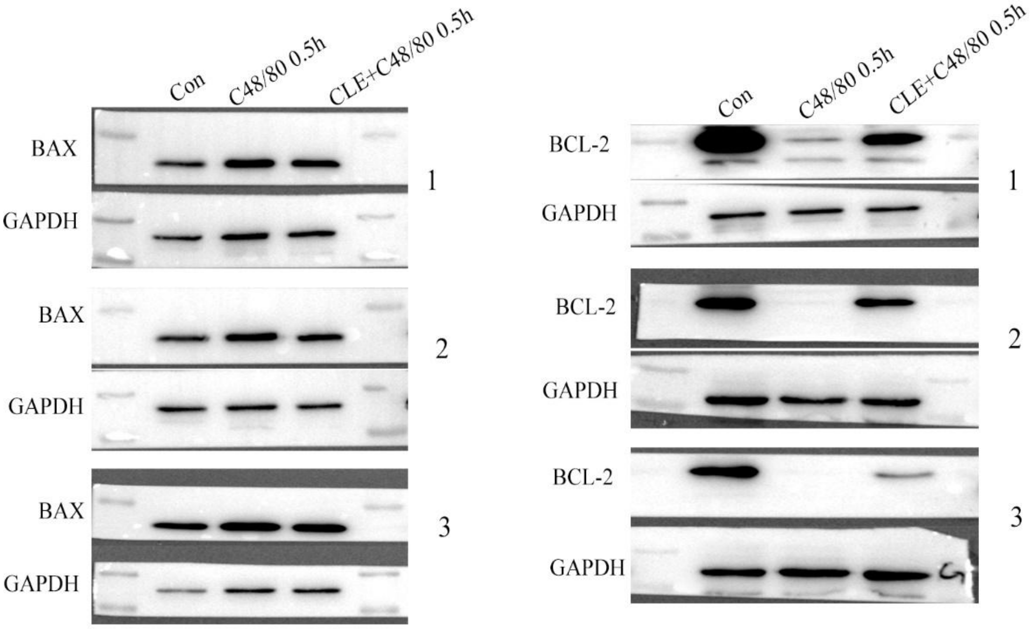

Supplementary 2. WB of BAX and BCl-2 protein in H9C2 cells .

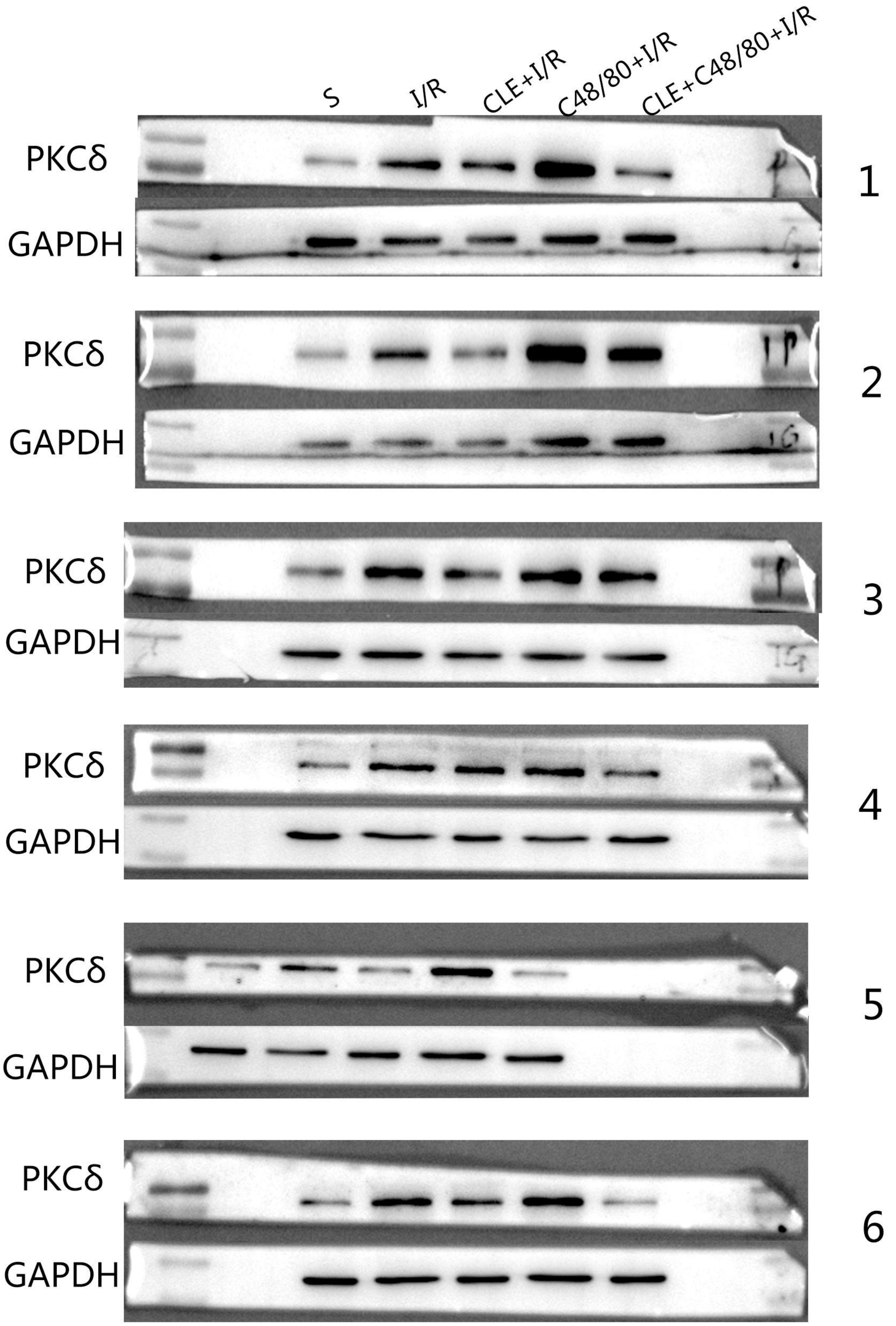

Supplementary 3. WB of δPKC protein in heart tissue.

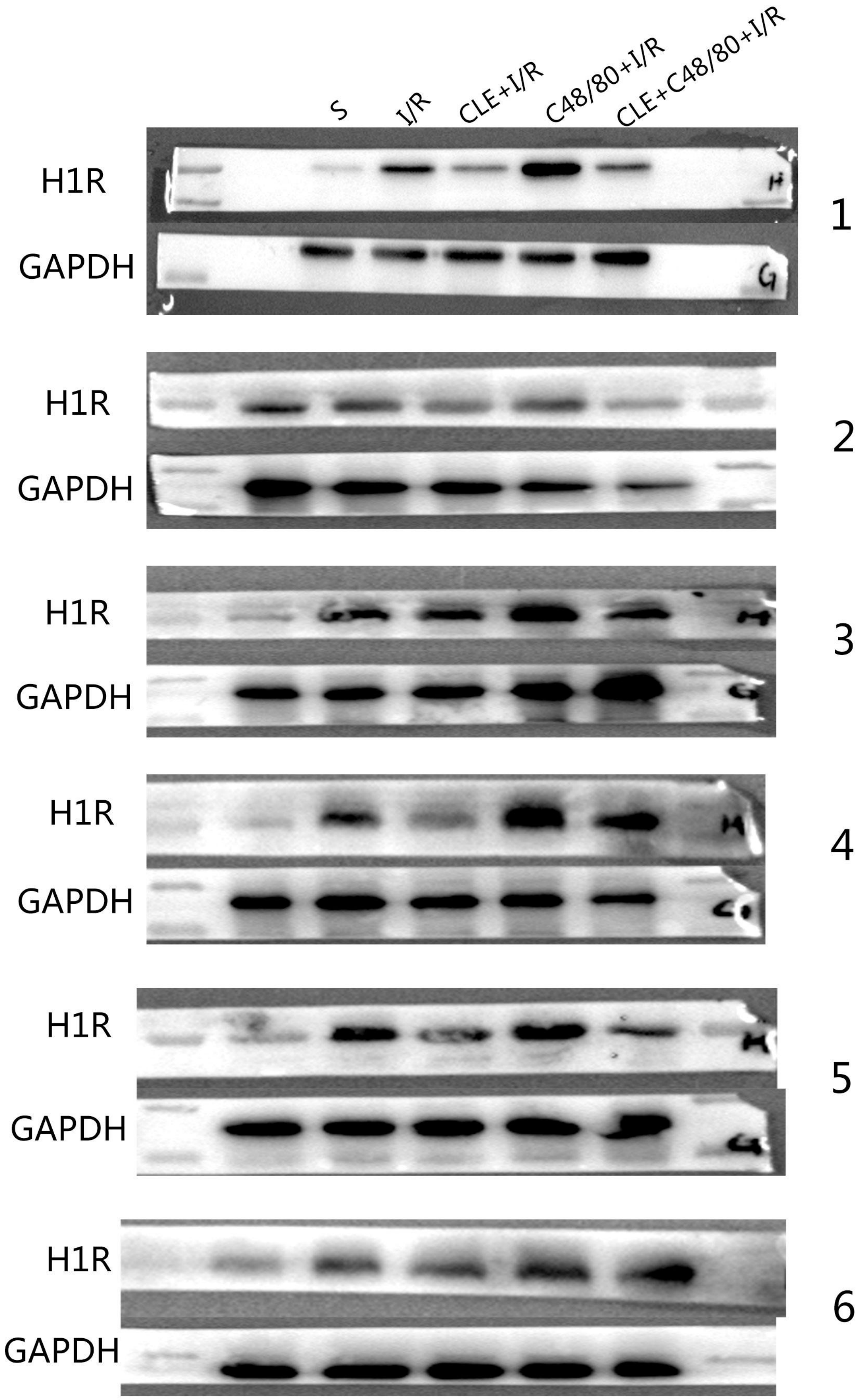

Supplementary 4. WB of H1R protein in heart tissue.
